# Are neuroanatomy multimedia resources effective in improving medical students' understanding of structural and functional neuroanatomy? A systematic review

**DOI:** 10.1002/ase.70079

**Published:** 2025-08-20

**Authors:** Eleni Patera, Mark Pickering, Thomas Flanagan

**Affiliations:** ^1^ Department of Anatomy, School of Medicine University College Dublin Dublin Ireland; ^2^ Human Anatomy Resource Centre University of Liverpool Liverpool UK

**Keywords:** medical students, multimedia, neuroanatomy education, neuroanatomy resources, pre‐clinical medical education

## Abstract

Neuroanatomy education has evolved and improved over the years, driven by advances in technology that have led to the development of innovative interactive digital and immersive learning resources. While neuroanatomy educators have been keeping pace with and harnessing these technology advances, many medical students still struggle to apply their basic neuroanatomy knowledge in a clinical context. Furthermore, the effectiveness of multimedia resources in improving medical students' understanding of structural and functional neuroanatomy has received limited attention. A systematic review was conducted to document neuroanatomy multimedia resources that were designed for pre‐clinical medical students and assessed their effectiveness in improving medical students' understanding of structural and functional neuroanatomy. Twenty‐nine articles were eligible to address the study objective and were appraised according to PRISMA‐P guidelines. This review concluded that neuroanatomy multimedia resources are primarily used as supplementary learning tools. However, due to a lack of robust evidence, no definitive conclusions can be made about their effectiveness in enhancing students' understanding of neuroanatomy. Nevertheless, this systematic review found that most neuroanatomy multimedia resources are effective in supporting students' understanding of structural neuroanatomy but less so for functional and clinical neuroanatomy. Additionally, static multimedia elements were more prevalent than dynamic ones. Moving forward, the thoughtful and informed use of multimedia elements could help establish resources that better integrate structural and functional neuroanatomy with their clinical and real‐life applications, ultimately bridging the gap between neuroanatomy and clinical neurology.

## INTRODUCTION

A historical review of neuroanatomy throughout the centuries serves as a reminder of how central the subject of neuroanatomy is to the disciplines of anatomy and clinical neurosciences.^1^ Knowledge of both structural and functional neuroanatomy is essential for applying knowledge in clinical neuroanatomy, which is concerned with how injury and disease can result in neurologic deficits.^2^ Neuroanatomy has been characterized as one of the most challenging components of the anatomy curriculum due to the inherent complexity of specific neuroanatomy concepts and associated neuroanatomical structures.^3^, ^4^, ^5^, ^6^ Remarkably, neuroanatomy has its own individual place in a medical curriculum, and it is often taught separately from gross anatomy courses as a stand‐alone course, with a specific amount of teaching hours dedicated to it and its associated laboratory practicals.^7^, ^8^ In recent years, concerns have been raised within the medical profession and medical education about students' inadequate knowledge of neuroanatomy.^7^, ^9^ Retention of key neuroanatomy concepts and clinically relevant principles is essential not only for physicians interested in specializing in neurology or neurosurgery, but also for general practitioners who must conduct standard clinical examinations and determine when a patient requires referral to a neurology specialist.^10^, ^11^ In addition, there has been a notable reduction in neuroanatomy teaching hours within medical curricula.^8^, ^12^, ^13^ Despite this, it remains unclear whether students' limited exposure to neuroanatomy directly leads to weaker knowledge of the subject and whether this, in turn, contributes to junior doctors feeling unprepared to manage neurological conditions.

In 1994, American professor of neurology, Ralph Jozefowicz, introduced the concept of “*neurophobia*,” which he defined as “a fear of the neural sciences and clinical neurology that is due to the students' inability to apply their knowledge of basic sciences to clinical situations”.^14^ Since the introduction of the concept of neurophobia, extensive research has been conducted to identify its causes, the factors contributing to difficulties in learning and understanding neuroanatomy, and the educational interventions that could help mitigate neurophobia.^4^, ^15^, ^16^, ^17^, ^18^, ^19^, ^20^, ^21^ In the future, the burden of neurological diseases is expected to intensify as major disorders affecting the nervous system continue to rise.^22^ The failure to recognize the implications of neurophobia in medical education could lead to a shortfall of practising neurologists in the future.^19^

Medical educators are facing growing pressure to address neurophobia by reforming neurology education, given its perceived impact on discouraging medical students from pursuing neurology as a career specialty. In 2021, Omar discussed various teaching strategies that could help tackle neurophobia.^23^ Some strategies include horizontal or vertical integration in medical education.^23^ Furthermore, a study showed that vertical integration in neuroanatomy teaching resulted in students feeling more prepared for clinical neuroscience‐related concepts (e.g., interpreting MRI and CT images) once they entered the clinical years of their degree.^24^

### Impact of technological advances on neuroanatomy education

Advances in educational technology have significantly transformed neuroanatomy instruction, shifting from traditional methods to modern, technology‐based teaching approaches and resources.^25^ New technologies (e.g., synchronous or asynchronous digital classrooms, virtual learning environments, and online interaction tools) are promising, offering the potential to simplify students' access to teaching and learning materials, enabling self‐paced learning, and enhancing the overall learning experience.^26^, ^27^

In recent decades, online learning, defined as education that occurs over the Internet,^28^ has been incorporated into neuroanatomy education. The terms “e‐learning” (electronic learning) and “computer‐assisted learning” (CAL) are often used interchangeably with online learning. Multimedia CAL resources have gained increased attention in recent years.^29^, ^30^ Multimedia technology is defined as “the use of a computer to present and combine graphics, audio, and video, with links and tools that let the user navigate, interact, create, and communicate”.^31^ Neuroanatomy educators can use multimedia elements such as audio, images, text, and videos during instruction, potentially simplifying complex concepts for students.

Advances in technology have led to the creation of both non‐digital resources, such as 3D‐printed and 3D plastic models, and digital resources ranging from simple multimedia tools to more complex immersive technologies such as virtual reality (VR), augmented reality (AR), mixed reality (MR), stereoscopy, and 3D digital atlases.^25^ Despite these advances, educators must exercise caution when integrating such technologies into their teaching. Educators need to ensure that any technological resource used for instruction or assigned to students is not overly complex and that students have sufficient time to engage with the resource. Insufficient exposure to these resources may hinder students' learning and their ability to engage effectively. When designing a resource, educators must ensure it is purposefully aligned with its intended goals. Key questions to consider include (1) What do students find challenging and why? (2) What features are required to simplify a topic? (3) How does the resource enhance the students' learning experience? (4) Does the resource content constructively align with the learning objectives on which students will be assessed?

### Research focused on the neuroanatomy teaching methods, tools and their impact on students' learning

Currently, in the literature, there are three systematic reviews focusing purely on neuroanatomy education. In 2018, Arantes et al. published a systematic review to explore neuroanatomy teaching methods and assess their impact on learning, with the ultimate goal of providing guidance for curricular improvement.^32^ This systematic review included 29 articles for final analysis and identified 15 teaching methods that were classified as digital and non‐digital tools. The number of articles focusing on digital tools and non‐digital tools was 14 for both categories. Most of the six studies focusing on computer‐based digital tools found that these tools were effective for both students and faculty, with students showing a positive attitude toward using them and demonstrating improved performance in their quiz or test scores. However, two studies revealed no statistically significant differences in students' scores after the introduction of computers into the course. Furthermore, students reported lower scores on deep approach for the computer‐based course compared to general studies (CAL Course: 25.55; General studies: 27.44). While this systematic review included studies involving medical students and students from allied health professions, a limitation was that it did not include research on the use of virtual or augmented reality in neuroanatomy education.

In 2020, Sotgiu et al. published a systematic review aimed at identifying the most effective methods for teaching human neuroanatomy, focusing on the challenges it poses for medical students.^5^ Newman et al. pointed out a major limitation of the review by Sotgiu et al. noting that it did not include any studies discussing VR or AR modalities.^33^ While few in number, Sotgiu et al. did include two studies in their review—one focussing on VR and the other on mobile augmented reality (mAR).^5^ However, as Chytas et al.^34^ highlighted, a major limitation of this review is that, despite the assertion of Sotgiu et al.^5^ that a combination of teaching methods is needed in neuroanatomy education, the authors concluded that cadaveric dissection remains the “gold standard” method for teaching neuroanatomy. Chytas et al.^34^ argued that this conclusion was not evidence‐based, as only two out of the 18 studies included in the review focused on cadaveric dissection. Moreover, while Sotgiu et al.^5^ referenced various studies that showed positive outcomes favoring cadaveric dissection, none specifically examined its effectiveness in neuroanatomy education specifically. Furthermore, the results did not demonstrate that cadaveric dissection leads to superior knowledge acquisition compared to other educational methods.^34^

In 2022, Newman et al.^33^ conducted a focused review to explore the technology‐enhanced teaching methods currently available to neuroanatomy educators. Their review aimed to compare traditional teaching methods with technology‐based approaches, identify those associated with improved knowledge acquisition and long‐term retention, and understand why some teaching methods are effective in particular contexts while others are not. Newman et al.^33^ noted that the systematic reviews by Arantes et al.^32^ and Sotgiu et al.^5^ lacked adequate descriptions of the resources they examined and failed to provide sufficient context to explain why certain resources yielded better outcomes than others. In response, Newman et al.^33^ proposed four potential explanations for why certain resources appear more effective in helping students to acquire and retain neuroanatomy knowledge over time. However, a potential limitation of their review is that it did not include CAL as one of the technology‐enhanced teaching methods for neuroanatomy education.

Current evidence in the literature demonstrates that there are various innovative tools, resources, and teaching methods available for neuroanatomy education. Despite this, the use of complex technological equipment does not automatically lead to better learning outcomes. There has been insufficient focus on the effectiveness of multimedia resources in improving medical students' understanding of both structural and functional neuroanatomy. It remains unclear whether these technology‐enhanced neuroanatomy resources are designed in a way that promotes deep learning while also helping students apply their knowledge of basic sciences in clinical contexts.

A systematic review that evaluates the effectiveness of neuroanatomy multimedia resources in improving medical students' understanding of neuroanatomy—by examining the outcomes of each study and identifying how different components of the resources support student learning—has not yet been published. Such a review would be valuable for neuroanatomy educators, as it could highlight potential inefficiencies in current neuroanatomy multimedia educational resources. This insight could help educators design more effective resources that aid students in mastering structural neuroanatomy, which forms the foundation for understanding functional neuroanatomy, ultimately supporting their progression through various levels of knowledge while using the resource.

The aim of this systematic review is to address the following research question: “Are neuroanatomy multimedia resources specifically designed for pre‐clinical medical students effective in improving their understanding of structural and functional neuroanatomy?”

## MATERIALS AND METHODS

### Protocol review

This systematic review was guided by the Preferred Reporting Items for Systematic Reviews and Meta‐Analyses Protocols (PRISMA‐P)^35^ guidelines.

### Literature search and databases

The bibliographic databases that were used for this systematic review were PubMed (United States National Library of Medicine, Bethesda, MD), Scopus™ (Elsevier B.V., Amsterdam, The Netherlands) and Education Resources Information Center (ERIC) (Institute of Education Sciences [IES], Washington, DC). The following combination of search strings was used to identify relevant studies: “neuroanatomy” AND (“learning” OR “e‐learning” OR “education” OR “teaching” OR “multimedia” OR “multi‐media” OR “video” OR “resource” OR “interactive” OR “animation” OR “virtual reality” OR “augmented reality” OR “online” OR “student”). No distinction was made in the term “neuroanatomy” to “structural” and “functional” as the use of the terms “structural neuroanatomy” OR “functional neuroanatomy” yielded results that focused on neurophysiology or neuropathology and not on neuroanatomy education. The comprehensive search strategy for the PubMed database can be seen in Appendix A.

The search utilized the “Article Type” filter on PubMed and Scopus, which was unavailable on the ERIC database. The filter was limited to journal articles, including original research, descriptive articles, book chapters, and short communications. Additionally, the “Publication Date” filter was applied to limit the search from 1994 to 2024. This date range was chosen because the concept of neurophobia was first introduced in 1994. Initially, only these two filters were applied during the electronic search to export the identified studies from each database and import them to the Covidence software (*Veritas Health Innovation, Melbourne, Australia*). Only studies published in English were considered, as this is the language of the authors. The study selection process began on September 24, 2021, and ended on December 31, 2024.

### Study selection criteria and screening procedures

The review followed the Patient/Population, Intervention, Comparison and Outcomes and Study (PICOS) model to define the criteria for study eligibility^36^: population (pre‐clinical undergraduate or graduate medical students), exposure (multimedia VR, AR, MR, or CAL resources), comparator (multimedia VR, AR, MR, CAL resources, or no comparator), outcomes (any multimedia neuroanatomy resource that was specifically designed for pre‐clinical medical students that assessed its impact on students' understanding of neuroanatomy), publication type (journal research articles and book chapters), study design ((e.g., randomized controlled trials (RCTs) and mixed‐methods)), language (English) and year of publication (April 1994 to December 2024). The inclusion and exclusion criteria for both study eligibility and report eligibility are demonstrated in Table 1.

**TABLE 1:** Inclusion and exclusion criteria for each domain of PICO(s) and other eligibility variables in relation to the research question.

PICO(s)	Inclusion	Exclusion
Population	Undergraduate or graduate pre‐clinical medical students regardless of country of education or participants' age	Clinical undergraduate or graduate medical students Undergraduate or postgraduate students studying any other major other than medicine Junior doctors Neurology/neurosurgery residents/trainees/physicians
Exposure	Neuroanatomy multimedia resources (varying from immersive technologies [VR, AR, mAR, MR, stereoscopy] to computer assisted learning (CAL) resources)	Digital resources without the use of multimedia Digital resources with extremely limited use of multimedia elements Gross anatomy multimedia resources Microanatomy/Histology multimedia resources
Comparator	Immersive technology other than the one used as the exposure (VR, AR, mAR, MR, stereoscopy) CAL resources However, no studies were excluded if a comparator was not used	N/A
Outcome	Any educational neuroanatomy multimedia resource that was specifically designed for pre‐clinical medical students and evaluated the effectiveness of the resource in improving pre‐clinical medical students' understanding of neuroanatomy	Any “physical” model (i.e., 3D printed model) Any multimedia model that relates to microanatomy/histology Dissection tools Any multimedia model that was not designed specifically for pre‐clinical medical students
Study design	Any study design was acceptable as long as it addressed the systematic review's research question and any other additional research questions	N/A
Publication type	Journal research articles Book chapters	Research articles behind a paywall Case reports Viewpoint commentaries Book chapters behind a paywall Letters to the editor Systematic reviews and meta‐analyses
Language	English	Any language other than English
Year of publication	April 1994 to December 2024	Articles published before April 1994

Abbreviations: 3D, three‐dimensional; AR, augmented reality; CAL, computer‐assisted‐learning; mAR, mobile augmented reality; MR, mixed reality; N/A, not applicable; PICO(s), Patient/Population, Intervention, Comparison and Outcomes and Study; VR, virtual reality.

Two independent reviewers (EP and TF) performed the study selection in two screening stages. First, the titles and abstracts of the articles were reviewed. Full texts were retrieved only when inclusion could not be determined from the title and abstract or if no abstract was available. In the second stage, full texts were assessed for inclusion. Discrepancies between reviewers were resolved by a third reviewer (MP). Articles excluded from the review were rejected by at least two of the three reviewers.

### Data extraction

The first author (EP) collected data from each included study. Key study characteristics such as the authors, publication year, country where study was conducted at, study design, participant cohort, study aims, type of multimedia resource and outcomes regarding resource's impact on students' understanding of neuroanatomy were tabulated in Table 2. The PICO(s) model was used to extract important information, including the study aim, design, population, exposure, comparator, measures, and outcomes (Table B1, Appendix B). The number of multimedia elements across each resource, such as images, text, audio, video, animations, and manipulable models (e.g., rotation and zooming), was also determined by reviewing descriptions in the articles, screenshots of the resource, supplementary materials, or by accessing the resources online. Bar charts were created to visually compare the multimedia elements present or absent in each resource. Thematic analysis was conducted after assessing the articles, identifying strengths and weaknesses in the resources and the corresponding studies. The strengths and weaknesses were identified predominantly based on the primary author's (EP) interpretation of the information presented within each study and resource if it was accessible, rather than being directly extracted verbatim. The interpretation focused on each study's materials and methods, results, discussion and conclusion. Recurring themes were noted across each study's methodological quality and clarity of result reporting. In cases where limitations were not explicitly acknowledged by the authors of each study, they were inferred by the primary author.

### Risk of bias assessment

To assess the validity of the studies, the primary reviewer (EP) conducted a risk of bias assessment using the Critical Appraisal Skills Program (CASP) checklists.^37^ The CASP RCT and cross‐sectional studies checklists, which include 11 questions for evaluating bias and study quality, were applied to the RCTs, mixed‐methods, and cross‐sectional studies included in the review.

**TABLE 2:** Study characteristics of included studies.

Study (authors, year of publication, country, reference number)	Study design	Participants	Exposure/intervention	Control intervention/comparator	Study aims	Outcomes regarding impact on students' understanding of neuroanatomy
Computer‐assisted learning (CAL) resources						
Lamperti and Sodicoff, 1997^38^ (United States)	Not specified	First year medical students (*N* = 185)	CAL program consisting of a computerized atlas, a laboratory guide and a clinical problem‐solving section	N/A	Development of a CAL resource to replace the traditional glass‐slide/microscope‐based laboratory component of a course and compare student performance between students who received the traditional program versus the CAL program	Students who were trained via the CAL resource scored significantly higher (*p* < 0.0001) than students who were taught by the traditional method
Allen et al. 2008^39^ (United States)	Not specified	First year medical students (*N* = 856)	Treatment 1: Didactic lecture & dissection lab for years 1995–1997 Treatment 2: Didactic lecture & dissection lab & PowerPoint lecture notes on CD video game console for year 2000 Treatment 3: Didactic lecture & dissection lab & web‐based lecture notes, & web‐ based interactive learning objects (patient case studies, review games, stimulated interactive patients, flashcards, and quizzes) for years 2001–2005	N/A	To determine the effect of web‐based interactive instructional techniques on students' written exam performance and students' opinions on elements of the web‐based resource	A statistically significant difference was observed between treatments 1 and 3 but not between treatments 1 and 2 or treatments 2 and 3 Students who received treatment 3 correctly answered a higher number of questions compared to students who received any of the other two types of treatment
Svirko and Mellanby, 2008^40^ (United Kingdom)	Not specified	Second year medical students (*N* = 205)	Neuroanatomy CAL course	N/A	To evaluate how successful a neuroanatomy CAL course was at encouraging students to employ a deep approach to learning	Students reported higher surface approach scores and lower deep approach scores for the CAL course than for their studies in general
Lewis et al. 2011^41^ (Canada)	Randomized controlled study	Second year medical students (*N* = 39)	Web‐based neuroanatomy localization application	Two neuroanatomy textbook resources	To evaluate the educational effectiveness of a web‐based neuroanatomy localization application	Student scores for the MCQ test were 67.5% and 54.7% for the intervention and control group, respectively The intervention group showed a significantly higher (*p* = 0.028) mean raw score than the control group
Brewer et al. 2012^42^ (Canada)	Randomized controlled study	Second year medical students (*N* = 118) & health science students (*N* = 13)	Health science students: Group 2/2D Group: Pre‐digital lab or Group 3/3D Group: 3D pre‐ digital lab Medical students: 3D Pre‐digital lab	Health science students: Control group Medical students: No comparator as they experienced both the digital lab and gross lab but in a different order	To evaluate the effectiveness of a CAL digital lab resource in improving the participants' knowledge of neuroanatomy post‐lecture and either pre or post the gross lab practical	Health science students: No statistically significant differences were found between the three groups neither between the two experimental groups. Post‐test scores of students in Group 3 were the highest from all three groups Medical students: There were no statistically significant differences between the two groups
Ruisoto Palomera et al. 2014^43^ (Spain)	Not specified	Medical students (*N* = 65) *Year of study was not specified	Computer‐based tool	N/A	To develop a computer‐based tool to explore neuroanatomy based on three‐ dimensional images and to compare whether the educational value assigned by students varies according to their visuospatial ability	No statistically significant differences were observed between students who showed high visuospatial ability and students who showed low visuospatial ability in any of the four items of the survey (*p* > 0.01)
Drapkin et al. 2015^44^ (United States)	Randomized controlled study	First year medical students (*N* = 62)	Neuroanatomy lecture with 3D neuroanatomy teaching tool (*N* = 33)	Neuroanatomy lecture with traditional methods (*N* = 29)	To develop a computerized (3D) neuroanatomy teaching tool for training medical students to identify subcortical structures on a magnetic resonance imaging (MRI) series of the human brain and to assess its efficacy	There were no statistically significant differences between the MRI identification scores across the experimental and control groups A statistically significant difference (*p* < 0.01) was observed for questions that involved C‐shaped structures as the experimental group outperformed the control group by an estimated difference in average score of 15.8%
Allen et al. 2016^45^ (Canada)	Randomized crossover study	Second year medical students (*N* = 47)	Group A: Access to the online 3D learning module and subsequently had access to the cadaveric laboratory session Group B: Access to the cadaveric laboratory session and subsequently had access to the online 3D learning resource	N/A	To examine the educational efficacy of a newly developed 3D neuroanatomy module	The only statistically significant difference between Groups A and B was observed for the post‐test scores as it was shown that students who accessed the 3D learning module before accessing the cadaveric laboratory session performed significantly better on this test (*p* < 0.01) Statistical analysis of scores within each group revealed that students in both groups scored significantly higher on the post‐test than on the pre‐test (*p* < 0.01)
Peterson and Mlynarczyk, 2016^46^ (United States)	Not specified	Graduate medical students (*N* = 51) Upper‐level undergraduate medical students (*N* = 5) Total number of students: *N* = 56 *Year of study was not specified	Traditional teaching material augmented with computerized 3D teaching tools	Traditional teaching material	To analyze the examination performance of students on questions covering material taught with traditional learning formats versus material covered with traditional formats augmented with digital 3D technology	Student performance for questions covering material taught with 3D teaching tools was statistically significantly higher (*p* < 0.0001) than for questions covering material taught with traditional teaching methods Students performed significantly better (*p* < 0.0001) on cadaveric SPOT exam questions that were taught with 3D teaching tools than for cadaveric SPOT exam questions taught with traditional teaching methods
Svirko and Mellanby, 2017^47^ (United Kingdom)	Not specified	Second year medical students (*N* = 869)	Neuroanatomy CAL course	Traditional neuroanatomy course	To compare the approach to learning that the students adopted toward a neuroanatomy CAL course with their approach to the more traditional aspects of their neuroanatomy instruction and examine whether students' approach to learning related to their performance in tests and examinations	Students' deep approach scores were significantly lower for the CAL course than for the traditional neuroanatomy course (*p* < 0.001)
Welch et al. 2020^48^ (United States)	Not specified	First year medical students (*N* = 314)	Neuroanatomy multimedia learning module	N/A	To assess the effectiveness of a neuroanatomy multimedia learning module that was developed and was offered as optional study aids to pre‐clinical medical students	No statistically significant differences were observed in the study conducted by Welch et al. (2020)^48^
Javaid et al. 2020^49^ (Ireland)	Single‐blinded controlled study	1st year graduate medical students (*N* = 34), second year undergraduate medical students (*N* = 46) & third‐year undergraduate clinical therapy students (*N* = 5)	Experimental group [*N* = 28]: Interactive CAL resource on spinal pathways developed by Javaid et al. No‐use group [*N* = 36]: No use of the interactive CAL resource developed by Javaid et al., neither use of the functional neuroanatomy resource developed by Krebs et al.	Control group [*N* = 21]: Functional neuroanatomy resource developed by Krebs et al.	To evaluate whether an interactive e‐resource focusing on the spinal pathways assists medical students' learning of neuroanatomy	No statistically significant differences were observed for participants in each of the three groups regarding their percentage of correct answers in the pre‐ or the post‐test in relation to the questions' level of difficulty For all three groups, there was a statistically significant difference in students' neuroanatomy knowledge on the spinal pathways between the pre‐ and post‐tests for the easy and difficult questions and total scores overall Students in all three groups performed statistically significantly better in the post‐test No statistically significant difference was noted across students' median normalized learning gain scores A statistically significant difference was observed for the median normalized learning gains of participants in the no‐use group for the easy versus difficult questions (*p* < 0.001)
Van Walsum Cappellen and Hennssen, 2022^50^ (Netherlands)	Randomized controlled study	Second year medical students (*N* = 38)	Group 1: Students were exposed to E‐Learning modules 1, 2 and 4 (*N* = 19) Group 2: Students were exposed to E‐learning modules 1, 3 and 4 (*N* = 19)	N/A	To evaluate the effect of spatial ability on cross‐sectional e‐learning brainstem anatomy and examine the learning outcomes of students working with PLI images and learning outcomes of students working with LFB images	No statistically significant differences were observed between the MRT scores of male and female medical students (*p* = 0.134) No statistically significant differences were observed between the pre‐intervention, post‐intervention and long‐term test scores of the two groups Post‐intervention and long‐term anatomical test scores were significantly higher than pre‐intervention test scores for both groups indicating improvement irrespective of the e‐learning module students were assigned
Booker et al. 2024^51^ (United Kingdom)	Cross‐sectional study	Second year medical students (*N* = 76)	Online video resources from Soton Brain Hub focusing on ‘pain pathways’ (*N* = 53)	Paper copy of a text‐based resource focusing on ‘pain pathways’ (*N* = 23)	To compare the effectiveness of text‐based resources and online video learning resources from the Soton Brain Hub online educational platform	Students who accessed the Soton Brain Hub video resources showed significantly higher immediate learning gains than students who accessed the text‐based resources (*p* = 0.030) No statistically significant differences were observed for the retained learning gains of the two groups (*p* = 0.919)
Xuan et al. 2024^52^ (China)	Randomized controlled study	Undergraduate medical students (*N* = 50) *Year of study was not specified	GRAVEN database: Access to GRAVEN CAL resource and traditional neuroanatomy teaching methods	Control group: Students received traditional teaching methods (PowerPoint teaching, Rhoton's cranial anatomy and surgical approaches, neurosurgery tricks of the trade) (*N* = 25)	To develop the GRAVEN database to help students learn neuroanatomy better and evaluate whether access to both the database and traditional neuroanatomy teaching methods improves academic performance	Students who had access to traditional neuroanatomy teaching methods and the GRAVEN CAL database scored statistically significantly higher in the test than students who had access to traditional neuroanatomy teaching methods only (*p* = 0.0026)
Yun et al. 2024^53^ (Republic of Korea)	Randomized control study	First year medical students (*N* = 154)	Virtual dissection group (*N* = 71): Accessed the complete anatomy app on a tablet to study structures of the diencephalon, telencephalon and structures associated with the third and lateral ventricles	Donor group (*N* = 83): Accessed donor dissections which were prepared in advance by tutors	To investigate medical students' academic performance and satisfaction when utilizing virtual versus donor dissections	The mean score of students in the virtual group for Quiz 1 was statistically significantly higher than that in the donor group (*p* < 0.05) No statistically significant differences were observed between the mean scores of Quiz 2 between the two groups
Stereoscopic resources						
Köckro et al. 2015^54^ (Germany)	Randomized controlled study	Second year medical students (*N* = 169)	3D group: Pre‐recorded audio lecture on the third ventricle & 3D animated tour of the third ventricle with DextroBeam (*N* = 89)	Control group/2D group: Pre‐recorded audio lecture on the third ventricle & 2D PowerPoint presentation (*N* = 80)	To evaluate the efficacy of a stereoscopically presented 3D neuroanatomical model to a large group of students and assess students' retention of anatomical knowledge	Mean score for the MCQ test was higher for the 3D group (5.45) than the 2D Group (5.19), however, there was no statistically significant differences between the two groups Due to absence of a large sample size (*N* = 169; 3D group: *N* = 89, 2D group: *N* = 80), the superiority of the 3D teaching over the 2D teaching cannot be determined accurately
de Faria et al. 2016^55^ (Brazil)	Randomized controlled study	Graduate medical students (*N* = 84) *Year of study was not specified	Group 1: Received a traditional lecture exhibiting 2D images Group 2: Received a lecture that used interactive non‐stereoscopic methods Group 3: Received a lecture that used interactive stereoscopic methods to demonstrate 1 non‐stereoscopic and 1 stereoscopic video	N/A	To develop and evaluate a virtual stereoscopic resource for neuroanatomy teaching	Students in Groups 2 and 3 scored significantly higher than students in Group 1 in the post‐test scores for their written theory exam (*p* < 0.05) Comparison of the mean values of the pre‐ and post‐tests showed that there was a statistically significant improvement in the post‐test scores of Groups 2 and 3 In terms of scores of the practical exam, there was a statistically significant difference in the mean scores between Group 1 and Groups 2 and 3
Bernard et al. 2020^56^ (France)	Prospective randomized controlled study	Second year medical students (*N* = 175)	3D group: 3D stereoscopic video (*N* = 91)	2D group: Non‐ stereoscopic video (*N* = 84)	To investigate whether a 3D stereoscopic instruction video on the circle of Willis could improve learning over a 2D video	There was a statistically significant difference between the scores of the two groups as students from the 3D group scored higher than students from the 2D group in the anatomical relations and clinical reasoning sections (*p* = 0.01)
Yohannan et al. 2024^57^ (India)	Three‐limb randomized controlled study	First‐year medical students (*N* = 152)	Stereoscopic group: Students received a 20‐min demonstration on the brainstem lesson module via AnaVu in stereoscopic mode Monoscopic group: Students received a 20‐min demonstration on the brainstem lesson module via AnaVu in monoscopic mode	Control group: Students were taught neuroanatomy via white board drawn diagrams	To assess the utility of the AnaVu tool compared to conventional methods	All three groups showed statistically significant improvement from pre‐ to post‐test (*p* < 0.001) The Stereo group scored significantly higher than both the Mono (*p* = 0.03) and control groups (*p* = 0.001) in basic recall questions, and both the Stereo and Mono groups outperformed the control group in radiology‐based questions (*p* < 0.001 and *p* = 0.046, respectively)
Virtual reality (VR) resources						
Stepan et al. 2017^58^ (United States)	Randomized controlled study	First year medical students (*N* = 34) & second year medical students (*N* = 32)	VR group: VR model of brain anatomy; students had a 10‐min VR study comprised of a 5‐min 3D video showing key anatomic relationships & 5 min of a fully immersive VR experience, and a 10‐min session with control study materials	Control group: 20‐min independent study with control study materials *Study control materials were not defined by the authors	To evaluate the effectiveness, satisfaction, and motivation associated with immersive VR simulation in teaching medical students neuroanatomy	No statistically significant differences were observed between the VR and control groups for their scores on the pre‐intervention, post‐intervention, or retention quizzes Second year medical students scored significantly higher than first year medical students in all three quizzes (*p* < 0.01)
Ekstrand et al. 2018^59^ (Canada)	Randomized controlled study	First year medical students (*N* = 41) & second year medical students (*N* = 23)	VR group (*N* = 31): Virtual reality learning material illustrating a set of brain structures Virtual reality brain was presented via a headset using two handheld remotes; participants had 12 min of study time to memorize spatial relationships	Paper‐based group: (*N* = 33) Booklet containing 15 colored figures and corresponding labels for the same set of brain structures presented in the VR group. Colored figures were obtained from the Blumenfeld's neuroanatomy through clinical cases, textbook; participants had 12 min of study time to memorize spatial relationships	To examine the impact of immersive virtual‐reality neuroanatomy training and compare it to traditional paper‐based methods	No statistically significant differences were observed between student scores from the two groups for the pre and two post‐intervention tests
Richard and Rajakumari, 2023^60^ (India)	Randomized controlled study	First‐ and second‐year medical students (*N* = 66) *The exact number of medical students in their first and second year was not specified	Experimental group (*N* = 33): Participants received a five‐minute lesson to become familiar with iPads loaded with the VR application. Subsequently, participants accessed a range of neuroanatomical structures	Control group (*N* = 33): Used traditional paper methods that showed the same neuroanatomical structures present in the VR application	To evaluate the short‐term and long‐term influence of 3D‐VR technology on learning outcomes, when compared to traditional teaching models	Male and female participants in the control group scored statistically significantly higher in their post‐test than in their pre‐test (Males: *p* < 0.001; Females: *p* = 0.01) Male and female participants in the intervention group scored statistically significantly lower in their post‐test than in their pre‐test (Males: *p* < 0.001; Females: *p* = 0.03) Male and female participants in the control and intervention groups scored statistically significantly higher in their second post‐test than in their first post‐test (VR Group: Males: *p* < 0.001, Females: *p* = 0.03; Control group: Males: *p* < 0.001, Females: *p* = 0.03)
Augmented reality (AR) resources						
Küçük et al. 2016^61^ (Turkey)	Mixed‐methods study	Second year medical students (*N* = 70)	Experimental group: Participants were taught by traditional presentation material such as 2D pictures, graphs, and text and had access to the MagicBook mobile Augmented Reality (mAR) App that focused on the ascending and descending pathways; MagicBook consisted of 6 3D video animations, 3D human anatomy model and two diagrams which were supplementary material for the experimental group only Participants used MagicBook to review the teaching material (*N* = 34) [females = 16; males = 18]	Control group: Participants were taught by traditional presentation material such as 2D pictures, graphs, and text Participants used a traditional textbook to review the teaching material	To determine the effects of learning anatomy via mAR on medical students' academic achievement and cognitive load	Students from the experimental group were statistically significantly more successful (*p* < 0.05) than students from the control group and were statistically significantly found to have lower cognitive load compared to students from the control group (*p* < 0.05)
Henssen et al. 2020^62^ (Netherlands)	Randomized controlled study	First year medical students (*N* = 23) & biomedical sciences students (*N* = 8)	AR group Practical assignment 1: Students received an overview of the anatomy of the human brain Practical assignment 2: Students studied subcortical structures using the GreyMapp augmented reality application	Control group Practical assignment 1: Students received an overview of the anatomy of the human brain Practical assignment 2: Students studied subcortical structures using cross‐sections (anatomical drawings of transverse sections of the human brain)	To investigate the differences on test scores, cognitive load, and motivation after neuroanatomy learning using AR applications or using cross‐sections of the brain	No statistically significant differences were observed between the pre and post‐test scores of the two groups Post‐test scores from both groups were significantly higher than their pre‐test scores; post‐test scores for the control group were significantly higher than post‐test scores for the GreyMapp‐AR group Statistical analysis of the three components of the post‐test showed that students from the control group scored significantly better on the third part of the test (cross‐sections) than students from the GreyMapp AR group
Cercenelli et al. 2024^63^ (Italy)	Not specified	Second‐year medical students (*N* = 70)	AR group: Had access to the AEducAR3.0 platform	N/A	To explore the effectiveness of the AEducAR3.0 hybrid platform which combines AR with 3D models in allowing students to study neuroanatomy at different learning levels (notional learning, notional learning in context and topographical learning)	Students achieved a higher correct response rate for the quiz of learning level two when compared to the correct response rate (70% ± 4%) for the quizzes of learning levels 1 and 3 (60% ± 7% and 58% ± 5%, respectively) (Learning level 1 vs. Learning level 2: *p* = 0.04; Learning level 2 vs. Learning level 3: *p* = 0.02)
Zeedzen‐Scheffers et al. 2024^64^ (Netherlands)	Randomized controlled study	First year (*N* = 5) and second year (*N* = 23) medical and biomedical sciences students Total number of participants *N* = 28 *The exact number of medical students and biomedical sciences students in their first and second year was not specified	AR group: Had access to the GreyMapp AR resource to complete their preparatory assignment prior attending their body donor‐based education Preparatory assignment 1: Students received an overview of the anatomy of the human brain	Control group: Had access to Sobotta anatomical atlas resource to complete their preparatory assignment prior attending their body donor‐based education	To investigate the impact of the GreyMapp resource on students' academic achievement when used as a preparatory teaching tool by students prior attending their body donor‐based education session, and the effect of AR versus traditional resources on students' cognition	Both the AR and control groups showed significant improvement from pre‐ to post‐test scores (*p* < 0.001), however, there were no statistically significant differences between the groups in the MRT scores (*p* = 0.09) or in their pre‐ and post‐test score comparisons (*p* = 0.35)
Mixed reality (MR) resource						
Pickering et al. 2022^65^ (United Kingdom)	Quasi‐randomized control trial	Second year medical students (*N* = 200)	MR resource or anatomy drawing screencast	N/A	To explore the impact of a MR resource focusing on the sensory and motor spinal pathways in comparison to a drawing screencasts multimedia video resource which was a pre‐existing resource already embedded in the medical curriculum	The percentage scores of students for the MCQ and SAQ sections and overall score were statistically significantly higher in the post‐test irrespective of the resource the students have been exposed to (MR: *p* < 0.001; Screencast: *p* < 0.001) When absolute and normalizing gains were applied to the data, it was shown that the only statistically significant increase in learning gain was observed in the MCQ section of the post‐test for the screencast group (absolute gain, *p* < 0.003; normalizing gain, *p* < 0.01)
Utilization of a VR and an AR resource						
Gurses et al. 2024^66^ (Turkey)	Not specified	Neurosurgery residents (*N* = 40) & second‐year medical students (*N* = 200)	Neurosurgery residents: Accessed the VR‐based 3D models Medical students: Accessed the AR‐based 3D models	N/A	To investigate the effectiveness of teaching neuroanatomy with AR and VR resources when cadaveric dissection is not available	Neurosurgery residents scored higher than medical students in the pre‐test (7.5/10 vs. 4.8/10) Both neurosurgery residents and medical students scored statistically significantly higher in their post‐test scores (*p* < 0.001 and *p* < 0.001, respectively)

Abbreviations: 2D, two‐dimensional; 3D, three‐dimensional; AR, augmented reality; CAL, computer‐assisted learning; E‐learning, electronic learning; LFB, luxol fast blue; mAR, mobile augmented reality; MCQ, multiple‐choice‐questions; MR, mixed reality; MRT, mental rotation test; N/A, not applicable; PLI, polarized light imaging; VR, virtual reality.

## RESULTS

### Study selection

**FIGURE 1:**
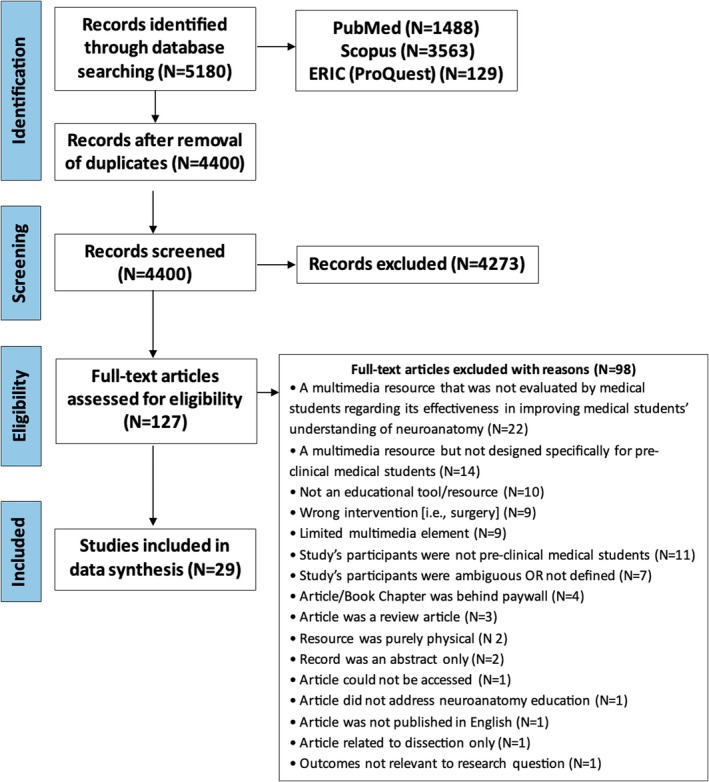
Flowchart of article selection summary according to PRISMA‐P guidelines.^35^

The electronic search across three databases identified a total of 5180 articles. Subsequently, these files were imported into Covidence to remove any duplicates before commencing the screening process. After duplicates were removed, 4400 articles were screened based on their titles and abstracts. Each abstract was reviewed for the inclusion criteria, leaving 127 articles for secondary screening. During the secondary screening, the full texts of the 127 articles were reviewed and 98 articles were excluded as they did not directly address the research question of this systematic review. The reasons for excluding studies during the secondary screening process are detailed in the PRISMA‐P flow chart (Figure 1). Ultimately, 29 articles were selected for final data extraction (Table 2).

### Study characteristics

The 29 studies included in the review were published between 1997 and 2024, with the majority published in 2024 (Table 2). These studies originated from 14 countries: the United States (*n* = 6),^38^, ^39^, ^44^, ^46^, ^48^, ^58^ Canada (*n* = 4),^41^, ^42^, ^45^, ^59^ United Kingdom (*n* = 4),^40^, ^47^, ^51^, ^65^ Netherlands (*n* = 3),^50^, ^62^, ^64^ France (*n* = 1),^56^ Republic of Ireland (*n* = 1),^49^ Italy (*n* = 1),^63^ Republic of Korea (*n* = 1),^53^ China (*n* = 1),^52^ Brazil (*n* = 1),^55^ Spain (*n* = 1),^43^ Germany (*n* = 1),^54^ India (*n* = 2),^57^, ^60^ and Turkey (*n* = 2)^61^, ^66^ (Table 2).

Among the 29 studies, seven studies^40^, ^42^, ^51^, ^55^, ^64^, ^65^, ^66^ focused solely on evaluating the impact multimedia resources have on students' understanding of neuroanatomy, while the remaining 22 studies assessed both students' perceptions (in terms of satisfaction or motivation) and the impact of multimedia resources on their understanding of neuroanatomy.^38^, ^39^, ^41^, ^43^, ^44^, ^45^, ^46^, ^47^, ^48^, ^49^, ^50^, ^52^, ^53^, ^54^, ^56^, ^57^, ^58^, ^59^, ^60^, ^61^, ^62^, ^63^ Of these 29 studies, 18 were randomized control studies,^41^, ^42^, ^44^, ^45^, ^49^, ^50^, ^52^, ^53^, ^54^, ^55^, ^56^, ^57^, ^58^, ^59^, ^60^, ^62^, ^64^, ^65^ one was a mixed‐methods study,^61^ one was a cross‐sectional study^51^ and nine studies did not specify their study design.^38^, ^39^, ^40^, ^43^, ^46^, ^47^, ^48^, ^63^, ^66^ The study characteristics for each article were extracted using the PICO(s) model and are summarized in Table 1. A summary of each study can be found in Table B1 (Appendix B).

### Multimedia technologies used

**FIGURE 2:**
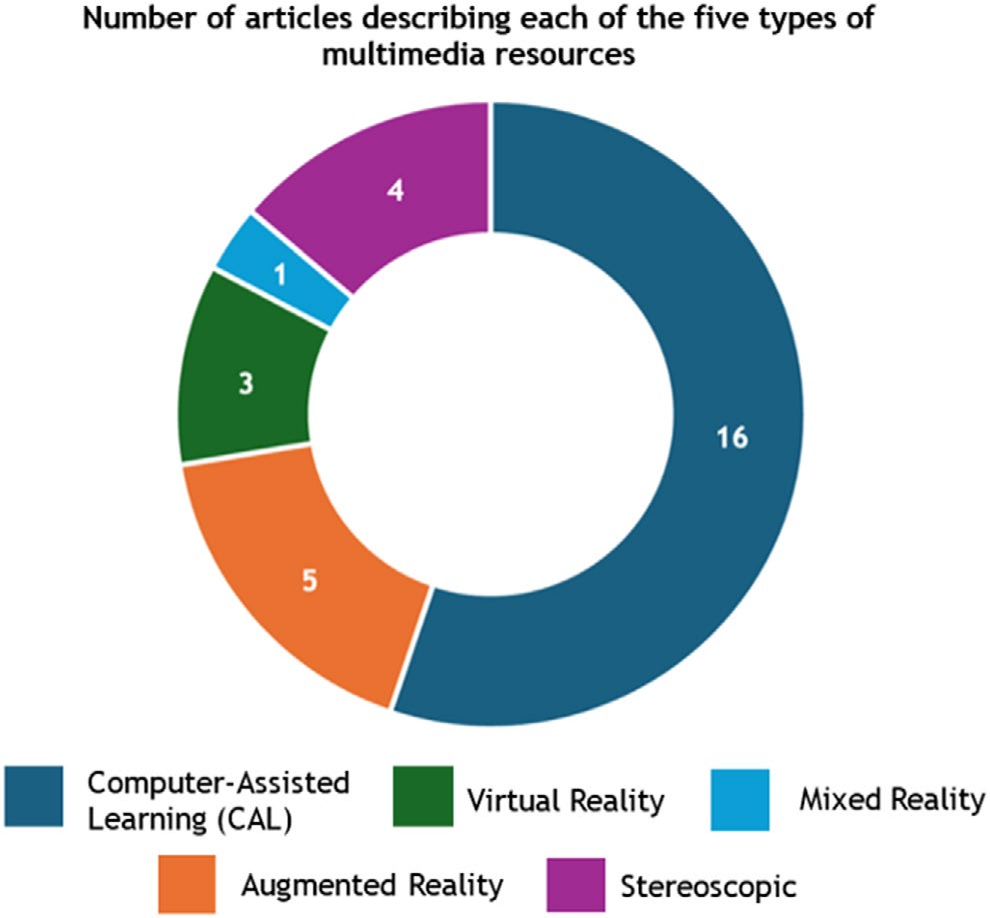
A donut chart indicating the number of articles describing each of the 5 types of multimedia resources that were identified after the 29 articles included in the final analysis were assessed. There was one study that described a resource that fitted into more than one type of a multimedia resource category (Augmented Reality and Virtual Reality), however, the resource that was utilized by medical students was an augmented reality resource, whereas the virtual reality resource was utilized by neurosurgery residents; hence, the augmented reality resource was categorized under the “Augmented Reality” category only.

The 29 studies identified five types of multimedia technology resources: (1) virtual reality (VR), (2) augmented reality (AR), (3) mixed reality (MR), (4) stereoscopy, and (5) computer‐assisted learning (CAL) resources (Figure 2). Of the 29 studies, 16 described CAL resources,^38^, ^39^, ^40^, ^41^, ^42^, ^43^, ^44^, ^45^, ^46^, ^47^, ^48^, ^49^, ^50^, ^51^, ^52^, ^53^ four described stereoscopic resources,^54^, ^55^, ^56^, ^57^ three described VR resources,^58^, ^59^, ^60^ five described AR resources,^61^, ^62^, ^63^, ^64^, ^66^ and one study described an MR resource.^65^

### Study participants

The studies included data from a total of 4607 students. Of these, 1855 were first‐year undergraduate and graduate medical students, 2377 were second‐year undergraduate medical students, and 26 were health‐allied profession students. Additionally, 135 students were classified as graduate medical students, 115 students as medical students, and five as upper‐level undergraduate medical students although their exact year of study was not specified. One study included five first‐year medical and biomedical sciences students and 23 second‐year medical and biomedical sciences students, however, the exact number of medical and biomedical students within each year was not provided. Another study had 66 participants which were first‐ and second‐year medical students, however, the exact number of medical students within each year was not provided. Hence, the corresponding authors and co‐authors of the studies where the participants' year of study was not specified, have been contacted via email or on ResearchGate to ask for clarifications, however, no responses have been received.

### CAL resources

Sixteen studies described the use of computer‐assisted learning (CAL) resources.^38^, ^39^, ^40^, ^41^, ^42^, ^43^, ^44^, ^45^, ^46^, ^47^, ^48^, ^49^, ^50^, ^51^, ^52^, ^53^ In 13 of these studies, the CAL resource was assessed in terms of medical students' perceptions of the resource and its impact on their understanding of neuroanatomy,^38^, ^39^, ^41^, ^43^, ^44^, ^45^, ^46^, ^47^, ^48^, ^49^, ^50^, ^52^, ^53^ while the remaining three studies focused solely on the impact of the CAL resource on students' understanding of neuroanatomy only.^40^, ^42^, ^51^

In eight studies, participants were exposed to a CAL resource without any comparator.^38^, ^39^, ^40^, ^42^, ^43^, ^45^, ^48^, ^50^ In one study,^42^ where second‐year health science students and second‐year undergraduate medical students were involved, a comparator was used only for the health science group. In the remaining eight studies, a comparator was included.^41^, ^44^, ^46^, ^47^, ^49^, ^51^, ^52^, ^53^ These comparators varied from neuroanatomy textbooks,^41^, ^51^, ^52^ traditional neuroanatomy lectures,^44^, ^52^ conventional teaching materials,^46^ traditional neuroanatomy courses,^47^ interactive CAL neuroanatomy resource,^49^ to a donor dissection.^53^ Fourteen out of the 16 CAL resources were designed as adjuncts to support neuroanatomy learning,^39^, ^40^, ^41^, ^42^, ^43^, ^44^, ^45^, ^46^, ^47^, ^48^, ^49^, ^50^, ^51^, ^52^ whereas only one resource aimed to replace traditional teaching materials entirely.^38^ One study utilized a CAL resource as an adjunct with the potential to replace donor dissection later on.^53^

The impact of CAL resources on students' understanding of neuroanatomy was assessed in 16 studies, nevertheless, only four studies^45^, ^49^, ^50^, ^51^ employed pre‐ and post‐tests that allowed for measurable changes in students' understanding. Among these, three studies^45^, ^49^, ^51^ reported statistically significant improvements in students' post‐test scores after exposure to the CAL resource. In the study by Allen et al.,^45^ group A, which used the CAL resource before attending the cadaveric laboratory session, scored significantly higher than group B, who accessed the resource afterward. Furthermore, only group A's final knowledge assessment scores were significantly higher (*p* < 0.01) compared to their first post‐test scores. The study by Javaid et al.^49^ found statistically significant differences in pre‐ and post‐test scores across three groups: an experimental group using an interactive CAL resource on spinal pathways, a control group using a functional neuroanatomy resource, and a no‐use group. The learning gain for the experimental group was significantly higher than the no‐use group (*p* = 0.04). In the study by Booker et al.,^51^ students in the intervention group who used CAL video resources from the Soton Brain Hub online educational platform showed statistically significantly higher average learning gains than the text‐based resource control group (*p* = 0.030). Nevertheless, no statistically significant differences were observed in the retained learning gains between the two groups on the retention MCQ test (*p* = 0.919) that participants took 3 weeks after accessing their assigned resource.

All eight studies^41^, ^44^, ^46^, ^47^, ^49^, ^51^, ^52^, ^53^ that used comparators found statistically significant differences between the CAL resource and the comparator in improving students' understanding of neuroanatomy. In the study by Svirko and Mellanby,^47^ a positive correlation was observed between students' deep engagement with the CAL course and their performance on the formative neuroanatomy assessment (*p* < 0.001; *r* = 0.12). Drapkin et al.^44^ found that the experimental group, which used CAL resources, outperformed the control group in questions involving C‐shaped structures, with an average score difference of 15.8% (*p* < 0.01). In the study by Lewis et al.,^41^ the intervention group achieved significantly higher mean raw scores than the control group (*p* = 0.028). Peterson and Mlynarczyk^46^ observed that student performance on questions related to material taught using 3D teaching tools was significantly better (*p* < 0.0001) compared to material taught using traditional teaching methods. Xuan et al.^52^ found that the experimental group, which used the GRAVEN database that consists of pictures of hand gestures that simulate intracranial arteries and veins and neurosurgical approaches, scored statistically significantly better than the control group who only studied neuroanatomy using traditional neuroanatomy teaching methods (PowerPoint and textbooks) (*p* = 0.0026). Yun et al.^53^ conducted two post‐tests (quizzes 1 and 2) after the donor group and the virtual group accessed donor dissections and the CompleteAnatomy app, respectively. Yun et al.^53^ found that the virtual group which accessed the CompleteAnatomy app on a tablet scored statistically significantly higher in Quiz 1 than the donor group (*p* < 0.05). Despite this, the authors reported no statistically significant differences between the two groups for Quiz 2.

### Stereoscopic resources

Four studies described the use of stereoscopic resources.^54^, ^55^, ^56^, ^57^ Three of these studies evaluated both students' perceptions of the stereoscopic resource and its impact on their understanding of neuroanatomy,^54^, ^56^, ^57^ while the fourth study^55^ focused solely on the impact of the stereoscopic resource on students' understanding only. In three of the studies, the stereoscopic resource was compared to a non‐stereoscopic resource,^54^, ^56^, ^57^ while the fourth study did not include a comparator.^55^ Pre‐ and post‐tests were used in three studies,^55^, ^56^, ^57^ whereas the remaining study only administered a post‐test after students were exposed to the stereoscopic resource.^54^

In the study by Bernard et al.,^56^ students who used a stereoscopic resource demonstrating the circle of Willis scored statistically significantly higher (*p* = 0.01) on tests of anatomical relations and clinical reasoning compared to those using a 2D resource. Similarly, in the study by de Faria et al.,^55^ students in the experimental groups—one using an interactive non‐stereoscopic lecture and the other using an interactive stereoscopic lecture, both on the limbic system—scored statistically significantly higher (*p* < 0.05) than students in the control group, who received a traditional 2D lecture on the limbic system. Both experimental groups showed significant improvement in post‐test scores compared to pre‐test scores, and there was a significant difference in practical exam scores between the control group and the experimental groups. However, no statistically significant advantage was found for the stereoscopic method over the non‐stereoscopic one (*p* > 0.05). In the study by Yohannan et al.,^57^ students in the stereoscopic group who received a 20‐min demonstration on the brainstem via the AnaVu stereoscopic resource scored statistically significantly higher in the basic recall questions than students in the monoscopic group (*p* = 0.03) and control group (*p* = 0.001) who received the same demonstration in monoscopic mode and white‐board drawn diagrams, respectively. Also, students in the stereoscopic group scored statistically significantly higher in the radiological questions than students in the control group (*p* < 0.001). In the study by Kockro et al.,^54^ where students completed a post‐test after using either a 2D non‐stereoscopic or a 3D stereoscopic resource focused on the anatomy of the third ventricle, no significant differences were found between the post‐test scores of the stereoscopic and non‐stereoscopic groups.

### Virtual reality (VR) resources

The impact of VR resources on medical students' understanding of neuroanatomy was evaluated in three studies using pre‐ and post‐tests.^58^, ^59^, ^60^ Two studies found no statistically significant differences between pre‐ and post‐test scores.^58^, ^59^ In the randomized controlled study by Stepan et al.,^58^ first‐ and second‐year medical students were randomly assigned to either a VR experimental group or a control group, which used unspecified study materials to learn about the ventricles and vasculature of the brain. No significant differences were observed between the VR and control groups in their pre‐intervention or retention quiz scores. However, second‐year medical students scored significantly higher than first year medical students in all three quizzes. Similarly, in the study by Ekstrand et al.,^59^ first‐ and second‐year medical students were randomly assigned to either a VR experimental group or a control group using paper‐based materials. The resources covered the basal ganglia, adjacent neuroanatomical structures, and the lateral corticospinal and spinothalamic tracts. No significant differences were found between the experimental and control groups in their pre‐test and two post‐intervention test scores. In the third study that evaluated the impact of a VR resource on students' understanding of neuroanatomy, a pre‐intervention test and two post‐intervention tests were employed.^60^ The authors reported that male and female participants in the control group who used paper‐based methods to study the neuroanatomical structures of interest showed a statistically significant improvement from their pre‐ to post‐test scores, while male and female participants in the intervention group who used a VR resource scored significantly lower in their post‐test compared to their pre‐test scores. However, participants in both groups demonstrated a statistically significant improvement in their second post‐test to their first post‐test. Despite this, the authors did not conduct statistical comparisons between the two groups, nor did they analyze the results collectively for male and female participants within each group.

### Augmented reality (AR) resources

The impact of augmented reality (AR) resources on students' understanding of neuroanatomy was assessed in five studies,^61^, ^62^, ^63^, ^64^, ^66^ four of which employed pre‐ and post‐tests.^61^, ^62^, ^64^, ^66^ In two out of these four studies, no statistically significant differences were found between the pre‐ and post‐test scores of the experimental and control groups.^61^, ^62^ One study did not report whether there were any statistically significant differences in the pre‐test scores of the experimental and control groups.^64^ In the remaining study,^66^ a statistically significant difference was observed in the pre‐test scores of the two intervention groups.

In 2016, Kücük et al.^61^ evaluated the effects of learning neuroanatomy using a mobile AR app on second‐year medical students' academic performance and cognitive load. Students were assigned to either the experimental group, which used the mAR app to review material on the ascending and descending pathways, or the control group, which used a traditional neuroanatomy textbook. Univariate ANOVA statistical analysis revealed that the experimental group scored significantly higher (*p* < 0.05) than the control group on the academic achievement test. Additionally, students in the experimental group reported significantly lower cognitive load scores compared to those in the control group. Henssen et al.^62^ examined differences in test scores, cognitive load, and motivation among first‐year medical and biomedical sciences students. Participants were assigned to either the experimental group, which used the GreyMapp‐AR app to study subcortical structures, or the control group, which used cross‐section anatomy drawings. While both groups showed significant improvement in their post‐test scores compared to their pre‐test scores, no statistically significant differences were observed between the two groups. Interestingly, the control group outperformed the AR group on the post‐test (*p* < 0.01). Further analysis revealed that the control group scored significantly better on the third part of the test, which focused on cross‐sections. Both groups experienced higher cognitive load scores (*p* = 0.039) after completing the practical assignments. Overall, post‐test scores improved for both groups, regardless of the resource used. A similar study conducted by Zeedzen‐Scheffers et al.^64^ examined the effectiveness of using the GreyMapp AR resource versus an anatomical atlas in preparing 28 first‐ and second‐year medical and biomedical sciences students for their neuroanatomy prosection‐based practicals. The intervention group had access to the GreyMapp AR resource to complete their preparatory assignment prior to attending their body donor‐based education. Students received an overview of the anatomy of the human brain for their preparatory assignment. The control group had access to an anatomical atlas resource to complete their preparatory assignment prior to attending their body donor‐based education. No statistically significant differences were observed in the pre‐ and post‐test scores of the two groups (*p* = 0.35). In the study by Cercenelli et al.,^63^ the effectiveness of the AEducAR3.0 hybrid platform, which combines AR with 3D printed models, was investigated among 70 second‐year medical students. The AEducAR3.0 platform aims to allow students to study neuroanatomy at three different learning levels (notional learning, notional learning in context, and topographical learning). In this study, there was no comparator present, and participants took a self‐assessment quiz at the end of each learning level. When considering all three learning levels, 51% of students scored above the sufficient threshold (9.6/16) with a median score of 10, and while the quiz difficulty was overall well‐balanced across all three learning levels, students performed significantly better in the quiz of learning level 2 compared to learning level 1 (*p* = 0.04) and 3 (*p* = 0.02). A single study conducted by Gurses et al.^66^ investigated the effectiveness of teaching neuroanatomy using AR and VR resources when cadaveric dissection was not available as a neuroanatomy teaching method. This study did not use a comparator; however, the study participants who were neurosurgery residents (*N* = 40) and second‐year medical students (*N* = 200) accessed VR‐based 3D models and AR‐based 3D models, respectively. Participants took a pre‐ and post‐test. Neurosurgery residents scored higher than medical students in the pre‐test (7.5/10 vs. 4.8/10). Both neurosurgery residents and medical students scored statistically significantly higher in their post‐test scores (*p* < 0.001).

### Mixed reality (MR) resources

Pickering et al.^65^ explored the impact of a mixed reality (MR) resource on medical students' understanding of neuroanatomy, focusing on the corticospinal tract, spinothalamic tract, dorsal column tract, and trigeminothalamic tract. This was compared to a screencast‐based multimedia video resource. Students exposed to either the MR resource or the screencast showed significant improvement in their post‐test scores across multiple sections, including multiple‐choice questions (MCQs), short answer questions (SAQs), and overall scores (MR: *p* < 0.001; screencast: *p* < 0.001). However, no significant differences were observed in the pre‐test scores between the two groups. Although both resources led to improved knowledge retention, the anatomy drawing screencast was found to have a greater effect on students' retention of information. Nevertheless, when learning gains were measured using both absolute and normalized scores, only the MCQ section of the post‐test showed a statistically significant improvement for the screencast group (absolute gain: *p* = 0.003; normalizing gain: *p* = 0.001).

### Multimedia elements across resources described in the included studies

**FIGURE 3:**
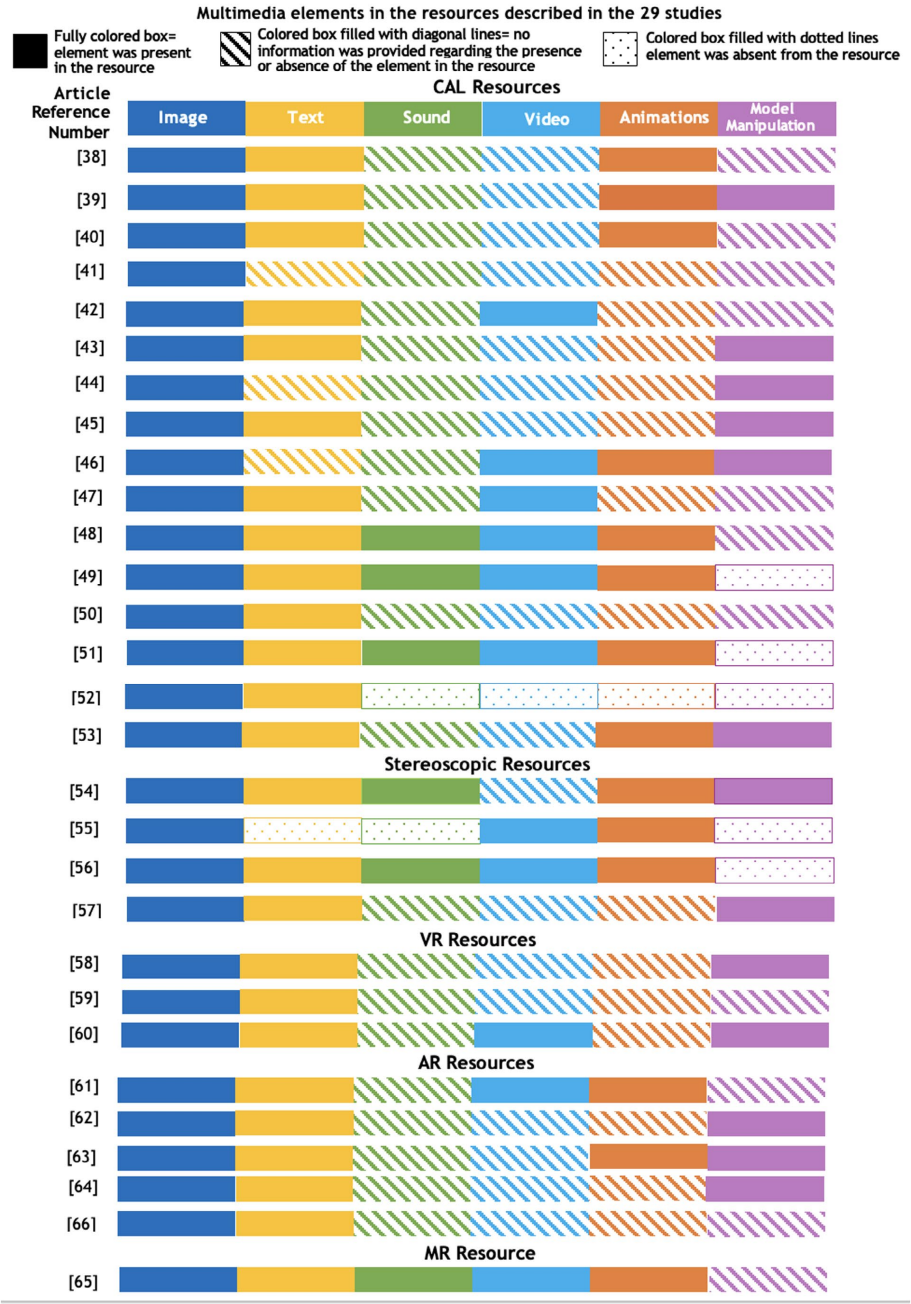
Colored bar charts illustrating the multimedia elements present in the 16 Computer‐Assisted Learning (CAL), four stereoscopic, three virtual reality (VR), five augmented reality (AR), and mixed reality (MR) resources.

The ‘image’ multimedia element was present in all 16 CAL resources,^38^, ^39^, ^40^, ^41^, ^42^, ^43^, ^44^, ^45^, ^46^, ^47^, ^48^, ^49^, ^50^, ^51^, ^52^, ^53^ all three VR resources,^58^, ^59^, ^60^ all five AR resources,^61^, ^62^, ^63^, ^64^, ^66^ all four stereoscopic resources,^54^, ^55^, ^56^, ^57^ and single MR resource.^65^ The “text” element appeared in 13 out of 16 CAL resources,^38^, ^39^, ^40^, ^42^, ^43^, ^45^, ^47^, ^48^, ^49^, ^50^, ^51^, ^52^, ^53^ three of the four stereoscopic resources,^54^, ^56^, ^57^ all three VR resources,^58^, ^59^, ^60^ all five AR resources,^61^, ^62^, ^63^, ^64^, ^66^ and the single MR resource.^65^ The “sound” element was present in three CAL resources,^48^, ^49^, ^51^ in two stereoscopic resources,^54^, ^56^ and the single MR resource.^65^ However, neither the AR resources^61^, ^62^, ^63^, ^64^, ^66^ nor the VR resources^58^, ^59^, ^60^ contained the “sound” element. The “video” element was present in six CAL resources,^42^, ^46^, ^47^, ^48^, ^49^, ^51^ two stereoscopic resources,^55^, ^56^ one AR resource,^61^ one VR resource,^60^ and the MR resource.^65^ The “animations” element appeared in eight CAL resources,^38^, ^39^, ^40^, ^46^, ^48^, ^49^, ^51^, ^53^ in three stereoscopic resources,^54^, ^55^, ^56^ two AR resources,^61^, ^63^ and the MR resource.^65^ None of the VR resources^58^, ^59^, ^60^ included the “animations” element. The “model manipulation” element was found in six CAL resources,^39^, ^43^, ^44^, ^45^, ^46^, ^53^ in two stereoscopic resources^54^, ^57^ in two VR resources,^58^, ^60^ and in three AR resources.^62^, ^63^, ^64^ The MR resource^65^ lacked the “model manipulation” element. A detailed representation of the multimedia elements present or absent in each resource can be seen in Figure 3.

### Effectiveness of neuroanatomy multimedia resources on medical students' understanding of neuroanatomy

Out of the 29 articles reviewed to answer the research question, 15 studies administered a pre‐ and post‐intervention test to assess the effectiveness of multimedia resources on students' understanding of neuroanatomy.^45^, ^49^, ^50^, ^51^, ^55^, ^56^, ^57^, ^58^, ^59^, ^60^, ^61^, ^62^, ^64^, ^65^, ^66^ Among these 15 studies, only seven showed a statistically significant improvement in students' understanding after exposure to the multimedia resource.^45^, ^49^, ^55^, ^56^, ^57^, ^64^, ^66^ Three of these studies focused on stereoscopic resources,^55^, ^56^, ^57^ one study focused on an AR resource,^64^ two studies focused on CAL resources,^45^, ^49^ and one study focused on an AR and a VR resource.^66^

### Risk of bias within studies

Of the 29 studies included in the final analysis, 20 were eligible for risk of bias assessment using the CASP checklist, which included the 18 randomized controlled studies, a single mixed‐methods study, and a single cross‐sectional study. A complete risk assessment could not be performed due to insufficient information, with many answers marked as “No” or “Cannot tell”. Despite this, individual types of bias—such as selection, performance, detection, and reporting bias—were assessed (Articles 1–19, Table C1, Appendix C, Article 20, Table C2, Appendix C).

Out of the 20 eligible studies, only one study exhibited selection bias as its participants were neurosurgery residents and medical students, with each group accessing a different type of an immersive multimedia resource.^66^ A high risk of performance bias was identified in one study.^42^ Seven studies^41^, ^42^, ^55^, ^60^, ^61^, ^62^, ^66^ showed a relatively high risk of reporting bias. For detection bias, it was unclear in all 20 studies whether participants, investigators, and assessors were blinded to the intervention and outcomes, as the responses to question 4 and its sub‐questions (4a, b, c) were predominantly “No” or “Cannot tell”. A summary of the risk of bias assessment for the eligible studies can be found in the supplementary material (Tables C1 and C2, Appendix C).

### Common themes across resources and the articles describing them

Thematic analysis of the included articles revealed both strengths and weaknesses in the resources and the articles that described them, which are outlined in Tables 3 and 4, respectively.

**TABLE 3:** Summarizes four strengths that were identified in some of the resources described in the 29 articles that were included in the final analysis.

Strengths identified in some of the resources described in the included studies	Type of multimedia resource					Total number of articles where resource exhibited this strength
	CAL (*N* = 16)	Stereoscopic (*N* = 4)	VR (*N* = 3)	AR (*N* = 5)	MR (*N* = 1)	
Resource exposed students to radiological imaging scans (i.e., MRI and CT scans)	[41, 42, 43, 44, 46]	[57]	[60]	[64]	N/A	8
Resource contained interactive features such as quizzes, feedback, and diagrammatic trees	[38, 39, 40, 47, 48, 49, 50, 53]	N/A	N/A	N/A	N/A	8
Resource contained a clinical application element	[38, 39, 40, 41, 47, 49, 51, 52]	N/A	N/A	[63]	[65]	10
Resource was designed based on evidence‐based principles (i.e., cognitive load theory and principles of multimedia learning)	[39, 45, 49, 50, 51]	[57]	N/A	[61, 62, 64]	[65]	10

Abbreviations: AR, augmented reality; CAL, computer‐assisted learning; CT, computed tomography; MR, mixed reality; MRI, magnetic resonance imaging; VR, virtual reality.

**TABLE 4:** Summarizes 12 existing weaknesses present across some of the resources described in the 29 articles that were included in the final analysis.

Weaknesses identified in some of the resources or the articles describing them	Type of multimedia resource					Total number of articles where resource or article exhibited this weakness
	CAL (*N* = 16)	Stereoscopic (*N* = 4)	VR (*N* = 3)	AR (*N* = 5)	MR (*N* = 1)	
Resource was not accessible	[38, 39, 40, 42, 43, 44, 45, 46, 47, 50, 53]	[54, 55, 57]	[58, 59, 60]	[61, 62, 64, 66]	[65]	22
Link provided to access resource was dysfunctional	[41]	N/A	N/A	N/A	N/A	1
Information regarding the description of resource was extremely limited	[40, 41, 42, 43, 45]	[55]	[60]	N/A	[65]	8
Article provided extremely limited information on how user interacts while using the resource or how user is expected to use the resource	[40, 41, 42, 43, 50]	[55]	[60]	[61]	[65]	9
Article does not provide adequate information on how resource was created to allow its recreation by others	[38, 39, 40, 42, 45, 48, 50]	N/A	[58, 60]	N/A	[65]	10
No information was provided on the anatomical structures present in the resource	[40]	[55]	N/A	N/A	N/A	2
Inadequate information regarding the anatomical structures present in the resource	[41, 43, 45, 46, 50, 51]	N/A	N/A	N/A	N/A	6
Article did not provide adequate supplementary material (i.e., figures illustrating screenshots from a resource)	[40, 42, 45, 46]	N/A	[58, 59, 60]	N/A	[65]	8
Inadequate information on methods section (i.e., design and structure of pre‐test/post‐test, amount of time students were given to complete such tests or questionnaires or surveys)	[38, 40, 42, 45, 47, 48, 50, 51, 52]	[55]	[58, 60]	[61, 62]	N/A	14
Inconsistencies present in the results section	[42, 45, 47, 48]	N/A	[60]	N/A	N/A	5
Limited use of multimedia elements (≤three out of the six multimedia elements)	[38, 40, 41, 42, 43, 44, 45, 47, 50, 52]	[55, 57]	[58, 59]	[62, 64, 66]	N/A	17
Resource focuses purely on structural neuroanatomy	[40, 42, 43, 44, 45, 50, 52, 53]	[54, 55, 56, 57]	[58, 59, 60]	[61, 64, 66]	[65]	19

Abbreviations: AR, augmented reality; CAL, computer‐assisted learning; MR, mixed reality; VR, virtual reality.

Regarding the strengths of some resources, four key advantages were identified: (i) exposure to radiological imaging scans (e.g., CT and MRI), (ii) inclusion of interactive features (e.g., quizzes, feedback, and diagrammatic trees), (iii) incorporation of clinical application elements, and (iv) design based on evidence‐based principles (Table 3). However, only eight out of the 29 resources exposed students to radiological imaging scans,^41^, ^42^, ^43^, ^44^, ^46^, ^57^, ^60^, ^64^ eight included interactive features,^38^, ^39^, ^40^, ^47^, ^48^, ^49^, ^50^, ^53^ 10 featured a clinical application element,^38^, ^39^, ^40^, ^41^, ^47^, ^49^, ^51^, ^52^, ^63^, ^65^ and 10 were designed based on evidence‐based principles.^39^, ^45^, ^49^, ^50^, ^51^, ^57^, ^61^, ^62^, ^64^, ^65^

In terms of weaknesses in the resources or the articles, 12 areas of concern were identified (Table 4). The four most common weaknesses were: (i) 22 of the 29 resources could not be accessed,^38^, ^39^, ^40^, ^42^, ^43^, ^44^, ^45^, ^46^, ^47^, ^50^, ^53^, ^54^, ^55^, ^57^, ^58^, ^59^, ^60^, ^61^, ^62^, ^64^, ^65^, ^66^ (ii) 19 articles focused solely on structural neuroanatomy,^40^, ^42^, ^43^, ^44^, ^45^, ^50^, ^52^, ^53^, ^54^, ^55^, ^56^, ^57^, ^58^, ^59^, ^60^, ^61^, ^64^, ^65^, ^66^ (iii) 17 resources contained fewer than three multimedia elements,^38^, ^40^, ^41^, ^42^, ^43^, ^44^, ^45^, ^47^, ^50^, ^52^, ^55^, ^57^, ^58^, ^59^, ^62^, ^64^, ^66^ and (iv) 14 articles lacked sufficient information in the methods section, such as details on the design and structure of the pre‐test/post‐test assessments, the time allocated for completing tests, questionnaires, or surveys.^38^, ^40^, ^42^, ^45^, ^47^, ^48^, ^50^, ^51^, ^52^, ^55^, ^58^, ^60^, ^61^, ^62^

## DISCUSSION

This systematic review aimed to answer the research question of whether neuroanatomy multimedia resources designed for pre‐clinical medical students are effective in improving their understanding of structural and functional neuroanatomy. Unlike previous reviews, this study highlighted a key limitation across the currently documented multimedia neuroanatomy resources: most of these resources primarily focus on structural neuroanatomy, with limited attention given to functional and clinical neuroanatomy.

### Effectiveness of neuroanatomy multimedia resources on students' understanding

Out of the 29 articles included in this review, only seven studies^45^, ^49^, ^55^, ^56^, ^57^, ^64^, ^66^ demonstrated a statistically significant improvement in students' understanding of neuroanatomy after exposure to multimedia resources. Three of these studies evaluated a stereoscopic resource,^55^, ^56^, ^57^ two evaluated a CAL resource,^45^, ^49^ one evaluated an AR resource,^64^ and one evaluated a VR and an AR resource.^66^

In the study by Allen et al.,^45^ the CAL resource focused solely on structural neuroanatomy. In contrast, the study by Javaid et al. focused on structural, functional, and clinical neuroanatomy. A systematic review published in 2009 assessed whether computer‐aided learning facilitates anatomy education.^67^ This review analyzed eight quantitative studies, all of which showed positive results favoring the use of computer‐aided‐learning resources. However, only one study specifically focused on a neuroanatomy resource, which was found to have potential as an adjunct tool for improving students' knowledge.^67^ The review concluded that computer‐aided learning can enhance anatomy education when well‐designed and integrated into the medical curriculum.^67^

Four studies evaluated the effectiveness of stereoscopic 3D resources in enhancing medical students' understanding of neuroanatomy.^54^, ^55^, ^56^, ^57^ While three studies^55^, ^56^, ^57^ found statistically significant improvements in students' scores, the literature suggests that stereoscopic resources are particularly effective in improving spatial visualization of neuroanatomical structures and clinical reasoning.^54^, ^56^, ^68^ The results from Yohannan et al.^57^ complement the literature, as the authors reported that the AnaVu stereoscopic tool helped students improve their visuospatial skills. Moreover, the authors reported that the stereoscopic group yielded a greater effect size regarding the tool's ability to teach radiological anatomy due to the nature of stereopsis integrating spatial concepts better.^57^ However, Bogomolova et al.^69^ emphasized that future research should consider students' visuospatial abilities when assessing the effectiveness of stereoscopic tools. The three studies that showed positive results^55^, ^56^, ^57^ did not account for the potential disadvantage faced by students with lower visuospatial abilities, which may have impacted the outcomes. Interestingly, none of these three studies employed specific psychometric tests such as mental rotation tests to assess students' spatial reasoning.

In the study that evaluated the use of an AR and a VR resource there were two experimental groups.^66^ Neurosurgery residents accessed a VR resource, and medical students accessed an AR resource. The authors reported that neurosurgery residents had statistically significantly higher pre‐test scores than medical students. A potential justification as to why neurosurgery residents had a higher baseline knowledge than medical students might be their extensive substantial clinical experience and knowledge. In this study^66^ even though the two groups accessed a different type of a multimedia resource, both groups scored statistically significantly higher in their post‐test than in their pre‐test. Despite this, the authors do not provide any information on whether a comparison was made between the post‐test scores of the two groups. In the study by Zeedzen‐Scheffers et al.,^64^ the AR group accessed the GreyMapp resource prior attending their body donor‐based education whereas the control group accessed the Sobotta anatomical atlas. The study showed that both groups showed a statistically significant improvement from pre‐ to post‐test scores nevertheless, when the MRT and pre‐ and post‐test scores of the two groups were compared, no statistically significant differences were observed.^64^ In September 2024, Salimi and colleagues^70^ conducted a systematic review and a meta‐analysis of 24 randomized controlled trials to assess the effectiveness of AR and VR in anatomy education. The systematic review and meta‐analysis showed that VR improved knowledge scores to a moderate extent, while AR did not show a significant effect. Moreover, VR was rated as more “useful” compared to other methods nevertheless, the high heterogeneity among studies suggests that further research is needed to clarify the factors that influence these technologies.^70^

### Multimedia elements in neuroanatomy resources

The multimedia elements present in neuroanatomy resources varied across the studies, with the “image” element being the only one consistently included in all 29 resources. The “text” element appeared in 25 resources, while the “video” and “model manipulation” elements were found in 11 and 13 resources, respectively. The “animation” element was present in 14 resources, and the “sound” element was included in just six. Interestingly, neither the augmented reality (AR) nor the virtual reality (VR) resources featured the sound element.

Auditory formats in multimedia resources can range from narration to music and sound effects.^71^ Narration, in particular, supports verbal learning, where students absorb information through text displayed on the screen, such as bullet points.^72^ Including narration in neuroanatomy resources can be especially beneficial, as it allows the educator to guide students through various neuroanatomical structures. This is crucial for students with limited or no prior knowledge of neuroanatomy, as they may struggle to identify and orient structures in brain cross‐sections. Additionally, the use of narration can be useful in improving students' understanding of the function of neuroanatomical structures. For example, the computer‐aided learning (CAL) resource created by Javaid et al.^49^ focused on spinal pathways and incorporated multimedia elements like text, images, and animations. These elements enabled students to visualize and trace sensory neurons from the spinal cord to the medulla oblongata, the thalamus, and finally the primary somatosensory cortex. In specific sections, narrated videos helped students understand the concepts behind each neuronal pathway. The narrator used schematic illustrations of the brainstem at various levels, highlighting anatomical structures (e.g., the internal capsule) and explaining their relationships to other regions (e.g., the lentiform nucleus). The narrator also pointed to structures on the screen, sometimes highlighting them in different colors for emphasis.

Dynamic multimedia elements, such as animations and videos, are particularly useful for explaining functional neuroanatomy.^72^ These concepts are difficult to study through cadaveric dissection or prosected specimens, but animations can simulate processes like sensory stimuli (e.g., pain or temperature), motor actions (e.g., leg flexion), and the flow of information along neuronal tracts. However, while incorporating multiple multimedia elements can enhance learning, it is important to be cautious. Overloading a resource with too many elements at once can overwhelm the learner and lead to cognitive overload.^73^, ^74^ Educators should use multimedia features thoughtfully and sparingly to maximize their effectiveness.

### Interpretation of the disparity between the identified strengths and weaknesses in the thematic analysis

A notable disparity between the number of strengths and weaknesses identified in the included studies or the described resources was highlighted by this systematic review. Only four strengths were identified within the described resources whereas 12 weaknesses were identified within the resources or the studies describing them. This disparity perhaps reflects a combination of challenges associated with conducting high‐quality pedagogical research, assessing educational studies, and the lack of established guidelines for designing and evaluating multimedia resources for neuroanatomy education.

Remarkably, 22 out of the 29 resources were not accessible. It is possible that many of the neuroanatomy multimedia resources evaluated in this systematic review could have been created to address immediate needs, without sufficient attention being given to their usability, pedagogical soundness, or long‐term sustainability. Also, some studies described the effectiveness of CAL resources (e.g., CompleteAnatomy and Anatomage) that were accessed by students once they were purchased by their institution. Hence, when resources come at a financial cost, they are less likely to be open access. On the other hand, immersive technologies such as VR and AR cannot be open access as they require specific equipment to operate; therefore, limiting the number of users that can benefit from their use. Despite this, studies which describe such resources can provide readers with enough context on their creation, the neuroanatomical structures present in them, and how the user interacts with them. This systematic review showed that various studies failed to adequately describe the neuroanatomy multimedia resource (*N* = 8), how it was created (*N* = 10) and the neuroanatomical structures it included (*N* = 8), meaning that recreation of the resource by other neuroanatomy educators might not be feasible. These weaknesses identified in the included studies can be potentially attributed to differences in publishing standards. It is possible that earlier publications were subject to less stringent peer review, which can potentially justify the limited information that was provided regarding a resource's design, usability, and content.

Other weaknesses identified across the included studies by this review included the absence of substantial information on the study's methodology and result sections. Out of the 29 studies, five studies had inconsistencies in their results section and 14 studies did not describe adequately their methodology in terms of the design, structure, and content of the pre‐test and/or post‐test(s) they administered to students. An inference that can be made after the assessment of the 29 studies included for final analysis is that in seven studies, the information on the statistical analysis was problematic. Information on the type of tests that were used to determine statistical significance and correlation along with the corresponding *p* and *r* values and test scores and standard deviation values on either the pre‐test or post‐tests were omitted. Hence, the presence of such limitations within these articles limited the statistical evidence that this review could have provided. Such omissions along with other omissions such as flawed introduction, results, and discussion sections, are common across manuscripts that are being submitted in medical science and medical education journals.^75^, ^76^ Other important information that was often omitted in some studies included the method that was used to carry out randomization of the study participants, whether the allocation sequence was concealed from the participants and the study's investigators, whether the study participants were blind to the study hypothesis and the intervention they were given, or whether the individual analyzing the study's outcomes was blinded. When the aforementioned information was not provided in a study, the risk of bias assessment of this study could not be fully performed; therefore, hindering the determination of the actual risk of bias present in a study. A solution to overcome these challenges would be for peer reviewers and journal editors to request such information if omitted from submitted manuscripts and to provide authors with guidelines on how to write each section of a manuscript, including how to comprehensively report their results. Recently, the Anatomical Sciences Education journal has published two discursive articles with journal‐specific recommended guidelines for survey‐based research,^77^ systematic reviews, and meta‐analyses.^78^ These guidelines can be used by educators during the design process of a study and not solely during the writing process of a manuscript. Familiarity with such guidelines can help tackle poor study design planning, ensure greater methodological rigor, and reproducibility and validity of the evidence reported. Moreover, these guidelines can be used by peer reviewers when reviewing manuscripts to ensure their critical appraisal, reduce subjective bias, and help advance the quality of educational research.

This systematic review has identified four strengths within some of the resources described in the included studies, which were a neuroanatomy multimedia resource exposing students to radiological imaging scans, containing a clinical application element, interactive features, and being designed upon evidence‐based principles. The use of radiological imaging scans and the presence of a clinical application element within a resource are a means of achieving vertical integration in neuroanatomy education, which is one strategy that could help tackle neurophobia.^23^ In vertical integration, pre‐clinical knowledge is designed to be applied to a clinical context or vice versa.^23^ In terms of vertical integration within the medical curriculum, studies showed that when pre‐clinical knowledge was applied to a clinical context by integrating case‐based learning, case‐stimulated interactive lectures, clinical seminars, as well as exposing students to patients, students expressed that their understanding was improved and their motivation toward neuroscience‐related topics was enhanced.^24^, ^79^ Furthermore, a study showed that vertical integration in neuroanatomy teaching resulted in students feeling more prepared once they entered the clinical years of their degree.^24^ Thus, a vertically integrated medical curriculum could assist students in applying their structural and functional neuroanatomy knowledge in the context of clinical neuroanatomy.

### Neuroanatomy resources designed upon evidence‐based principles

The design of a resource upon evidence‐based principles and the incorporation of interactive features within it are means of enhancing learning outcomes and the students' learning experience. Interestingly, only 10 out of the 29 studies included in the final analysis reported that the design of the resource they described was informed by evidence‐based principles.^39^, ^45^, ^49^, ^50^, ^51^, ^57^, ^61^, ^62^, ^64^, ^65^ The design of these 10 neuroanatomy multimedia resources was informed by the principles of multimedia learning^39^, ^45^, ^49^, ^50^, ^65^ and the cognitive load theory.^51^, ^57^, ^61^, ^62^, ^64^ Emphasis on how people learn is essential in medical education.^72^ Understanding how learning takes place can assist in designing effective instruction. Applying the 12 principles of multimedia learning during the design and creation of a multimedia resource could create an effective multimedia learning experience for the students.^72^

Only four out of the five studies that applied multimedia learning principles during the design of the resource specified the principles that were used.^45^, ^49^, ^61^, ^65^ Allen et al.^45^ specified that the design of their CAL resource was guided by the dual channel assumption and coherence effect principles. According to Paivio and Baddeley, visual and auditory information are processed by separate channels in humans.^80^, ^81^ The dual channel assumption is defined as humans possessing a visual channel that processes material that is represented visually or spatially, and an auditory channel that processes material that is auditorily or verbally presented.^72^ The dual channel assumption underlines the cognitive theory of multimedia learning, which is defined as the human brain's limited capacity for processing information in the visual and auditory channels.^82^ Selectively choosing the words and pictures that will be used during instruction is key as the incorporation of a multimedia element that does not directly relate to the material can negatively impact students' learning.^72^

The coherence principle aims to reduce extraneous material that, when present, may hinder students' learning. The coherence principle was used by Pickering et al.^65^ Other principles that were used to reduce extraneous processing are the signaling and temporal and spatial contiguity principles, which were used in three studies.^49^, ^61^, ^65^ The signaling principle underlines that highlighting essential material via the use of headings, pointer words, or the use of an outline in a multimedia lesson can help people learn better.^82^, ^83^ The spatial and temporal contiguity principles underscore that students learn better when corresponding words and pictures are presented close to each other and simultaneously instead of successively.^72^

The segmentation principle which was applied by Javaid et al.^49^ aims to break large lessons into small learner‐controlled segments.^72^ Lastly, the redundancy principle, which was applied in three studies^49^, ^61^, ^65^ is the principle that aims to minimize the learner's extraneous processing by avoiding the combined use of pictures and text when learners already possess sufficient knowledge.^83^ When learners already possess prior knowledge in a subject, the use of one source only is sufficient as it enables them to build a mental model of what they are presented, hence, the use of a second source is deemed redundant.^83^

As neuroanatomy is often a challenging subject due to the inherent complexity of neuroanatomical structures, it is essential that neuroanatomy educators take into consideration the various principles of multimedia learning and the cognitive load theory when designing and creating new neuroanatomy resources. The use of instructional design principles can ensure the creation of quality online learning resources. Equally important, is for neuroanatomy educators to understand student differences and how their content meets their students' educational needs.^13^, ^49^

### Limitations

One limitation of this systematic review is that only three databases were used to identify relevant articles. This means that potentially eligible studies from other databases may have been overlooked. Additionally, during the initial screening, one non‐English article was excluded, which could be considered another limitation. A further potential limitation is the use of the CASP checklist tool for assessing the risk of bias in the included studies. While the CASP tool is useful, it does not account for factors such as stakeholder opinions, result consistency, or publication bias, unlike the Grades of Recommendation, Assessment, Development, and Evaluation (GRADE) framework, which includes these elements when evaluating the quality of evidence.^84^ A final limitation of this systematic review is that the strengths and weaknesses identified in the included studies during the thematic analysis stage were predominantly determined by the first author's interpretation of each study's results and based on information provided within the studies. This could have introduced subjective interpretation bias although efforts were made to ensure consistency through repeated reviews of the extracted data. The addition of at least one more rater for the identification of the strengths and weaknesses within the included studies could have perhaps strengthened the reliability of the findings.

### Future directions

A neuroanatomy resource that focuses solely on structural neuroanatomy helps medical students develop foundational knowledge in lower‐order cognitive skills, such as recognizing and localizing neuroanatomical structures. However, understanding structural neuroanatomy alone is insufficient for students to grasp how damage to specific structures leads to clinical symptoms. Therefore, future resources should also include a focus on functional neuroanatomy. This would help students develop higher‐order cognitive skills, such as explaining the function of neuroanatomical structures and understanding how their spatial relationships and connections influence these functions. Furthermore, to enable students to apply both their factual (structural neuroanatomy) and conceptual (functional neuroanatomy) knowledge in real‐world clinical contexts, resources should incorporate a clinical application component. By allowing students to connect their understanding of both structural and functional neuroanatomy to clinical scenarios, such resources can bridge the gap between basic neuroanatomy and clinical neurology, potentially reducing the prevalence of neurophobia.

## CONCLUSION

This systematic review indicates that there is insufficient robust evidence to support the effectiveness of neuroanatomy multimedia resources in improving medical students' understanding of neuroanatomy. The current evidence suggests that these resources primarily serve as supplementary learning tools. While they appear useful in enhancing students' knowledge of structural neuroanatomy, they do not adequately address functional neuroanatomy.

The review highlights that 19 out of the 29 resources lack components related to functional neuroanatomy and clinical applications. As these resources focus exclusively on structural neuroanatomy, they fail to help students connect structure with function or apply this knowledge in clinical contexts. The remaining 10 resources primarily focus on structural neuroanatomy but include some functional neuroanatomy content, albeit limited. Only 10 of the 29 resources included a clinical application component, and the quality of this component varied significantly across the resources.

While cadaveric dissection and prosected specimens offer valuable tactile feedback for students manipulating human tissue, they are not ideal for teaching functional neuroanatomy, which largely occurs mainly at cellular and molecular levels. Additionally, this review found that many neuroanatomy multimedia resources lack important multimedia elements, such as sound, animations, and videos. Thoughtful incorporation of these dynamic elements could potentially lead to the development of high‐quality resources that simplify complex neuroanatomical concepts. By enhancing students' understanding of structural and functional neuroanatomy, these resources could ultimately help bridge the gap between neuroanatomy knowledge and clinical symptomatology.

## AUTHOR CONTRIBUTIONS

**Eleni Patera:** Investigation; writing – original draft; writing – review and editing; methodology; formal analysis; data curation; validation. **Mark Pickering:** Supervision; conceptualization; investigation; methodology; project administration. **Thomas Flanagan:** Conceptualization; investigation; writing – original draft; writing – review and editing; supervision; methodology; project administration.

## Supporting information


Appendix A.

Appendix B.

Appendix C.
