# Supplementary material for: Are neuroanatomy multimedia resources effective in improving medical students' understanding of structural and functional neuroanatomy? A systematic review
Source: Anat Sci Educ. 2025 Aug 20;18(10):1029–56. doi: 10.1002/ase.70079 (PMC12511660; doi:10.1002/ase.70079)
Supplement: Supplementary file 1 — Appendix A. [file ASE-18-1029-s002.docx]

**Are Neuroanatomy Multimedia Resources Effective in Improving Medical Students’ Understanding of Structural and Functional Neuroanatomy? A Systematic Review**

**Running title:**

How effective are neuroanatomy multimedia learning resources?

Eleni Patera^1,2^, Mark Pickering^1^, Thomas Flanagan^1^

^1^ Department of Anatomy, School of Medicine, University College Dublin, Belfield, Dublin 4, Ireland

^2^ Human Anatomy Resource Centre, University of Liverpool, Liverpool, United Kingdom

Eleni Patera: [elpa9530@gmail.com](mailto:elpa9530@gmail.com)

Thomas Flanagan: [thomas.flanagan@ucd.ie](mailto:thomas.flanagan@ucd.ie)

Mark Pickering: [mark.pickering@ucd.ie](mailto:mark.pickering@ucd.ie)

**Abstract**

Neuroanatomy education has evolved and improved over the years, driven by advances in technology that have led to the development of innovative interactive digital and immersive learning resources. While neuroanatomy educators have been keeping pace with and harnessing these technology advances, many medical students still struggle to apply their basic neuroanatomy knowledge in a clinical context. Furthermore, the effectiveness of multimedia resources in improving medical students’ understanding of structural and functional neuroanatomy has received limited attention. A systematic review was conducted to document neuroanatomy multimedia resources that were designed for pre-clinical medical students and assessed their effectiveness in improving medical students’ understanding of structural and functional neuroanatomy. Twenty-nine articles were eligible to address the study objective and were appraised according to PRISMA-P guidelines. This review concluded that neuroanatomy multimedia resources are primarily used as supplementary learning tools. However, due to a lack of robust evidence, no definitive conclusions can be made about their effectiveness in enhancing students’ understanding of neuroanatomy. Nevertheless, this systematic review found that most neuroanatomy multimedia resources are effective in supporting students' understanding of structural neuroanatomy but less so for functional and clinical neuroanatomy. Additionally, static multimedia elements were more prevalent than dynamic ones. Moving forward, the thoughtful and informed use of multimedia elements could help establish resources that better integrate structural and functional neuroanatomy with their clinical and real-life applications, ultimately bridging the gap between neuroanatomy and clinical neurology.

**Keywords:** neuroanatomy education; multimedia; medical students; neuroanatomy resources; pre-clinical medical education

**Introduction**

A historical review of neuroanatomy throughout the centuries serves as a reminder of how central the subject of neuroanatomy is to the disciplines of anatomy and clinical neurosciences^1^. Knowledge of both structural and functional neuroanatomy is essential for applying knowledge in clinical neuroanatomy which is concerned with how injury and disease can result in neurologic deficits^2^. Neuroanatomy has been characterized as one of the most challenging components of the anatomy curriculum due to the inherent complexity of specific neuroanatomy concepts and associated neuroanatomical structures^3-6^. Remarkably, neuroanatomy has its own individual place in a medical curriculum, and it is often taught separately from gross anatomy courses as a stand-alone course, with a specific amount of teaching hours dedicated to it and its associated laboratory practicals^7-8^. In recent years, concerns have been raised within the medical profession and medical education about students' inadequate knowledge of neuroanatomy^7,9^. Retention of key neuroanatomy concepts and clinically relevant principles is essential not only for physicians interested in specializing in neurology or neurosurgery, but also for general practitioners who must conduct standard clinical examinations and determine when a patient requires referral to a neurology specialist^10-11^. In addition, there has been a notable reduction in neuroanatomy teaching hours within medical curricula^8,12-13^. Despite this, it remains unclear whether students' limited exposure to neuroanatomy directly leads to weaker knowledge of the subject and whether this, in turn, contributes to junior doctors feeling unprepared to manage neurological conditions.

In 1994, American professor of neurology, Ralph Jozefowicz, introduced the concept of ‘*neurophobia’*, which he defined as “a fear of the neural sciences and clinical neurology that is due to the students’ inability to apply their knowledge of basic sciences to clinical situations”^14^. Since the introduction of the concept of neurophobia, extensive research has been conducted to identify its causes, the factors contributing to difficulties in learning and understanding neuroanatomy, and the educational interventions that could help mitigate neurophobia^4,15,17-22^. In the future, the burden of neurological diseases is expected to intensify as major disorders affecting the nervous system continue to rise^16^. The failure to recognize the implications of neurophobia in medical education could lead to a shortfall of practising neurologists in the future^20^.

Medical educators are facing growing pressure to address neurophobia by reforming neurology education, given its perceived impact on discouraging medical students from pursuing neurology as a career specialty. In 2021, Omar discussed various teaching strategies that could help tackle neurophobia^23^. Some strategies include horizontal or vertical integration in medical education^23^. Furthermore, a study showed that vertical integration in neuroanatomy teaching resulted in students feeling more prepared for clinical neuroscience-related concepts (e.g., interpreting MRI and CT images) once they entered the clinical years of their degree^24^.

**Impact of technological advances on neuroanatomy education**

Advances in educational technology have significantly transformed neuroanatomy instruction, shifting from traditional methods to modern, technology-based teaching approaches and resources^25^. New technologies (e.g., synchronous or asynchronous digital classrooms, virtual learning environments, online interaction tools) are promising, offering the potential to simplify students' access to teaching and learning materials, enabling self-paced learning, and enhancing the overall learning experience^26,27^.

In recent decades, online learning, defined as education that occurs over the Internet^28^, has been incorporated into neuroanatomy education. The terms "e-learning" (electronic learning) and "computer-assisted learning" (CAL) are often used interchangeably with online learning. Multimedia CAL resources have gained increased attention in recent years^29-30^. Multimedia technology is defined as “the use of a computer to present and combine graphics, audio, and video, with links and tools that let the user navigate, interact, create, and communicate” ^31^. Neuroanatomy educators can use multimedia elements such as audio, images, text, and videos during instruction, potentially simplifying complex concepts for students.

Advances in technology have led to the creation of both non-digital resources, such as 3D-printed and 3D plastic models, and digital resources ranging from simple multimedia tools to more complex immersive technologies such as virtual reality (VR), augmented reality (AR), mixed reality (MR), stereoscopy, and 3D digital atlases^25^. Despite these advances, educators must exercise caution when integrating such technologies into their teaching. Educators need to ensure that any technological resource used for instruction or assigned to students is not overly complex and that students have sufficient time to engage with the resource. Insufficient exposure to these resources may hinder students' learning and their ability to engage effectively. When designing a resource, educators must ensure it is purposefully aligned with its intended goals. Key questions to consider include: 1) What do students find challenging and why? 2) What features are required to simplify a topic? 3) How does the resource enhance the students’ learning experience? 4) Does the resource content constructively align with the learning objectives on which students will be assessed?

**Research focused on the neuroanatomy teaching methods, tools and their impact on students’ learning**

Currently, in the literature, there are three systematic reviews focusing purely on neuroanatomy education. In 2018, Arantes et al. published a systematic review to explore neuroanatomy teaching methods and assess their impact on learning, with the ultimate goal of providing guidance for curricular improvement^32^. This systematic review included 29 articles for final analysis and identified 15 teaching methods that were classified as digital and non-digital tools. The number of articles focusing on digital tools and non-digital tools was 14 for both categories. Most of the six studies focusing on computer-based digital tools found that these tools were effective for both students and faculty, with students showing a positive attitude toward using them and demonstrating improved performance in their quiz or test scores. However, two studies revealed no statistically significant differences in students’ scores after the introduction of computers into the course. Furthermore, students reported lower scores on deep approach for the computer-based course compared to general studies (CAL Course: 25.55; General studies: 27.44). While this systematic review included studies involving medical students and students from allied health professions, a limitation was that it did not include research on the use of virtual or augmented reality in neuroanatomy education.

In 2020, Sotgiu et al. published a systematic review aimed at identifying the most effective methods for teaching human neuroanatomy, focusing on the challenges it poses for medical students^5^. Newman et al. pointed out a major limitation of the review by Sotgiu et al., noting that it did not include any studies discussing VR or AR modalities^33^. While few in number, Sotgiu et al. did include two studies in their review - one focussing on VR and the other on mobile augmented reality (mAR)^5^. However, as Chytas et al.^34^ highlighted, a major limitation of this review is that, despite the assertion of Sotgiu et al.^5^ that a combination of teaching methods is needed in neuroanatomy education, the authors concluded that cadaveric dissection remains the “gold standard” method for teaching neuroanatomy. Chytas et al.^34^ argued that this conclusion was not evidence-based, as only two out of the 18 studies included in the review focused on cadaveric dissection. Moreover, while Sotgiu et al.^5^ referenced various studies that showed positive outcomes favouring cadaveric dissection, none specifically examined its effectiveness in neuroanatomy education specifically. Furthermore, the results did not demonstrate that cadaveric dissection leads to superior knowledge acquisition compared to other educational methods^32^.

In 2022, Newman et al.^33^ conducted a focused review to explore the technology-enhanced teaching methods currently available to neuroanatomy educators. Their review aimed to compare traditional teaching methods with technology-based approaches, identify those associated with improved knowledge acquisition and long-term retention, and understand why some teaching methods are effective in particular contexts while others are not. Newman et al.^31^ noted that the systematic reviews by Arantes et al.^32^ and Sotgiu et al.^5^ lacked adequate descriptions of the resources they examined and failed to provide sufficient context to explain why certain resources yielded better outcomes than others. In response, Newman et al.^33^ proposed four potential explanations for why certain resources appear more effective in helping students to acquire and retain neuroanatomy knowledge over time. However, a potential limitation of their review is that it did not include CAL as one of the technology-enhanced teaching methods for neuroanatomy education.

Current evidence in the literature demonstrates that there are various innovative tools, resources, and teaching methods available for neuroanatomy education. Despite this, the use of complex technological equipment does not automatically lead to better learning outcomes. There has been insufficient focus on the effectiveness of multimedia resources in improving medical students’ understanding of both structural and functional neuroanatomy. It remains unclear whether these technology-enhanced neuroanatomy resources are designed in a way that promotes deep learning while also helping students apply their knowledge of basic sciences in clinical contexts.

A systematic review that evaluates the effectiveness of neuroanatomy multimedia resources in improving medical students’ understanding of neuroanatomy – by examining the outcomes of each study and identifying how different components of the resources support student learning - has not yet been published. Such a review would be valuable for neuroanatomy educators, as it could highlight potential inefficiencies in current neuroanatomy multimedia educational resources. This insight could help educators design more effective resources that aid students in mastering structural neuroanatomy, which forms the foundation for understanding functional neuroanatomy, ultimately supporting their progression through various levels of knowledge while using the resource.

The aim of this systematic review is to address the following research question: ‘Are neuroanatomy multimedia resources specifically designed for pre-clinical medical students effective in improving their understanding of structural and functional neuroanatomy?’

**Materials and Methods**

**Protocol review**

This systematic review was guided by the Preferred Reporting Items for Systematic Reviews and Meta-Analyses Protocols (PRISMA-P)^35^ guidelines.

**Literature search and databases**

The bibliographic databases that were used for this systematic review were PubMed (United States National Library of Medicine, Bethesda, MD), Scopus^TM^ (Elsevier B.V., Amsterdam, The Netherlands) and Education Resources Information Center (ERIC) (Institute of Education Sciences [IES], Washington, DC). The following combination of search strings was used to identify relevant studies: "neuroanatomy" AND ("learning" OR "e-learning" OR "education" OR "teaching" OR "multimedia" OR "multi-media" OR "video" OR "resource" OR "interactive" OR "animation" OR "virtual reality" OR "augmented reality" OR "online" OR "student"). No distinction was made in the term ‘neuroanatomy’ to ‘structural’ and ‘functional’ as the use of the terms ‘structural neuroanatomy’ OR ‘functional neuroanatomy’ yielded results that focused on neurophysiology or neuropathology and not on neuroanatomy education. The comprehensive search strategy for the PubMed database can be seen in **Appendix A.**

The search utilized the “Article Type” filter on PubMed and Scopus, which was unavailable on the ERIC database. The filter was limited to journal articles, including original research, descriptive articles, book chapters, and short communications. Additionally, the “Publication Date” filter was applied to limit the search from 1994 to 2024. This date range was chosen because the concept of neurophobia was first introduced in 1994. Initially, only these two filters were applied during the electronic search to export the identified studies from each database and import them to the Covidence software (*Veritas Health Innovation, Melbourne, Australia*). Only studies published in English were considered, as this is the language of the authors. The study selection process began on September 24, 2021, and ended on December 31, 2024.

**Study selection criteria and screening procedures**

The review followed the Patient/Population, Intervention, Comparison and Outcomes and Study (PICOS) model to define the criteria for study eligibility^36^: population (pre-clinical undergraduate or graduate medical students), exposure (multimedia VR, AR, MR or CAL resources), comparator (multimedia VR, AR, MR, CAL resources or no comparator), outcomes (any multimedia neuroanatomy resource that was specifically designed for pre-clinical medical students that assessed its impact on students’ understanding of neuroanatomy), publication type (journal research articles and book chapters), study design (( e.g., randomized controlled trials (RCTs), mixed-methods)), language (English) and year of publication (April 1994 to December 2024). The inclusion and exclusion criteria for both study eligibility and report eligibility are demonstrated in **Table 1**.

**Table 1:** Inclusion and exclusion criteria for each domain of PICO(s) and other eligibility variables in relation to the research question. VR: Virtual Reality; AR: Augmented Reality; mAR: mobile Augmented Reality; CAL: Computer-Assisted-Learning; 3D: Three-dimensional; PICO(s): Patient/Population, Intervention, Comparison and Outcomes and Study.

Footnote:

VR: Virtual Reality

AR: Augmented Reality

mAR: mobile Augmented Reality

CAL: Computer-Assisted-Learning

3D: Three-dimensional

PICO(s): Patient/Population, Intervention, Comparison and Outcomes and Study.

Two independent reviewers (EP, TF) performed the study selection in two screening stages. First, the titles and abstracts of the articles were reviewed. Full texts were retrieved only when inclusion could not be determined from the title and abstract or if no abstract was available. In the second stage, full texts were assessed for inclusion. Discrepancies between reviewers were resolved by a third reviewer (MP). Articles excluded from the review were rejected by at least two of the three reviewers.

**Data extraction**

The first author (EP) collected data from each included study. Key study characteristics such as the authors, publication year, country where study was conducted at, study design, participant cohort, study aims, type of multimedia resource and outcomes regarding resource’s impact on students’ understanding of neuroanatomy were tabulated in **Table 2**. The PICO(s) model was used to extract important information, including the study aim, design, population, exposure, comparator, measures, and outcomes (**Table 1, Appendix B**). The number of multimedia elements across each resource, such as images, text, audio, video, animations, and manipulable models (e.g., rotation, zooming), was also determined by reviewing descriptions in the articles, screenshots of the resource, supplementary materials, or by accessing the resources online. Bar charts were created to visually compare the multimedia elements present or absent in each resource. Thematic analysis was conducted after assessing the articles, identifying strengths and weaknesses in the resources and the corresponding studies. The strengths and weaknesses were identified predominantly based on the primary author’s (EP) interpretation of the information presented within each study and resource if it was accessible, rather than being directly extracted verbatim. The interpretation focused on each study’s materials and methods, results, discussion and conclusion. Recurring themes were noted across each study’s methodological quality and clarity of result reporting. In cases where limitations were not explicitly acknowledged by the authors of each study, they were inferred by the primary author.

**Risk of bias assessment**

To assess the validity of the studies, the primary reviewer (EP) conducted a risk of bias assessment using the Critical Appraisal Skills Program (CASP) checklists^37^. The CASP RCT and cross-sectional studies checklists, which include 11 questions for evaluating bias and study quality, were applied to the RCTs, mixed-methods and cross-sectional studies included in the review.

**Results**

**Study selection**

The electronic search across three databases identified a total of 5,180 articles. Subsequently, these files were imported into Covidence to remove any duplicates before commencing the screening process. After duplicates were removed, 4,400 articles were screened based on their titles and abstracts. Each abstract was reviewed for the inclusion criteria, leaving 127 articles for secondary screening. During the secondary screening, the full texts of the 127 articles were reviewed and 98 articles were excluded as they did not directly address the research question of this systematic review. The reasons for excluding studies during the secondary screening process are detailed in the PRISMA-P flow chart (**Figure 1**). Ultimately, 29 articles were selected for final data extraction (**Table 2**).

**Table 2:** Characteristics of included studies

Footnote:

AR: Augmented reality

CAL: Computer-assisted learning

E-learning: Electronic learning

LFB: Luxol fast blue

mAR: Mobile augmented reality

MCQ: Multiple-choice-questions

MR: Mixed reality

MRT: Mental rotation test

N/A: Not applicable

PLI: Polarized light imaging

VR: Virtual Reality

2D: Two-dimensional

3D: Three-dimensional

**Study characteristics**

The 29 studies included in the review were published between 1997 and 2024, with the majority published in 2024 (**Table 2**). These studies originated from 14 countries: the United States (n=6)^42,43,46,48,50,56^, Canada (n=4)^39,44,47,57^, United Kingdom (n=4)^38,40,49,67^, Netherlands (n=3)^52,55,73^, France (n=1)^54^, Republic of Ireland (n=1)^51^, Italy (n=1)^72^, Republic of Korea (n=1)^69^, China (n=1)^68^, Brazil (n=1)^41^, Spain (n=1)^45^, Germany (n=1)^53^ , India (n=2)^70,71^ and Turkey (n=2)^58,74^ (**Table 2**).

Among the 29 studies, seven studies^38, 39,40,41,67,73,74^ focused solely on evaluating the impact multimedia resources have on students’ understanding of neuroanatomy, while the remaining 22 studies assessed both students’ perceptions (in terms of satisfaction or motivation) and the impact of multimedia resources on their understanding of neuroanatomy^42,43,44,45,46,47,48,49,50,51,52,53,54,55,56,57,58,68,69,70,71,72^. Of these 29 studies, 18 were randomized control studies^39,40,41,44,46,47,51,52,53,54,55,56,57,68,69,70,71,73^, one was a mixed-methods study^58^  , one was a cross-sectional study^67^ and nine studies did not specify their study design^38,42,43,45,48,49,50,72,74^. The study characteristics for each article were extracted using the PICO(s) model, and are summarized in **Table 1**. A summary of each study can be found in **Table 1** (**Appendix B**).

**Multimedia technologies used**

The 29 studies identified five types of multimedia technology resources: 1) Virtual Reality (VR), 2) Augmented Reality (AR), 3) Mixed Reality (MR), 4) Stereoscopy and 5) Computer-Assisted Learning (CAL) resources (**Figure 2**). Of the 29 studies, 16 described CAL resources^38,39,42,43,44,45,46,47,48,49,50,51,52,67,68,69^, four described stereoscopic resources^41,53,54,70^, three described VR resources^56,57,71^ five described AR resources^55,58,72,73,74^ and one study described an MR resource^40^.

**Study participants**

The studies included data from a total of 4,607 students. Of these, 1,855 were first-year undergraduate and graduate medical students, 2,377 were second-year undergraduate medical students, and 26 were health-allied profession students. Additionally, 135 students were classified as graduate medical students, 115 students as medical students, and five as upper-level undergraduate medical students although their exact year of study was not specified. One study included five first-year medical and biomedical sciences students and 23 second-year medical and biomedical sciences students, however, the exact number of medical and biomedical students within each year was not provided. Another study had 66 participants which were first- and second-year medical students, however, the exact number of medical students within each year was not provided. Hence, the corresponding authors and co-authors of the studies where the participants’ year of study was not specified, have been contacted via email or on ResearchGate to ask for clarifications, however, no responses have been received.

**CAL resources**

Sixteen studies described the use of computer-assisted learning (CAL) resources^38,39,42,43,44,45,46,47,48,49,50,51,52,67,68,69^. In 13 of these studies, the CAL resource was assessed in terms of medical students’ perceptions of the resource and its impact on their understanding of neuroanatomy^42,43,44,45,46,47,48,49,50,51,52,68,69^, while the remaining three studies focussed solely on the impact of the CAL resource on students’ understanding of neuroanatomy only^38,39,67^.

In eight studies, participants were exposed to a CAL resource without any comparator^38,39,42,43,45,47,50,52^. In one study^39^, where second-year health science students and second-year undergraduate medical students were involved, a comparator was used only for the health science group. In the remaining eight studies, a comparator was included^44,46,48,49,51,67,68,69^. These comparators varied from neuroanatomy textbooks^44,67,68^, traditional neuroanatomy lectures^46,68^, conventional teaching materials^48^, traditional neuroanatomy courses^49^, interactive CAL neuroanatomy resource^51^, to a donor dissection^69^. Fourteen out of the 16 CAL resources were designed as adjuncts to support neuroanatomy learning^38,39,43,44,45,46,47,48,49,50,51,52,67,68^, whereas only one resource aimed to replace traditional teaching materials entirely^42^. One study utilized a CAL resource as an adjunct with the potential to replace donor dissection later on^69^.

The impact of CAL resources on students’ understanding of neuroanatomy was assessed in 16 studies, nevertheless, only four studies^47,51,52,67^ employed pre- and post-tests that allowed for measurable changes in students’ understanding. Among these, three studies^47,51,67^ reported statistically significant improvements in students’ post-test scores after exposure to the CAL resource. In the study by Allen et al.^47^, group A, which used the CAL resource before attending the cadaveric laboratory session, scored significantly higher than group B, who accessed the resource afterward. Furthermore, only group A’s final knowledge assessment scores were significantly higher (p<0.01) compared to their first post-test scores. The study by Javaid et al. ^51^ found statistically significant differences in pre- and post-test scores across three groups: an experimental group using an interactive CAL resource on spinal pathways, a control group using a functional neuroanatomy resource, and a no-use group. The learning gain for the experimental group was significantly higher than the no-use group (p=0.04). In the study by Booker et al.^67^, students in the intervention group who used CAL video resources from the Soton Brain Hub online educational platform showed statistically significantly higher average learning gains than the text-based resource control group (p=0.030). Nevertheless, no statistically significant differences were observed in the retained learning gains between the two groups on the retention MCQ test (p=0.919) that participants took 3 weeks after accessing their assigned resource.

All eight studies^44,46,48,49,51,67,68,69^ that used comparators found statistically significant differences between the CAL resource and the comparator in improving students’ understanding of neuroanatomy. In the study by Svirko and Mellanby^49^, a positive correlation was observed between students’ deep engagement with the CAL course and their performance on the formative neuroanatomy assessment (p<0.001; r=0.12). Drapkin et al.^46^ found that the experimental group, which used CAL resources, outperformed the control group in questions involving C-shaped structures, with an average score difference of 15.8% (p<0.01). In the study by Lewis et al.^44^, the intervention group achieved significantly higher mean raw scores than the control group (p=0.028). Peterson and Mlynarczyk^48^ observed that student performance on questions related to material taught using 3D teaching tools was significantly better (p<0.0001) compared to material taught using traditional teaching methods. Xuan et al.^68^ found that the experimental group, which used the GRAVEN database that consists of pictures of hand gestures that simulate intracranial arteries and veins and neurosurgical approaches, scored statistically significantly better than the control group who only studied neuroanatomy using traditional neuroanatomy teaching methods (PowerPoint and textbooks) (p=0.0026). Yun et al.^69^ conducted two post-tests (quizzes 1 and 2) after the donor group and the virtual group accessed donor dissections and the CompleteAnatomy app, respectively. Yun et al.^69^ found that the virtual group which accessed the CompleteAnatomy app on a tablet scored statistically significantly higher in Quiz 1 than the donor group (p<0.05). Despite this, the authors reported no statistically significant differences between the two groups for Quiz 2.

**Stereoscopic resources**

Four studies described the use of stereoscopic resources^41,53,54,70^. Three of these studies evaluated both students’ perceptions of the stereoscopic resource and its impact on their understanding of neuroanatomy^53,54,70^, while the fourth study^41^ focused solely on the impact of the stereoscopic resource on students’ understanding only. In three of the studies, the stereoscopic resource was compared to a non-stereoscopic resource^53,54,70^, while the fourth study did not include a comparator^41^. Pre- and post-tests were used in three studies^41,54,70^, whereas the remaining study only administered a post-test after students were exposed to the stereoscopic resource^53^.

In the study by Bernard et al.^54^, students who used a stereoscopic resource demonstrating the circle of Willis scored statistically significantly higher (p=0.01) on tests of anatomical relations and clinical reasoning compared to those using a 2D resource. Similarly, in the study by de Faria et al. ^41^, students in the experimental groups – one using an interactive non-stereoscopic lecture and the other using an interactive stereoscopic lecture, both on the limbic system - scored statistically significantly higher (p<0.05) than students in the control group, who received a traditional 2D lecture on the limbic system. Both experimental groups showed significant improvement in post-test scores compared to pre-test scores, and there was a significant difference in practical exam scores between the control group and the experimental groups. However, no statistically significant advantage was found for the stereoscopic method over the non-stereoscopic one (p>0.05). In the study by Yohannan et al.^70^, students in the stereoscopic group who received a 20-minute demonstration on the brainstem via the AnaVu stereoscopic resource scored statistically significantly higher in the basic recall questions than students in the monoscopic group (p=0.03) and control group (p=0.001) who received the same demonstration in monoscopic mode and white-board drawn diagrams, respectively. Also, students in the stereoscopic group scored statistically significantly higher in the radiological questions than students in the control group (p<0.001). In the study by Kockro et al.^53^, where students completed a post-test after using either a 2D non-stereoscopic or a 3D stereoscopic resource focused on the anatomy of the third ventricle, no significant differences were found between the post-test scores of the stereoscopic and non-stereoscopic groups.

**Virtual reality (VR) resources**

The impact of VR resources on medical students’ understanding of neuroanatomy was evaluated in three studies using pre- and post-tests^56,57,71^. Two studies found no statistically significant differences between pre- and post-test scores ^56,57^. In the randomized controlled study by Stepan et al.^56^, first- and second-year medical students were randomly assigned to either a VR experimental group or a control group, which used unspecified study materials to learn about the ventricles and vasculature of the brain. No significant differences were observed between the VR and control groups in their pre-intervention or retention quiz scores. However, second-year medical students scored significantly higher than first year medical students in all three quizzes. Similarly, in the study by Ekstrand et al.^57^, first- and second-year medical students were randomly assigned to either a VR experimental group or a control group using paper-based materials. The resources covered the basal ganglia, adjacent neuroanatomical structures, and the lateral corticospinal and spinothalamic tracts. No significant differences were found between the experimental and control groups in their pre-test and two post-intervention test scores. In the third study that evaluated the impact of a VR resource on students’ understanding of neuroanatomy, a pre-intervention test and two post-intervention tests were employed^71^. The authors reported that male and female participants in the control group who used paper-based methods to study the neuroanatomical structures of interest, showed a statistically significant improvement from their pre- to post-test scores, while male and female participants in the intervention group who used a VR resource scored significantly lower in their post-test compared to their pre-test scores. However, participants in both groups demonstrated a statistically significant improvement in their second post-test to their first post-test. Despite this, the authors did not conduct statistical comparisons between the two groups, nor did they analyze the results collectively for male and female participants within each group.

**Augmented reality (AR) resources**

The impact of augmented reality (AR) resources on students’ understanding of neuroanatomy was assessed in five studies^55,58, 72,73,74^, four of which employed pre- and post-tests^55,58,73,74^. In two out of these four studies, no statistically significant differences were found between the pre- and post-test scores of the experimental and control groups ^55,58^. One study did not report whether there were any statistically significant differences in the pre-test scores of the experimental and control groups^73^. In the remaining study^74^, a statistically significant difference was observed in the pre-test scores of the two intervention groups.

In 2016, Kücük et al.^58^ evaluated the effects of learning neuroanatomy using a mobile AR app on second-year medical students’ academic performance and cognitive load. Students were assigned to either the experimental group, which used the mAR app to review material on the ascending and descending pathways, or the control group, which used a traditional neuroanatomy textbook. Univariate ANOVA statistical analysis revealed that the experimental group scored significantly higher (p<0.05) than the control group on the academic achievement test. Additionally, students in the experimental group reported significantly lower cognitive load scores compared to those in the control group. Henssen et al.^55^ examined differences in test scores, cognitive load, and motivation among first-year medical and biomedical sciences students. Participants were assigned to either the experimental group, which used the GreyMapp-AR app to study subcortical structures, or the control group, which used cross-section anatomy drawings. While both groups showed significant improvement in their post-test scores compared to their pre-test scores, no statistically significant differences were observed between the two groups. Interestingly, the control group outperformed the AR group on the post-test (p<0.01). Further analysis revealed that the control group scored significantly better on the third part of the test, which focused on cross-sections. Both groups experienced higher cognitive load scores (p=0.039) after completing the practical assignments. Overall, post-test scores improved for both groups, regardless of the resource used. A similar study conducted by Zeedzen-Scheffers et al.^73^ examined the effectiveness of using the GreyMapp AR resource versus an anatomical atlas in preparing 28 first- and second-year medical and biomedical sciences students for their neuroanatomy prosection-based practicals. The intervention group had access to the GreyMapp AR resource to complete their preparatory assignment prior attending their body donor-based education. Students received an overview of the anatomy of the human brain for their preparatory assignment. The control group had access to an anatomical atlas resource to complete their preparatory assignment prior attending their body donor-based education. No statistically significant differences were observed in the pre- and post-test scores of two groups (p=0.35). In the study by Cercenelli et al.^72^, the effectiveness of the AEducAR3.0 hybrid platform which combines AR with 3D printed models was investigated among 70 second year medical students. The AEducAR3.0 platform aims to allow students to study neuroanatomy at three different learning levels (notional learning, notional learning in context and topographical learning). In this study, there was no comparator present, and participants took a self-assessment quiz at the end of each learning level. When considering all three learning levels, 51% of students scored above the sufficient threshold (9.6/16) with a median score of 10, and while the quiz difficulty was overall well-balanced across all three learning levels, students performed significantly better in the quiz of learning level 2 compared to learning level 1 (p=0.04) and 3 (p= 0.02). A single study conducted by Gurses et al.^74^, investigated the effectiveness of teaching neuroanatomy using AR and VR resources when cadaveric dissection was not available as a neuroanatomy teaching method. This study did not use a comparator; however, the study participants who were neurosurgery residents (N= 40) and second-year medical students (N=200) accessed VR-based 3D models and AR-based 3D models, respectively. Participants took a pre- and post-test. Neurosurgery residents scored higher than medical students in the pre-test (7.5/10 vs 4.8/10). Both neurosurgery residents and medical students scored statistically significantly higher in their post-test scores. p<0.001).

**Mixed reality (MR) resources**

Pickering et al.^40^ explored the impact of a mixed reality (MR) resource on medical students' understanding of neuroanatomy, focusing on the corticospinal tract, spinothalamic tract, dorsal column tract, and trigeminothalamic tract. This was compared to a screencast-based multimedia video resource. Students exposed to either the MR resource or the screencast showed significant improvement in their post-test scores across multiple sections, including multiple-choice questions (MCQs), short answer questions (SAQs), and overall scores (MR: p<0.001; screencast: p<0.001). However, no significant differences were observed in the pre-test scores between the two groups. Although both resources led to improved knowledge retention, the anatomy drawing screencast was found to have a greater effect on students’ retention of information. Nevertheless, when learning gains were measured using both absolute and normalized scores, only the MCQ section of the post-test showed a statistically significant improvement for the screencast group (absolute gain: p=0.003; normalizing gain: p=0.001).

**Multimedia elements across resources described in the included studies**

The ‘image’ multimedia element was present in all 16 CAL resources^38,39,42,43,44,45,46,47,48,49,50,51,52,67,68,69^ , all three VR resources^56,57,71^, all five AR resources^55,58,72,73,74^, all four stereoscopic resources^41,53,54,70^ and single MR resource^40^. The ‘text’ element appeared in 13 out of 16 CAL resources^38,39,42,43,45,47,49,50,51,52,67,68,69^, three of the four stereoscopic resources^53,54,70^, all three VR resources^56,57,71^, all five AR resources^55,58,72,73,74^ and the single MR resource^40^. The ‘sound’ element was present in three CAL resources ^50,51,67^, in two stereoscopic resources^53,54^ and the single MR resource^40^. However, neither the AR resources^55,58,72,73,74^ nor the VR resources^56,57,71^ contained the ‘sound’ element. The ‘video’ element was present in six CAL resources^39,48,49,50,51,67^, two stereoscopic resources^41,54^, one AR resource^58^, one VR resource^71^ and the MR resource^40^. The ‘animations’ element appeared in eight CAL resources^38,42,43,48,50,51,67,69^, in three stereoscopic resources^41,53,54^, two AR resources^58,72^, and the MR resource^40^. None of the VR resources^56,57,71^ included the ‘animations’ element. The ‘model manipulation’ element was found in six CAL resources^43,45,46,47,48,69^, in two stereoscopic resources^53,70^ in two VR resources^56,71^, and in three AR resources ^55,72,73^. The MR resource^40^ lacked the ‘model manipulation’ element. A detailed representation of the multimedia elements present or absent in each resource can be seen in **Figure 3**.

**Effectiveness of neuroanatomy multimedia resources on medical students’ understanding of neuroanatomy**

Out of the 29 articles reviewed to answer the research question, 15 studies administered a pre- and post-intervention test to assess the effectiveness of multimedia resources on students’ understanding of neuroanatomy^40,41,47,51,52,54,55,56,57,58,67,70,71,73,74^. Among these 15 studies, only seven showed a statistically significant improvement in students’ understanding after exposure to the multimedia resource^41,47,51,54,70,73,74^. Three of these studies focussed on stereoscopic resources^41,54,71^, one study focused on an AR resource ^73^, two studies focused on CAL resources^47,51^ and one study focused on an AR and a VR resource^74^.

**Risk of bias within studies**

Of the 29 studies included in the final analysis, 20 were eligible for risk of bias assessment using the CASP checklist, which included the 18 randomized controlled studies, a single mixed-methods study and a single cross-sectional study. A complete risk assessment could not be performed due to insufficient information, with many answers marked as “No” or “Cannot tell”. Despite this, individual types of bias - such as selection, performance, detection, and reporting bias – were assessed **(Articles 1-19, Table 1, Appendix C, Article 20, Table 2, Appendix C)**.

Out of the 20 eligible studies, only one study exhibited selection bias as its participants were neurosurgery residents and medical students, with each group accessing a different type of an immersive multimedia resource ^74^. A high risk of performance bias was identified in one study^39^. Seven studies ^39,41,44,55,58,71,74^ showed a relatively high risk of reporting bias. For detection bias, it was unclear in all 20 studies whether participants, investigators, and assessors were blinded to the intervention and outcomes, as the responses to question 4 and its sub-questions (4a, b, c) were predominantly “No” or “Cannot tell”. A summary of the risk of bias assessment for the eligible studies can be found in the supplementary material (**Tables 1-2, Appendix C**).

**Common themes across resources and the articles describing them**

Thematic analysis of the included articles revealed both strengths and weaknesses in the resources and the articles that described them, which are outlined in **Tables 3** and **4**, respectively.

**Table 3:** Summarizes four strengths that were identified in some of the resources described in the 29 articles that were included in the final analysis.

Footnote:

AR: Augmented reality

CAL: Computer-assisted learning

CT: Computed tomography

MR: Mixed reality

MRI: Magnetic resonance imaging

VR: Virtual Reality

**Table 4:** Summarizes 12 existing weaknesses present across some of the resources described in the 29 articles that were included in the final analysis.

Footnote:

AR: Augmented reality

CAL: Computer-assisted learning

MR: Mixed reality

VR: Virtual Reality

Regarding the strengths of some resources, four key advantages were identified: i) exposure to radiological imaging scans (e.g., CT, MRI), ii) inclusion of interactive features (e.g., quizzes, feedback, diagrammatic trees), iii) incorporation of clinical application elements, and iv) design based on evidence-based principles **(Table 3)**. However, only eight out of the 29 resources exposed students to radiological imaging scans^39,44,45,46,48,70,71,73^; eight included interactive features^38,42,43,49,50,51,52,69^; 10 featured a clinical application element^38,40,42,43,44,49,51,67,68,72^; and 10 were designed based on evidence-based principles^40,43,47,51,52,55,58,67,70,73^.

In terms of weaknesses in the resources or the articles, 12 areas of concern were identified (**Table 4**). The four most common weaknesses were: i) 22 of the 29 resources could not be accessed^38,39,40,41,42,43,45,46,47,48,49,52,53,55,56,57,58,69,70,71,73,74^; ii) 19 articles focused solely on structural neuroanatomy^38,39,40,41,45,46,47,52,53,54,56,57,58,68,69,70,71,73,74^; iii) 17 resources contained fewer than three multimedia elements^38,39,41,42,44,45,46,47,49,52,55,56,57,68,70,73,74^; and iv) 14 articles lacked sufficient information in the methods section, such as details on the design and structure of the pre-test/post-test assessments, the time allocated for completing tests, questionnaires, or surveys^38,39,41,42,47,49,50,52,55,56,58,67,68,71^.

**Discussion**

This systematic review aimed to answer the research question of whether neuroanatomy multimedia resources designed for pre-clinical medical students are effective in improving their understanding of structural and functional neuroanatomy. Unlike previous reviews, this study highlighted a key limitation across the currently documented multimedia neuroanatomy resources: most of these resources primarily focus on structural neuroanatomy, with limited attention given to functional and clinical neuroanatomy.

**Effectiveness of neuroanatomy multimedia resources on students’ understanding**

Out of the 29 articles included in this review, only seven studies^41,47,51,54,70,73,74^ demonstrated a statistically significant improvement in students’ understanding of neuroanatomy after exposure to multimedia resources. Three of these studies evaluated a stereoscopic resource^41,54,70^, two evaluated a CAL resource^47,51^, one evaluated an AR resource ^73^, and one evaluated a VR and an AR resource^74^.

In the study by Allen et al. ^47^, the CAL resource focused solely on structural neuroanatomy. In contrast, the study by Javaid et al. focused on structural, functional, and clinical neuroanatomy. A systematic review published in 2009 assessed whether computer-aided learning facilitates anatomy education^59^. This review analyzed eight quantitative studies, all of which showed positive results favouring the use of computer-aided-learning resources. However, only one study specifically focussed on a neuroanatomy resource, which was found to have potential as an adjunct tool for improving students’ knowledge^59^. The review concluded that computer-aided learning can enhance anatomy education when well-designed and integrated into the medical curriculum^59^.

Four studies evaluated the effectiveness of stereoscopic 3D resources in enhancing medical students’ understanding of neuroanatomy^41,53,54,70^. While three studies^41,54,70^ found statistically significant improvements in students’ scores, the literature suggests that stereoscopic resources are particularly effective in improving spatial visualization of neuroanatomical structures and clinical reasoning^53,54,61^. The results from Yohannan et al. ^70^. complement the literature, as the authors reported that the AnaVu stereoscopic tool helped students improve their visuo-spatial skills. Moreover, the authors reported that the stereoscopic group yielded a greater effect size regarding the tool’s ability to teach radiological anatomy due to the nature of stereopsis integrating spatial concepts better^70^. However, Bogomolova et al. ^60^ emphasized that future research should consider students’ visuo-spatial abilities when assessing the effectiveness of stereoscopic tools. The three studies that showed positive results^41,54,70^ did not account for the potential disadvantage faced by students with lower visuo-spatial abilities, which may have impacted the outcomes. Interestingly, none of these three studies employed specific psychometric tests such as mental rotation tests to assess students’ spatial reasoning.

In the study that evaluated the use of an AR and a VR resource there were two experimental groups^74^. Neurosurgery residents accessed a VR resource, and medical students accessed an AR resource. The authors reported that neurosurgery residents had statistically significantly higher pre-test scores than medical students. A potential justification as to why neurosurgery residents had a higher baseline knowledge than medical students might be their extensive substantial clinical experience and knowledge. In this study^74^ even though the two groups accessed a different type of a multimedia resource, both groups scored statistically significantly higher in their post-test than in their pre-test. Despite this, the authors do not provide any information on whether a comparison was made between the post-test scores of the two groups. In the study by Zeedzen-Scheffers et al.^73^, the AR group accessed the GreyMapp resource prior attending their body donor-based education whereas the control group accessed the Sobotta anatomical atlas. The study showed that both groups showed a statistically significant improvement from pre- to post-test scores nevertheless, when the MRT and pre- and post-test scores of the two groups were compared, no statistically significant differences were observed^73^. In September 2024, Salimi and colleagues^75^ conducted a systematic review and a meta-analysis of 24 randomized controlled trials to assess the effectiveness of AR and VR in anatomy education. The systematic review and meta-analysis showed that VR improved knowledge scores to a moderate extent, while AR did not show a significant effect. Moreover, VR was rated as more ‘useful’ compared to other methods nevertheless, the high heterogeneity among studies suggests that further research is needed to clarify the factors that influence these technologies^75^.

**Multimedia elements in neuroanatomy resources**

The multimedia elements present in neuroanatomy resources varied across the studies, with the ‘image’ element being the only one consistently included in all 29 resources. The ‘text’ element appeared in 25 resources, while the ‘video’ and ‘model manipulation’ elements were found in 11 and 13 resources, respectively. The ‘animation’ element was present in 14 resources, and the ‘sound’ element was included in just six. Interestingly, neither the augmented reality (AR) nor the virtual reality (VR) resources featured the sound element.

Auditory formats in multimedia resources can range from narration to music and sound effects^62^. Narration, in particular, supports verbal learning, where students absorb information through or text displayed on the screen, such as bullet points^63^. Including narration in neuroanatomy resources can be especially beneficial, as it allows the educator to guide students through various neuroanatomical structures. This is crucial for students with limited or no prior knowledge of neuroanatomy, as they may struggle to identify and orient structures in brain cross-sections. Additionally, the use of narration can be useful in improving students’ understanding of the function of neuroanatomical structures. For example, the computer-aided learning (CAL) resource created by Javaid et al.^51^ focused on spinal pathways and incorporated multimedia elements like text, images, and animations. These elements enabled students to visualize and trace sensory neurons from the spinal cord to the medulla oblongata, the thalamus, and finally the primary somatosensory cortex. In specific sections, narrated videos helped students understand the concepts behind each neuronal pathway. The narrator used schematic illustrations of the brainstem at various levels, highlighting anatomical structures (e.g., the internal capsule) and explaining their relationships to other regions (e.g., the lentiform nucleus). The narrator also pointed to structures on the screen, sometimes highlighting them in different colors for emphasis.

Dynamic multimedia elements, such as animations and videos, are particularly useful for explaining functional neuroanatomy^63^.These concepts are difficult to study through cadaveric dissection or prosected specimens, but animations can simulate processes like sensory stimuli (e.g., pain or temperature), motor actions (e.g. leg flexion), and the flow of information along neuronal tracts. However, while incorporating multiple multimedia elements can enhance learning, it is important to be cautious. Overloading a resource with too many elements at once can overwhelm the learner and lead to cognitive overload^64,65^. Educators should use multimedia features thoughtfully and sparingly to maximize their effectiveness.

**Interpretation of the disparity between the identified strengths and weaknesses in the thematic analysis**

A notable disparity between the number of strengths and weaknesses identified in the included studies or the described resources was highlighted by this systematic review. Only four strengths were identified within the described resources whereas 12 weaknesses were identified within the resources or the studies describing them. This disparity perhaps reflects a combination of challenges associated with conducting high-quality pedagogical research, assessing educational studies and the lack of established guidelines for designing and evaluating multimedia resources for neuroanatomy education.

Remarkably, 22 out of the 29 resources were not accessible. It is possible that many of the neuroanatomy multimedia resources evaluated in this systematic review, could have been created to address immediate needs, without sufficient attention being given to their usability, pedagogical soundness or long-term sustainability. Also, some studies described the effectiveness of CAL resources (e.g., CompleteAnatomy, Anatomage) that were accessed by students once they were purchased by their institution. Hence, when resources come at a financial cost, they are less likely to be open access. On the other hand, immersive technologies such as VR and AR cannot be open access as they require specific equipment to operate therefore, limiting the number of users that can benefit from their use. Despite this, studies which describe such resources can provide readers with enough context on their creation, the neuroanatomical structures present in them and how the user interacts with them. This systematic review showed that various studies failed to adequately describe the neuroanatomy multimedia resource (N=8), how it was created (N=10) and the neuroanatomical structures it included (N=8), meaning that recreation of the resource by other neuroanatomy educators might not be feasible. These weaknesses identified in the included studies can be potentially attributed to differences in publishing standards. It is possible that earlier publications were subject to less stringent peer review which can potentially justify the limited information that was provided regarding a resource’s design, usability and content.

Other weaknesses identified across the included studies by this review included the absence of substantial information on the study’s methodology and result sections**.** Out of the 29 studies, five studies had inconsistencies in their results section and 14 studies did not describe adequately their methodology in terms of the design, structure, and content of the pre-test and/or post-test(s) they administered to students. An inference that can be made after the assessment of the 29 studies included for final analysis is that in seven studies, the information on the statistical analysis was problematic. Information on the type of tests that were used to determine statistical significance and correlation along with the corresponding *p* and *r* values and test scores and standard deviation values on either the pre-test or post-tests were omitted. Hence, the presence of such limitations within these articles limited the statistical evidence that this review could have provided. Such omissions along with other omissions such as flawed introduction, results, and discussion sections, are common across manuscripts that are being submitted in medical science and medical education journals^76,77^. Other important information that was often omitted in some studies included the method that was used to carry out randomization of the study participants, whether the allocation sequence was concealed from the participants and the study’s investigators, whether the study participants were blind to the study hypothesis and the intervention they were given or whether the individual analysing the study’s outcomes was blinded. When the aforementioned information was not provided in a study, the risk of bias assessment of this study could not be fully performed therefore, hindering the determination of the actual risk of bias present in a study. A solution to overcome these challenges, would be for peer reviewers and journal editors to request such information if omitted from submitted manuscripts and to provide authors with guidelines on how to write each section of a manuscript including how to comprehensively report their results. Recently, the Anatomical Sciences Education journal has published two discursive articles with journal specific recommended guidelines for survey -based research^78^, systematic reviews and meta-analyses^79^. These guidelines can be used by educators during the design process of a study and not solely during the writing process of a manuscript. Familiarity with such guidelines can help tackle poor study design planning, ensure greater methodological rigor, reproducibility and validity of the evidence reported. Moreover, these guidelines can be used by peer reviewers when reviewing manuscripts to ensure their critical appraisal, reduce subjective bias and help advance the quality of educational research.

This systematic review has identified four strengths within some of the resources described in the included studies which were a neuroanatomy multimedia resource exposing students to radiological imaging scans, containing a clinical application element, interactive features, and being designed upon evidence-based principles. The use radiological imaging scans and the presence of a clinical application element within a resource are a means of achieving vertical integration in neuroanatomy education which is one strategy that could help tackle neurophobia^23^. In vertical integration, pre-clinical knowledge is designed to be applied to a clinical context or vice versa^23^ . In terms of vertical integration within the medical curriculum, studies showed that when pre-clinical knowledge was applied into a clinical context by integrating case-based learning, case stimulated interactive lectures, clinical seminars as well as exposing students to patients, students expressed that their understanding was improved and their motivation towards neuroscience related topics was enhanced^24,80^. Furthermore, a study showed that vertical integration in neuroanatomy teaching resulted in students feeling more prepared once they entered the clinical years of their degree^24^ . Thus, a vertically integrated medical curriculum could assist students in applying their structural and functional neuroanatomy knowledge in the context of clinical neuroanatomy.

**Neuroanatomy resources designed upon evidence-based principles**

The design of a resource upon evidence-based principles and the incorporation of interactive features within it are means of enhancing learning outcomes and the students’ learning experience. Interestingly, only 10 out of the 29 studies included in the final analysis reported that the design of the resource they described was informed by evidence-based principles^40,43,47,51,52,55,58,67,70,73^. The design of these 10 neuroanatomy multimedia resources was informed by the principles of multimedia learning^40,43,47,51,52^ and the cognitive load theory^55,58,67,70,73^. Emphasis on how people learn is essential in medical education^63^. Understanding how learning takes place can assist in designing effective instruction. Applying the 12 principles of multimedia learning during the design and creation of a multimedia resource could create an effective multimedia learning experience for the students ^63^.

Only four out of the five studies that applied multimedia learning principles during the design of the resource, specified the principles that were used^40,47,58,51^. Allen et al. ^47^ specified that the design of their CAL resource was guided by the dual channel assumption and coherence effect principles. According to Paivio and Baddeley, visual and auditory information are processed by separate channels in humans^81,82^. The dual channel assumption is defined as humans possessing a visual channel that processes material that is represented visually or spatially, and an auditory channel that processes material that is auditorily or verbally presented^63^. The dual channel assumption underlines the cognitive theory of multimedia learning which is defined as the human brain’s limited capacity for processing information in the visual and auditory channels^83^. Selectively choosing the words and pictures that will be used during instruction is key as the incorporation of a multimedia element that does not directly relate to the material can negatively impact students’ learning^63^.

The coherence principle aims to reduce extraneous material that when present may hinder students’ learning. The coherence principle was used by Pickering et al. ^40^. Other principles that were used to reduce extraneous processing are the signalling and temporal and spatial contiguity principles which were used in three studies^40,51,58^. The signalling principle underlines that highlighting essential material via the use of headings, pointer words or use of an outline in a multimedia lesion can help people learn better ^83,84^. The spatial and temporal contiguity principles underscore that students learn better when corresponding words and pictures are presented close to each other and simultaneously instead of successively ^63^.

The segmentation principle which was applied by Javaid et al.^51^ aims to break large lessons into small learner-controlled segments^63^. Lastly, the redundancy principle, which was applied in three studies^40,51,58^ is the principle that aims to minimise the learner’s extraneous processing by avoiding the combined use of pictures and text when learners already possess sufficient knowledge^84^. When learners already possess prior knowledge in a subject, the use of one source only is sufficient as it enables them to build a mental model of what they are presented, hence, the use of a second source is deemed redundant ^84^.

As neuroanatomy is often a challenging subject due to the inherent complexity of neuroanatomical structures, it is essential that neuroanatomy educators take into consideration the various principles of multimedia learning and the cognitive load theory when designing and creating new neuroanatomy resources. The use of instructional design principles can ensure the creation of quality online learning resources. Equally important, is for neuroanatomy educators to understand student differences and how their content meets their students’ educational needs^51,85^.

**Limitations**

One limitation of this systematic review is that only three databases were used to identify relevant articles. This means that potentially eligible studies from other databases may have been overlooked. Additionally, during the initial screening, one non-English article was excluded, which could be considered another limitation. A further potential limitation is the use of the CASP checklist tool for assessing the risk of bias in the included studies. While the CASP tool is useful, it does not account for factors such as stakeholder opinions, result consistency, or publication bias, unlike the Grades of Recommendation, Assessment, Development, and Evaluation (GRADE) framework, which includes these elements when evaluating the quality of evidence^66^. A final limitation of this systematic review is that the strengths and weaknesses identified in the included studies during the thematic analysis stage were predominantly determined by the first author’s interpretation of each study’s results and based on information provided within the studies. This could have introduced subjective interpretation bias although efforts were made to ensure consistency through repeated reviews of the extracted data. The addition of at least one more rater for the identification of the strengths and weaknesses within the included studies could have perhaps strengthened the reliability of the findings.

**Future directions**

A neuroanatomy resource that focuses solely on structural neuroanatomy helps medical students develop foundational knowledge in lower-order cognitive skills, such as recognizing and localizing neuroanatomical structures. However, understanding structural neuroanatomy alone is insufficient for students to grasp how damage to specific structures leads to clinical symptoms. Therefore, future resources should also include a focus on functional neuroanatomy. This would help students develop higher-order cognitive skills, such as explaining the function of neuroanatomical structures and understanding how their spatial relationships and connections influence these functions. Furthermore, to enable students to apply both their factual (structural neuroanatomy) and conceptual (functional neuroanatomy) knowledge in real-world clinical contexts, resources should incorporate a clinical application component. By allowing students to connect their understanding of both structural and functional neuroanatomy to clinical scenarios, such resources can bridge the gap between basic neuroanatomy and clinical neurology, potentially reducing the prevalence of neurophobia.

**Conclusion**

This systematic review indicates that there is insufficient robust evidence to support the effectiveness of neuroanatomy multimedia resources in improving medical students’ understanding of neuroanatomy. The current evidence suggests that these resources primarily serve as supplementary learning tools. While they appear useful in enhancing students’ knowledge of structural neuroanatomy, they do not adequately address functional neuroanatomy.

The review highlights that 19 out of the 29 resources lack components related to functional neuroanatomy and clinical applications. As these resources focus exclusively on structural neuroanatomy, they fail to help students connect structure with function or apply this knowledge in clinical contexts. The remaining 10 resources primarily focus on structural neuroanatomy but include some functional neuroanatomy content, albeit limited. Only 10 of the 29 resources included a clinical application component, and the quality of this component varied significantly across the resources.

While cadaveric dissection and prosected specimens offer valuable tactile feedback for students manipulating human tissue, they are not ideal for teaching functional neuroanatomy, which largely occurs mainly at cellular and molecular levels. Additionally, this review found that many neuroanatomy multimedia resources lack important multimedia elements, such as sound, animations, and videos. Thoughtful incorporation of these dynamic elements could potentially lead to the development of high-quality resources that simplify complex neuroanatomical concepts. By enhancing students' understanding of structural and functional neuroanatomy, these resources could ultimately help bridge the gap between neuroanatomy knowledge and clinical symptomatology.

**Notes on Contributors**

ELENI PATERA B.Sc(Hons)., M.Sc., M.Sc., P.G.Cert.H.B.E, F.H.E.A. is a part-time PhD student and a Doctoral Academic Teacher at the Human Anatomy Resource Centre at the University of Liverpool, Liverpool, UK. She teaches gross systemic anatomy to graduate and undergraduate pre-clinical medical students, dentistry students and health-allied students. Her research interests include how students study and learn anatomy in their own time, as well as how anatomy educators can help students master their self-directed learning. Other research interests include the design of anatomy resources that help students develop lower-order and higher-order cognitive skills.

MARK PICKERING, B.A., PhD., is a lecturer in human anatomy at the School of Medicine at University College Dublin, in Dublin, Ireland. His teaching is primarily focused on preclinical neurosciences and embryology in both the undergraduate entry and graduate entry medicine programmes. His research interests are broadly focused on understanding the factors underpinning the structure and function of the nervous system and the application of simple fabrication methods such as 3D printing to development of low cost and flexible lab tools, such as microscopes

THOMAS C. FLANAGAN, B.Sc., Ph.D., is lecturer in human anatomy and principal investigator in the Discipline of Anatomy, School of Medicine at University College Dublin, in Dublin, Ireland. He coordinates the pre-clinical neurosciences course in the undergraduate medicine curriculum and teaches primarily neuroanatomy to undergraduate medicine, graduate-entry medicine, allied healthcare and engineering students. His research interests lie in the field of tissue engineering and medical devices, and in the development and deployment of e-learning technologies and customised anatomical models in anatomy education.

**Literature Cited**

[1] Neuwirth, L., Dacius, T. and Mukherji, B. (2018). Teaching Neuroanatomy Through a Historical Context. *Journal of Undergraduate Neuroscience Education*, 16(2), pp. E26-E31.

[2] Splittgerber, R. (2018). *Snell's Clinical Neuroanatomy*. 8th ed. Philadelphia, Pa: Wolters Kluwer, p.VII.

[3] Kramer, B. and Soley, J. T. (2002). Medical students perception of problem topics in anatomy. *East African Medical Journal*. 79(8), pp. 408-14. <https://doi.org/10.4314/eamj.v79i8.8826>.

[4] Javaid M. A, Chakraborty, S., Cryan J.F., Schellekens, H. and Toulouse, A. (2018). Understanding neurophobia: Reasons behind impaired understanding and learning of neuroanatomy in cross-disciplinary healthcare students. *Anatomical Sciences Education.* 11(1), pp.81-93 <https://doi.org/10.1002/ase.1711>

[5] Sotgiu, M. A., Mazzarello, V., Bandiera, P., Madeddu, R., Montella, A. and Moxham, B. (2020). Neuroanatomy, the Achille's Heel of Medical Students. A Systematic Analysis of Educational Strategies for the Teaching of Neuroanatomy. *Anatomical Sciences Education.* 13(1), pp.107-116. <https://doi.org/10.1002/ase.1866>

[6] Cheung, C. C., Bridges, S. M. and Tipoe, G. L. (2021). Why is Anatomy Difficult to Learn? The Implications for Undergraduate Medical Curricula. *Anatomical Sciences Education*. 14(6), pp. 752-763. <https://doi.org/10.1002/ase.2071>

[7] Mateen, F. and D’Eon, M. (2008). Neuroanatomy: a single institution study of knowledge loss. *Medical Teacher*, 30(5), pp.537-539. <https://doi.org/10.1080/01421590802064880>

[8] Hazelton, L. (2011). Changing concepts of neuroanatomy teaching in medical education. *Teaching and Learning in Medicine.* 23(4), pp. 359-64. <https://doi.org/10.1080/10401334.2011.611777>

[9] Singh, R., Yadav, N., Pandey, M. and Jones, D. G. (2022). Is inadequate anatomical knowledge on the part of physicians hazardous for successful clinical practice?. *Surgical and Radiologic Anatomy*, 44(1), pp.83-92. <https://doi.org/10.1007/s00276-021-02875-7>

[10] Menken, M. (2002). Demystifying neurology. *British Medical Journal*, 324(7352), pp.1469-1470. <https://doi.org/10.1136/bmj.324.7352.1469>

[11] Flanagan, E., Walsh, C. and Tubridy, N. (2007). 'Neurophobia'--attitudes of medical students and doctors in Ireland to neurological teaching. European Journal of Neurology, 14 (10), pp.1109-12. <https://doi.org/10.1111/j.1468-1331.2007.01911.x>

[12] McKeown, P. P., Heylings, D. J., Stevenson, M., McKelvey, K. J., Nixon, J. R. and McCluskey, D. R. (2003). The impact of curricular change on medical students' knowledge of anatomy. *Medical Education*. 37(11), pp.954-61. <https://doi.org/10.1046/j.1365-2923.2003.01670.x>.

[13] McBride, J. M. and Drake, R. L. (2018). National survey on anatomical sciences in medical education. *Anatomical Sciences Education*, 11(1), pp.7-14. <https://doi.org/10.1002/ase.1760>

[14] Jozefowicz, R. F. (1994). Neurophobia: the fear of neurology among medical students. *Archives of Neurology*. 51(4), pp. 328-329. <https://doi.org/10.1001/archneur.1994.00540160018003>

[15] Youssef, F. F. (2009). Neurophobia and its implications: evidence from a Caribbean medical school. *BMC Medical Education,* 9, pp.1-7, <https://doi.org/10.1186/1472-6920-9-39>

[16] Shelley, B. P., Chacko, T. V. and Nair, B. R. (2018). Preventing "Neurophobia": Remodeling Neurology Education for 21^st^-Century Medical Students through Effective Pedagogical Strategies for "Neurophilia". *Annals of Indian Academy of Neurology*. 21(1), pp. 9-18. <https://doi.org/10.4103/aian.AIAN_371_17>

[17] McCarron, M. (2012). A systematic review of neurophobia and perceived causes among medical students and junior doctors. *Journal of Neurology, Neurosurgery & Psychiatry,*83, p.e1. <http://dx.doi.org/10.1136/jnnp-2011-301993.75>

[18] Matthias, A. T., Nagasingha, P., Ranasinghe, P. and Gunatilake, S. B. (2013). Neurophobia among medical students and non-specialist doctors in Sri Lanka. *BMC Medical Education*. 13, pp.1-7. <https://doi.org/10.1186/1472-6920-13-164>

[19] Charalambous, N., Goh, A., Los, F. Sharma, K. (2015). Does Understanding Basic Neuroscience Cure Neurophobia?. *Journal of Neurology, Neurosurgery & Psychiatry,* 86(11), p.e4 <https://doi/org/10.1136/jnnp-2015-312379.5>

[20] Abushouk, A. I., Duc, N. M. (2016). Curing neurophobia in medical schools: evidence-based strategies. *Medical Education Online*. 21, p.32476. <https://doi.org/10.3402/meo.v21.32476>

[21] Hernando-Requejo, V. (2020). Neurophobia: why, how much, consequences and solutions [version 1]. *MedEdPublish* 2020,**9(1), pp.1-15,** <https://doi.org/10.15694/mep.2020.000003.1>

[22] Moreno-Zambrano, D., Sandrone, S., Meza-Venegas, J., Jimenez, J., Freire-Bonifacini, A., Santibanez-Vasquez, R. and Garcia-Santibanez, R. (2021). Exploring the key factors behind neurophobia: A systematic review of the English, Spanish and Portuguese literature. *Brain Disorders*, 2, pp.1-11. <https://doi.org/10.1016/j.dscb.2021.100011>

[23] Omar, N. A. (2021). Tackling Neurophobia among Medical Professionals from Its Inception: Role of Medical Curriculum to Treat This Epidemic Phenomenon. Education in Medicine Journal, 13*(1),* pp.73-85. <https://doi.org/10.21315/eimj2021.13.1.7>

[24] Haines, D. E., Hutchins, J.B. and Lynch, J. C. (2002). Medical neurobiology: Do we teach neurobiology in a format that is relevant to the clinical setting?. *The Anatomical Record*. 269(2), pp.99-106. <https://doi.org/10.1002/ar.10073>

[25] Nicolosi, F. *et al.* (2021) ‘New Neuroanatomy Learning Paradigms for the next generation of trainees: A novel literature-based 3D methodology’, *Clinical Neurology and Neurosurgery*, 210, p. 106948. <https://doi.org/10.1016/j.clineuro.2021.106948>

[26] Henelly, M. L. and Ctori, I. (2022). Technology in Education. Community Eye Health, 35(114), pp.22-23.

[27] Haleem, A., Javaid, M., Qadri, M. A. and Suman, R. (2022). Understanding the role of digital technologies in education: A review. Sustainable Operations and Computers, 3, pp.275-285. <https://doi.org/10.1016/j.susoc.2022.05.004>

[28] Ruiz, J. G., Mintzer, M. J. and Leipzig, R. M. (2006). The impact of e-learning in medical education. In *Academic Medicine*, 81(3), pp.207-212 <https://doi.org/10.1097/00001888-200603000-00002>

[29] McCoy, L., Lewis, J. H. and Dalton, D. (2016). Gamification and Multimedia for Medical Education: A Landscape Review. *The Journal of the American Osteopathic Association*, *116*(1), pp.22–34. <https://doi.org/10.7556/jaoa.2016.003>

[30] Mansour, L., Amin, S., Chirumamilla, V., Sushereba, C., Militello, L., Wolf, S., San Miguel, C., Barrie, M., Ramachandran, S. and Patterson, E. (2020). Exploring the Use of Multimedia Resources in Online Medical Education: A Targeted Literature Review. *Proceedings of the International Symposium on Human Factors and Ergonomics in Health Care*, 9(1), pp.201-205. <https://doi.org/10.1177/2327857920091028>

[31] Jean-François, R. (2008). Understanding Multimedia Documents. New York: Springer. Available at: <http://www1.udel.edu/fth/multilit/01-MultiLit.pdf> [Accessed June 6, 2022]

[32] Arantes, M., Arantes, J. and Ferreira, M. A. (2018). Tools and resources for neuroanatomy education: a systematic review. *BMC Medical Education*, *18*(1), p.94. <https://doi.org/10.1186/s12909-018-1210-6>

[33] Newman, H. J., Meyer, A. J., Wilkinson, T. J., Pather, N. and Carr, S. E. (2022). Technology enhanced neuroanatomy teaching techniques: A focused BEME systematic review of current evidence: BEME Guide No. 75. *Medical Teacher*, pp.1-12. <https://doi.org/10.1080/0142159X.2022.2039382>

[34] Chytas, D., Piagkou, M., Salmas, M. and Johnson, E. O. (2020). Is Cadaveric Dissection The “Gold Standard” For Neuroanatomy Education?. *Anatomical Sciences Education*., 13(6), pp.804-805.  <https://doi.org/10.1002/ase.1957>

[35] Moher, D., Shamseer, L., Clarke, M., Ghersi, D., Liberati, A., Petticrew, M., Shekelle, P., Stewart, L. A. and PRISMA-P Group. Preferred reporting items for systematic review and meta-analysis protocols (PRISMA-P) 2015 statement. *Syst Rev* **4**, 1 (2015). <https://doi.org/10.1186/2046-4053-4-1>

[36] Eriksen MB, Frandsen TF. The impact of patient, intervention,
comparison, outcome (PICO) as a search strategy tool on literature search quality: a systematic review. J Med Libr Assoc.
2018;106(4):420–31

[37] Randomised Controlled Trials (RCTS) checklist (2024) *CASP*. Available at: https://casp-uk.net/casp-tools-checklists/randomised-controlled-trial-rct-checklist/ (Accessed: 07 October 2024).

[38] Svirko, E. and Mellanby, J. (2008). Attitudes to e-learning, learning style and achievement in learning neuroanatomy by medical students. *Medical Teacher*, *30*(9-10), pp.e219-e227. <https://doi.org/10.1080/01421590802334275>

[39] Brewer, D., Wilson, T., Eagleson, R. and de Ribaupierre, S. (2012). Evaluation of Neuroanatomical Training using a 3D Visual Reality Model. *Studies In Health Technology And Informatics*, *173*, pp.85-91. <https://doi.org/10.3233/978-1-61499-022-2-85>

[40] Pickering, J. D., Panagiotis, A., Ntakakis, G., Athanassiou, A., Babatsikos, E. and Bamidis, P. D. (2022). Assessing the difference in learning gain between a mixed reality application and drawing screencasts in neuroanatomy. *Anatomical Sciences Education*, *15*(3), pp. 628-635. <https://doi.org/10.1002/ase.2113>

[41] de Faria, J., Teixeira, M., de Moura Sousa Júnior, L., Otoch, J. and Figueiredo, E. (2016). Virtual and stereoscopic anatomy: when virtual reality meets medical education. *Journal Of Neurosurgery*, *125*(5), pp.1105-1111. <https://doi.org/10.3171/2015.8.jns141563>

[42] Lamperti, A. and Sodicoff, M. (1997). Computer-based neuroanatomy laboratory for medical students. *The Anatomical Record*, *249*(3), pp.422-428. [https://doi.org/10.1002/(sici)1097-0185(199711)249:3<422::aid-ar14>3.0.co;2-q](https://doi.org/10.1002/(sici)1097-0185(199711)249:3%3c422::aid-ar14%3e3.0.co;2-q)

[43] Allen, E., Walls, R. and Reilly, F. (2008). Effects of interactive instructional techniques in a web-based peripheral nervous system component for human anatomy. *Medical Teacher*, *30*(1), pp.40-47. <https://doi.org/10.1080/01421590701753518>

[44] Lewis, E., Strike, M., Doja, A., Ni, A., Weber, J., Wiper-Bergeron, N. and Sell, E. (2011). Web-Based Software to Assist in the Localization of Neuroanatomical Lesions. *Canadian Journal Of Neurological Sciences,* *38*(2), pp.251-255. <https://doi.org/10.1017/s0317167100011422>

[45] Ruisoto Palomera, P., Juanes Méndez, J. and Prats Galino, A. (2014). Enhancing neuroanatomy education using computer-based instructional material. *Computers In Human Behavior*, *31*, pp.446-452. <https://doi.org/10.1016/j.chb.2013.03.005>

[46] Drapkin, Z., Lindgren, K., Lopez, M. and Stabio, M. (2015). Development and assessment of a new 3D neuroanatomy teaching tool for MRI training. *Anatomical Sciences Education*, *8*(6), pp.502-509. <https://doi.org/10.1002/ase.1509>

[47] Allen, L., Eagleson, R. and de Ribaupierre, S. (2016). Evaluation of an online three-dimensional interactive resource for undergraduate neuroanatomy education. *Anatomical Sciences Education*, *9*(5), pp.431-439. <https://doi.org/10.1002/ase.1604>

[48] Peterson, D. and Mlynarczyk, G. (2016). Analysis of traditional versus three-dimensional augmented curriculum on anatomical learning outcome measures. *Anatomical Sciences Education*, *9*(6), pp.529-536. <https://doi.org/10.1002/ase.1612>

[49] Svirko, E. and Mellanby, J. (2017). Teaching neuroanatomy using computer-aided learning: What makes for successful outcomes?. *Anatomical Sciences Education*, *10*(6), pp. 560-569. <https://doi.org/10.1002/ase.1694>

[50] Welch, M. C., Yu, J., Larkin, M. B., Graves, E. K. and Mears, D. (2020). A Multimedia Educational Module for Teaching Early Medical Neuroanatomy. *MedEdPORTAL: the journal of teaching and learning resources*, *16*, p.10885. <https://doi.org/10.15766/mep_2374-8265.10885>

[51] Javaid, M. A., Schellekens, H., Cryan, J. F. and Toulouse, A. (2020b). Neuroanatomy of the spinal pathways: Evaluation of an interactive multimedia e-learning resource [version 1]. MedEdPublish*.* 9:88. <https://doi.org/10.15694/mep.2020.000088.1>

[52] van Walsum, A. and Henssen, D. (2022). E‐Learning Three‐Dimensional Anatomy of the Brainstem: Impact of Different Microscopy Techniques and Spatial Ability. *Anatomical Sciences Education*, *15*(2), pp.317-329. <https://doi.org/10.1002/ase.2056>

[53] Kockro, R. A, Amaxopoulou, C., Killeen, T., Wagner, W., Reisch, R., Schwandt, E., Gutenberg, A., Giese, A., Stofft, E. and Stadie, A. T. (2015). Stereoscopic neuroanatomy lectures using a three-dimensional virtual reality environment. *Annals Of Anatomy*, 201, pp.91-98. <https://doi.org/10.1016/j.aanat.2015.05.006>

[54] Bernard, F., Richard, P., Kahn, A. and Fournier, H. (2020). Does 3D stereoscopy support anatomical education?. *Surgical And Radiologic Anatomy*, *42*(7), pp.843-852. <https://doi.org/10.1007/s00276-020-02465-z>

[55] Henssen, D., van den Heuvel, L., De Jong, G., Vorstenbosch, M., van Cappellen van Walsum, A. M., Van den Hurk, M. M., Kooloos, J. and Bartels, R. (2020). Neuroanatomy Learning: Augmented Reality vs. Cross-Sections. *Anatomical sciences education*, *13*(3), pp.353–365. <https://doi.org/10.1002/ase.1912>

[56] Stepan, K., Zeiger, J., Hanchuk, S., Del Signore, A., Shrivastava, R., Govindaraj, S. and Iloreta, A. (2017). Immersive virtual reality as a teaching tool for neuroanatomy. *International Forum Of Allergy & Rhinology*, *7*(10), pp.1006-1013. <https://doi.org/10.1002/alr.21986>

[57] Ekstrand, C., Jamal, A., Nguyen, R., Kudryk, A., Mann, J. and Mendez, I. (2018). Immersive and interactive virtual reality to improve learning and retention of neuroanatomy in medical students: a randomized controlled study. *CMAJ Open*, *6*(1), pp. E103-E109. <https://doi.org/10.9778/cmajo.20170110>

[58] Küçük, S., Kapakin, S. and Göktaş, Y. (2016). Learning anatomy via mobile augmented reality: Effects on achievement and cognitive load. *Anatomical Sciences Education*, *9*(5), pp.411-421. <https://doi.org/10.1002/ase.1603>

[59] Tam, M.D., Hart, A. R., Williams, S., Heylings, D. and Leinster, S. (2009). Is learning anatomy facilitated by computer-aided learning? A review of the literature. *Medical teacher*, *31*(9), pp.e393–e396. <https://doi.org/10.1080/01421590802650092>

[60] Bogomolova, K., Hierck, B. P., Looijen, A. E. M., Pilon, J. N. M., Putter, H., Wainman, B., Hovius, S. E. R. and van der Hage, J. A. (2021). Stereoscopic three-dimensional visualisation technology in anatomy learning: A meta-analysis. *Medical Educ*ation. 55, pp. 322– 332. <https://doi.org/10.1111/medu.14352>

[61] Jacquesson, T., Simon, E., Dauleac, C., Margueron, L., Robinson, P. and Mertens, P. (2020). Stereoscopic three-dimensional visualization: interest for neuroanatomy teaching in medical school. *Surgical And Radiologic Anatomy*, 42(6), pp.719-727. <https://doi.org/10.1007/s00276-020-02442-6>

[62] Diamond, M. (2011). The role of narrative in multimedia learning. Doctor of Philosophy in Curriculum and Instruction. *University of Nevada, Las Vegas*.

[63] Mayer, R. E. (2010). Applying the science of learning to medical education. *Medical Education*, 44(6), pp.543-549. <https://doi.org/10.1111/j.1365-2923.2010.03624.x>

[64] Mayer, R. E. (2008). Applying the science of learning: Evidence-based principles for the design of multimedia instruction. American Psychologist, 63(8), pp.760–769. [https://doi.org/10.1037/0003-066X.63.8.760](https://psycnet.apa.org/doi/10.1037/0003-066X.63.8.760)

[65] Sweller, J., van Merriënboer, J. J. G. and Paas, F. (2019). Cognitive Architecture and Instructional Design: 20 Years Later. *Educational Psychology Review,* 31, pp.261–292 <https://doi.org/10.1007/s10648-019-09465-5>

[66] Purssell, E. (2020). Can the Critical Appraisal Skills Programme check-lists be used alongside Grading of Recommendations Assessment, Development and Evaluation to improve transparency and decision-making? *Journal of Advanced Nursing,* 76(4), pp. 1082-1089 <https://doi.org/10.1111/jan.14303>

[67] Booker, J., Woodward, C., Taylor, C., Robson, A. and Border, S. 2023. Creating evidence‐based engaging online learning resources in neuroanatomy. Anatomical Sciences Education, 17(3), pp.605–619.

[68] Xuan, H., Zhong, J., Wang, X., Song, Y., Shen, R., Liu, Y., Zhang, S., Cai, J. and Liu, M. (2024). GRAVEN: a database of teaching method that applies gestures to represent the neurosurgical approach’s blood vessels and nerves. BMC Medical Education, 24(509), 1-9. https://doi.org/10.1186/s12909-024-05512-0

‌[69] Yun, Y.H., Kwon, H.Y., Jeon, S.K., Jon, Y.M., Park, M.J., Shin, D.H., Choi, H.J. (2024). Effectiveness and satisfaction with virtual and donor dissections: A randomized controlled trial. *Sci Rep* **14**, 16388. <https://doi.org/10.1038/s41598-024-66292-7>

[70] Yohannan, D.G., Oommen, A.M., Kumar, A.S. Devanand, S., UT, M.R., Sajan, N., Thomas, N.E., Anzer, N., Raju, N.K., Thomas, B., Rajan, J.E., Govindapillai, U.K., Harish, P., Kapilamoorthy, T.R., Kesavadas, C. and Sivaswamy, J. (2024). “Visualization matters” – stereoscopic visualization of 3D graphic neuroanatomic models through *AnaVu* enhances basic recall and radiologic anatomy learning when compared with monoscopy. *BMC Med Educ* **24**, 932. <https://doi.org/10.1186/s12909-024-05910-4>

[71]T, Richard. and R, Rajakumari. (2023). Enhancing Medical Students' Anatomy Memory through Virtual Reality Dissection Simulations.  *ICSES*, pp. 1-8, https://doi.org/10.1109/ICSES60034.2023.10465581

[72] Cercenelli, L., Stradiotti, S., Bortolani, B., Tarsitano, A., Manzoli, L.,.(2024). AEducAR3.0: An Exciting Hybrid Educational Platform for a Comprehensive Neuroanatomy Learning. In: De Paolis, L.T., Arpaia, P., Sacco, M. (eds) Extended Reality. XR Salento 2024. Lecture Notes in Computer Science, vol 15028. Springer, Cham. https://doi.org/10.1007/978-3-031-71704-8_10

[73]Zeedzen-Scheffers, I., Karstens, J., van den Hurk, M., Henssen, D. and Boer, L.L. (2024). Comparing the effectiveness of augmented reality and anatomical atlases in student preparation for neuroanatomy dissection. *Sci Rep* **14**, 24939 (2024). https://doi.org/10.1038/s41598-024-76379-w

[74] Gurses, M.E., Gökalp, E., Gecici, N.N., Gungor, A., Berker, M., Ivan, M.E., Komotar, R.J., Cohen-Gadol, A.A., Türe, U. (2024). Creating a neuroanatomy education model with augmented reality and virtual reality simulations of white matter tracts. J Neurosurg.141(3):865-874. <https://doi.org/10.3171/2024.2.JNS2486>

[75] Salimi, S., Asgari, Z., Mohammadnejad, A., Teimazi, A., Bakhtiari, M. (2024). Efficacy of virtual reality and augmented reality in anatomy education: A systematic review and meta‐analysis. Anatomical Sciences Education. [**https://doi.org/10.1002/ase.2501**](https://doi.org/10.1002/ase.2501)

‌[76] Ezeala, C., Nweke, I., Ezeala, M. (2013). Common errors in manuscripts submitted to medical science journals. Annals of Medical and Health Sciences Research. 3(3):376.

[77] Gottlieb, M., Dehon, E., Jordan, J., Bentley, S., Ranney, M. L., Lee, S., Khandelwal, S. and Santen, S. A. (2018). Getting Published in Medical Education: Overcoming Barriers to Scholarly Production. *The western journal of emergency medicine*, 19(1), pp.1–6. <https://doi.org/10.5811/westjem.2017.11.35253>

[78] Wilson, A.B., Bay, B.H., Byram, J.N., Carroll, M.A., Finn, G.M., Hammer, N., Hildebrandt, S., Krebs, C., Wisco, J.J., Organ, J.M. Journal recommended guidelines for survey-based research. (2024) Anatomical Sciences Education.17(7):1389-1391. <https://doi.org/10.1002/ase.2499>

[79] Wilson, A.B., Bay, B.H., Byram, J.N., Carroll, M.A., Finn, G.M., Hammer, N., Hildebrandt, S., Krebs, C., Wisco, J.J., Organ, J.M. (2024). Journal recommended guidelines for systematic review and meta‐analyses. Anatomical Sciences Education. 17(7):1392–5. [**https://doi.org/10.1002/ase.2500**](https://doi.org/10.1002/ase.2500)

**[80]** Ghosh, S. and Pandya, H.V. (2008). Implementation of Integrated Learning Program in neurosciences during first year of traditional medical course: Perception of students and faculty. *BMC Medical Education* 8, pp.1-8 <https://doi.org/10.1186/1472-6920-8-44>

‌[81] Paivio, A. (1990). Mental Representations: A Dual-Coding Approach. Oxford Psychology Series (New York, 1990; online edn, Oxford Academic, 1 Sep 2008), <https://doi.org/10.1093/acprof:oso/9780195066661.001.0001>

[82] Baddeley, A. D. (1999). Essentials of Human Memory. Boston, MA: Allyn & Bacon 1999.

[83] Mayer, R. E. (2005). Cognitive Theory of Multimedia Learning. In R. E. Mayer (Ed.), The Cambridge handbook of multimedia learning (pp. 31–48). Cambridge University Press. [https://doi.org/10.1017/CBO9780511816819.004](https://psycnet.apa.org/doi/10.1017/CBO9780511816819.004)

[84] Mayer, R. E. (2014). Incorporating motivation into multimedia learning. *Learning and Instruction*, 24, pp.171–173. <https://doi.org/10.1016/j.learninstruc.2013.04.003>

McBride, J. M. and Drake, R. L. (2018). National survey on anatomical sciences in medical education. *Anatomical Sciences Education*, 11(1), pp.7-14. <https://doi.org/10.1002/ase.1760>

[85] Felder, R. and Brent, R. (2005). Understanding Student Differences. *Journal of Engineering Education*, 94(1), pp.57-72. <https://doi.org/10.1002/j.2168-9830.2005.tb00829.x>

**Figure legends:**

**Figure 1:** Flow chart of article selection summary according to PRISMA-P guidelines (Moher et al., 2015, Syst. Rev.).

**Figure 2:** A donut chart indicating the number of articles describing each of the 5 types of multimedia resources that were identified after the 29 articles included in the final analysis were assessed. There was one study that described a resource that fitted into more than one type of a multimedia resource category (Augmented Reality and Virtual Reality),however, the resource that was utilized by medical students was an augmented reality resource whereas the virtual reality resource was utilized by neurosurgery residents, hence, the augmented reality resource was categorized under the ‘Augmented Reality’ category only.

**Figure 3:** Colored bar charts illustrating the multimedia elements present the 16 Computer-Assisted Learning (CAL), four stereoscopic, three virtual reality (VR), five augmented reality (AR) and mixed reality (MR) resources.

**Table 1:** Inclusion and exclusion criteria for each domain of PICO(s) and other eligibility variables in relation to the research question.

| **PICO(s)** | **Inclusion** | **Exclusion** |
| --- | --- | --- |
| **Population** | • Undergraduate or graduate pre-clinical medical students regardless of country of education or participants’ age | • Clinical undergraduate or graduate medical students  • Undergraduate or postgraduate students studying any other major other than medicine  • Junior doctors  • Neurology/neurosurgery residents/trainees/physicians |
| **Exposure** | • Neuroanatomy multimedia resources (varying from immersive technologies [VR, AR, mAR, MR, stereoscopy] to computer assisted learning (CAL) resources)) | • Digital resources without the use of multimedia  • Digital resources with extremely limited use of multimedia elements  • Gross anatomy multimedia resources  • Microanatomy/Histology multimedia resources |
| **Comparator** | • Immersive technology other than the one used as the exposure (VR, AR, mAR, MR, stereoscopy)  • CAL resources  • However, no studies were excluded if a comparator was not used | • N/A |
| **Outcome** | • Any educational neuroanatomy multimedia resource that was specifically designed for pre-clinical medical students and evaluated the effectiveness of the resource in improving pre-clinical medical students’ understanding of neuroanatomy | • Any “physical” model (i.e., 3D printed model)  • Any multimedia model that relates to microanatomy/histology  • Dissection tools  • Any multimedia model that was not designed specifically for pre-clinical medical students |
| **Study design** | • Any study design was acceptable as long as it addressed the systematic review’s research question and any other additional research questions. | • N/A |
| **Publication type** | • Journal Research Articles  • Book chapters | • Research Articles behind a paywall  • Case reports  • Viewpoint commentaries  • Book chapters behind a paywall  • Letters to the Editor  • Systematic reviews and Meta-Analyses |
| **Language** | • English | • Any language other than English |
| **Year of publication** | • April 1994- March 2022 | • Articles published before April 1994 |

AR: Augmented Reality

CAL: Computer-Assisted-Learning

mAR: mobile Augmented Reality

MR: Mixed reality

N/A: Not applicable

3D: Three-dimensional

PICO(s): Patient/Population, Intervention, Comparison and Outcomes and Study.

VR: Virtual Reality

**Table 2:** Study characteristics of included studies

| **Study (Authors, Year of Publication, Country, Reference Number)** | **Study Design** | **Participants** | **Exposure/ Intervention** | **Control Intervention/ Comparator** | **Study Aims** | **Outcomes regarding impact on students’ understanding of neuroanatomy** |
| --- | --- | --- | --- | --- | --- | --- |
| **Computer-Assisted Learning (CAL) Resources** | | | | | | |
| Lamperti and Sodicoff, 1997 (United States) [42] | Not specified | First year medical students (N=185) | CAL program consisting of a computerized atlas, a laboratory guide and a clinical problem-solving section | N/A | Development of a CAL resource to replace the traditional glass-slide/microscope-based laboratory component of a course and compare student performance between students who received the traditional program versus the CAL program | • Students who were trained via the CAL resource scored significantly higher (p<0.0001) than students who were taught by the traditional method. |
| Allen et al. 2008 (United States) [43] | Not specified | First year medical students (N=856) | Treatment 1:  Didactic lecture & dissection lab for years 1995-1997  Treatment 2:  Didactic lecture & dissection lab & PowerPoint lecture notes on CD video game console for year 2000  Treatment 3:  Didactic lecture & dissection lab & web-based lecture notes, & web- based interactive learning objects (patient case studies, review games, stimulated interactive patients, flashcards, and quizzes) for years 2001-2005 | N/A | To determine the effect of web-based interactive instructional techniques on students’ written exam performance and students’ opinions on elements of the web-based resource | • A statistically significant difference was observed between treatments 1 and 3 but not between treatments 1 and 2 or treatments 2 and 3.  • Students who received treatment 3 correctly answered a higher number of questions compared to students who received any of the other two types of treatment. |
| Svirko and Mellanby, 2008 (United Kingdom) [38] | Not specified | Second year medical students (N=205) | Neuroanatomy CAL course | N/A | To evaluate how successful a neuroanatomy CAL course was at encouraging students to employ a deep approach to learning | • Students reported higher surface approach scores and lower deep approach scores for the CAL course than for their studies in general. |
| Lewis et al. 2011 (Canada) [44] | Randomized Controlled Study | Second year medical students (N=39) | Web-based neuroanatomy localization application | Two neuroanatomy textbook resources | To evaluate the educational effectiveness of a web-based neuroanatomy localization application | • Student scores for the MCQ test were 67.5% and 54.7% for the intervention and control group, respectively.  • The intervention group showed a significantly higher (p=0.028) mean raw score than the control group. |
| Brewer et al. 2012 (Canada) [39] | Randomized Controlled Study | Second year medical students (N=118) & health science students (N=13) | Health science students:  Group 2/ 2D Group: Pre-Digital Lab or Group 3/3D Group: 3D Pre- Digital Lab  Medical students:  3D Pre- Digital Lab | Health science students:  Control group  Medical students:  No comparator as they experienced both the digital lab and gross lab but in a different order. | To evaluate the effectiveness of a CAL digital lab resource in improving the participants’ knowledge of neuroanatomy post-lecture and either pre or post the gross lab practical | Health science students:  • No statistically significant differences were found between the three groups neither between the two experimental groups. Post-test scores of students in group 3 were the highest from all three groups.  Medical students:  • There were no statistically significant differences between the two groups. |
| Ruisoto Palomera et al. 2014 (Spain) [45] | Not specified | Medical students (N=65)  *Year of study was not specified | Computer-Based Tool | N/A | To develop a computer-based tool to explore neuroanatomy based on three- dimensional images and to compare whether the educational value assigned by students varies according to their visuospatial ability | • No statistically significant differences were observed between students who showed high visuospatial ability and students who showed low visuospatial ability in any of the four items of the survey (p>0.01). |
| Drapkin et al. 2015 (United States) [46] | Randomized Controlled Study | First year medical students (N=62) | Neuroanatomy lecture with 3D neuroanatomy teaching tool (N=33) | Neuroanatomy lecture with traditional methods  (N=29) | To develop a computerized (3D) neuroanatomy teaching tool for training medical students to identify subcortical structures on a magnetic resonance imaging (MRI) series of the human brain and to assess its efficacy. | • There were no statistically significant differences between the MRI identification scores across the experimental and control groups.  • A statistically significant difference (p<0.01) was observed for questions that involved C-shaped structures as the experimental group outperformed the control group by an estimated difference in average score of 15.8%. |
| Allen et al. 2016 (Canada) [47] | Randomized Crossover Study | Second year medical students (N=47) | Group A:  Access to the online 3D learning module and subsequently had access to the cadaveric laboratory session  Group B:  Access to the cadaveric laboratory session and subsequently had access to the online 3D learning resource | N/A | To examine the educational efficacy of a newly developed 3D neuroanatomy module | • The only statistically significant difference between Groups A and B was observed for the post-test scores as it was shown that students who accessed the 3D learning module before accessing the cadaveric laboratory session performed significantly better on this test (p<0.01).  • Statistical analysis of scores within each group revealed that students in both groups scored significantly higher on the post-test than on the pre-test (p<0.01). |
| Peterson and Mlynarczyk, 2016 (United States) [48] | Not specified | Graduate medical students (N=51)  Upper-level undergraduate medical students (N=5)  Total number of students: N=56  *Year of study was not specified | Traditional teaching material augmented with computerized 3D teaching tools | Traditional teaching material | To analyse the examination performance of students on questions covering material taught with traditional learning formats versus material covered with traditional formats augmented with digital 3D technology. | • Student performance for questions covering material taught with 3D teaching tools was statistically significantly higher (p<0.0001) than for questions covering material taught with traditional teaching methods.  • Students performed significantly better (p<0.0001) on cadaveric SPOT exam questions that were taught with 3D teaching tools than for cadaveric SPOT exam questions taught with traditional teaching methods. |
| Svirko and Mellanby, 2017 (United Kingdom) [49] | Not specified | Second year medical students (N=869) | Neuroanatomy CAL course | Traditional neuroanatomy course | To compare the approach to learning that the students adopted toward a neuroanatomy CAL course with their approach to the more traditional aspects of their neuroanatomy instruction and examine whether students’ approach to learning related to their performance in tests and examinations | • Students’ deep approach scores were significantly lower for the CAL course than for the traditional neuroanatomy course (p<0.001). |
| Welch et al. 2020 (United States) [50] | Not specified | First year medical students  (N=314) | Neuroanatomy multimedia learning module | N/A | To assess the effectiveness of a neuroanatomy multimedia learning module that was developed and was offered as optional study aids to pre-clinical medical students | • No statistically significant differences were observed in the study conducted by Welch et al. (2020). |
| Javaid et al. 2020b (Ireland) [51] | Single-blinded controlled study | 1^st^ year graduate medical students (N=34), second year undergraduate medical students (N=46) & third-year undergraduate clinical therapy students (N=5) | Experimental Group [N=28]:  Interactive CAL resource on spinal pathways developed by Javaid et al.  No-use Group [N=36]:  No use of the interactive CAL resource developed by Javaid et al., neither use of the Functional Neuroanatomy resource developed by Krebs et al. | Control Group [N=21]:  Functional Neuroanatomy resource developed by Krebs et al. |  | • No statistically significant differences were observed for participants in each of the three groups regarding their percentage of correct answers in the pre- or the post-test in relation to the questions’ level of difficulty.  • For all three groups, there was a statistically significant difference in students’ neuroanatomy knowledge on the spinal pathways between the pre- and post-tests for the easy and difficult questions and total scores overall.  • Students in all three groups performed statistically significantly better in the post-test.  • No statistically significant difference was noted across students’ median normalized learning gain scores.  • A statistically significant difference was observed for the median normalized learning gains of participants in the no-use group for the easy versus difficult questions (p<0.001). |
| Van Walsum Cappellen and Hennssen, 2022 (Netherlands) [52] | Randomized Controlled Study | Second year medical students (N=38) | Group 1:  Students were exposed to  E-Learning Modules 1, 2 and 4  (N=19)  Group 2:  Students were exposed to  E-Learning Modules 1, 3 and 4  (N=19) | N/A | To evaluate the effect of spatial ability on cross-sectional e-learning brainstem anatomy and examine the learning outcomes of students working with PLI images and learning outcomes of students working with LFB images | • No statistically significant differences were observed between the MRT scores of male and female medical students (p=0.134).  • No statistically significant differences were observed between the pre-intervention, post-intervention and long-term test scores of the two groups.  • Post-intervention and long-term anatomical test scores were significantly higher than pre-intervention test scores for both groups indicating improvement irrespective of the e-learning module students were assigned. |
| Booker et al. 2024  (United Kingdom)  [67] | Cross-sectional study | Second year medical students (N=76) | Online video resources from Soton Brain Hub focusing on ‘pain pathways’ (N=53) | Paper copy of a text-based resource focusing on ‘pain pathways’ (N=23) | To compare the effectiveness of text-based resources and online video learning resources from the Soton Brain Hub online educational platform. | • Students who accessed the Soton Brain Hub video resources showed significantly higher immediate learning gains than students who accessed the text-based resources (p=0.030).  • No statistically significant differences were observed for the retained learning gains of the two groups (p=0.919). |
| Xuan et al. 2024 (China) [68] | Randomized Controlled Study | Undergraduate medical students  (N=50)  *Year of study was not specified | GRAVEN database:  Access to GRAVEN CAL resource and traditional neuroanatomy teaching methods | Control group:  Students received traditional teaching methods (PowerPoint teaching, Rhoton’s Cranial Anatomy and Surgical Approaches, Neurosurgery Tricks of the Trade (N=25) | To develop the GRAVEN database to help students learn neuroanatomy better and evaluate whether access to both the database and traditional neuroanatomy teaching methods improves academic performance. | • Students who had access to traditional neuroanatomy teaching methods and the GRAVEN CAL database scored statistically significantly higher in the test than students who had access to traditional neuroanatomy teaching methods only (p=0.0026). |
| Yun et al. 2024 (Republic of Korea) [69] | Randomized Control Study | First year medical students (N=154) | Virtual dissection group (N=71):  Accessed the Complete Anatomy app on a tablet to study structures of the diencephalon, telencephalon and structures associated with the third and lateral ventricles. | Donor group (N=83):  Accessed donor dissections which were prepared in advance by tutors. | To investigate medical students’ academic performance and satisfaction when utilizing virtual vs donor dissections. | • The mean score of students in the virtual group for Quiz 1 was statistically significantly higher than that in the donor group (p<0.05)  • No statistically significant differences were observed between the mean scores of Quiz 2 between the two groups. |
| **Stereoscopic Resources** | | | | | | |
| Köckro et al. 2015 (Germany) [53] | Randomized Controlled Study | Second year medical students (N=169) | 3D Group:  Pre-recorded audio lecture on the third ventricle & 3D animated tour of the third ventricle with DextroBeam  (N=89) | Control Group/2D Group:  Pre-recorded audio lecture on the third ventricle & 2D PowerPoint Presentation (N=80) | To evaluate the efficacy of a stereoscopically presented 3D neuroanatomical model to a large group of students and assess students’ retention of anatomical knowledge | • Mean score for the MCQ test was higher for the 3D group (5.45) than the 2D Group (5.19), however, there was no statistically significant differences between the two groups.  • Due to absence of a large sample size (N=169; 3D group: N=89, 2D group: N=20), the superiority of the 3D teaching over the 2D teaching cannot be determined accurately. |
| de Faria et al. 2016 (Brazil) [41] | Randomized Controlled Study | Graduate medical students (N=84)  *Year of study was not specified | Group 1: Received a traditional lecture exhibiting 2D images  Group 2:  Received a lecture that used interactive non-stereoscopic methods  Group 3:  Received a lecture that used interactive stereoscopic methods to demonstrate 1 non-stereoscopic and 1 stereoscopic video | N/A | To develop and evaluate a virtual stereoscopic resource for neuroanatomy teaching | • Students in groups 2 and 3 scored significantly higher than students in group 1 in the post-test scores for their written theory exam (p<0.05)  • Comparison of the mean values of the pre and post tests showed that there was a statistically significant improvement in the post-test scores of groups 2 and 3.  • In terms of scores of the practical exam, there was a statistically significant difference in the mean scores between group 1 and groups 2 and 3. |
| Bernard et al. 2020 (France) [54] | Prospective Randomized Controlled Study | Second year medical students (N=175) | 3D Group: 3D stereoscopic video (N=91) | 2D Group: Non- stereoscopic video (N=84) | To investigate whether a 3D stereoscopic instruction video on the Circle of Willis could improve learning over a 2D video. | • There was a statistically significant difference between the scores of the two groups as students from the 3D group scored higher than students from the 2D group in the anatomical relations and clinical reasoning sections (p=0.01) |
| Yohannan et al. 2024 (India) [70] | Three-limb Randomized Controlled Study | First-year medical students (N=152) | Stereoscopic group:  Students received a 20-minute demonstration on the brainstem lesson module via AnaVu in stereoscopic mode.  Monoscopic group:  Students received a 20-minute demonstration on the brainstem lesson module via AnaVu in monoscopic mode. | Control group:  Students were taught neuroanatomy via white board drawn diagrams. | To assess the utility of the AnaVu tool compared to conventional methods. | • All three groups showed statistically significant improvement from pre- to post-test (p < 0.001).  • The Stereo group scored significantly higher than both the Mono (p = 0.03) and Control groups (p = 0.001) in basic recall questions, and both the Stereo and Mono groups outperformed the Control group in radiology-based questions (p < 0.001 and p = 0.046, respectively). |
| **Virtual Reality (VR) Resources** | | | | | | |
| Stepan et al. 2017 (United States) [56] | Randomized Controlled Study | First year medical students (N=34) & second year medical students (N=32) | VR Group:  VR model of brain anatomy; students had a 10-minute VR study comprised of a 5-minute 3D video showing key anatomic relationships & 5 minutes of a fully immersive VR experience, and a 10-minute session with control study materials | Control Group:  20-minute independent study with control study materials  *Study control materials were not defined by the authors | To evaluate the effectiveness, satisfaction, and motivation associated with immersive VR simulation in teaching medical students neuroanatomy | • No statistically significantly differences were observed between the VR and Control groups for their scores on the pre-intervention, post-intervention, or retention quizzes.  • Second year medical students scored significantly higher than first year medical students in all three quizzes (p< 0.01). |
| Ekstrand et al. 2018 (Canada) [57] | Randomized Controlled Study | First year medical students (N=41) & second year medical students (N=23) | VR Group:  (N=31)  Virtual reality learning material illustrating a set of brains structures.  Virtual reality brain was presented via a headset using two handheld remotes; participants had 12 minutes of study time to memorize spatial relationships | Paper-based Group:  (N=33) Booklet containing 15 colored figures and corresponding labels for the same set of brain structures presented in the VR group. Colored figures were obtained from the Blumenfeld’s Neuroanatomy Through Clinical Cases, textbook; participants had 12 minutes of study time to memorize spatial relationships | To examine the impact of immersive virtual-reality neuroanatomy training and compare it to traditional paper-based methods | • No statistically significant differences were observed between student scores from the two groups for the pre and two post-intervention tests. |
| Richard and Rajakumari, 2023 (India) [71] | Randomized Controlled Study | First- and second-year medical students (N=66)  *The exact number of medical students in their first and second year was not specified. | Experimental group (N=33):  Participants received a five-minute lesson to become familiar with iPads loaded with the VR application. Subsequently, participants accessed a range of neuroanatomical structures | Control group (N=33):  Used traditional paper methods that showed the same neuroanatomical structures present in the VR application. | To evaluate the short-term and long-term influence of 3D-VR technology on learning outcomes, when compared to traditional teaching models. | • Male and female participants in the control group scored statistically significantly higher in their post-test than in their pre-test (Males: p<0.001; Females: p=0.01).  • Male and female participants in the intervention group scored statistically significantly lower in their post-test than in their pre-test (Males: p<0.001; Females: p=0.03).  • Male and female participants in the control and intervention groups scored statistically significantly higher in their second post-test than in their first post-test (VR Group: Males: p<0.001, Females: p=0.03; Control group: Males: p<0.001, Females: p=0.03). |
| **Augmented Reality (AR) Resources** | | | | | | |
| Küçük et al. 2016 (Turkey) [58] | Mixed-methods study | Second year medical students (N=70) | Experimental Group: Participants were taught by traditional presentation material such as 2D pictures, graphs, and text and had access to the MagicBook mobile Augmented Reality (mAR) App that focused on the ascending and descending pathways; MagicBook consisted of 6 3D video animations, 3D human anatomy model and two diagrams which were supplementary material for the experimental group only  Participants used MagicBook to review the teaching material  (N=34) [females=16; males=18] | Control Group:  Participants were taught by traditional presentation material such as 2D pictures, graphs, and text  Participants used a traditional textbook to review the teaching material | To determine the effects of learning anatomy via mAR on medical students’ academic achievement and cognitive load. | • Students from the experimental group were statistically significant more successful (p<0.05) than students from the control group and were statistically significantly found to have lower cognitive load compared to students from the control group (p<0.05). |
| Henssen et al. 2020 (Netherlands) [55] | Randomized Controlled Study | First year medical students (N=23) & biomedical sciences students (N=8) | AR Group:  Practical Assignment 1:  Students received an overview of the anatomy of the human brain  Practical Assignment 2:  Students studied subcortical structures using the GreyMapp Augmented Reality application | Control Group:  Practical Assignment 1:  Students received an overview of the anatomy of the human brain  Practical Assignment 2:  Students studied subcortical structures using cross-sections (anatomical drawings of transverse sections of the human brain) | To investigate the differences on test scores, cognitive load, and motivation after neuroanatomy learning using AR applications or using cross-sections of the brain | • No statistically significant differences were observed between the pre and post-test scores of the two groups.  • Post-test scores from both groups were significantly higher than their pre-test scores; post-test scores for the control group were significantly higher than post-test scores for the GreyMapp-AR group.  • Statistical analysis of the three components of the post-test showed that students from the control groups scored significantly better on the third part of the test (cross-sections) than students from the GreyMapp AR group. |
| Cercenelli et al. 2024 (Italy) [72] | Not specified | Second-year medical students (N=70) | AR Group:  Had access to the AEducAR3.0 platform | N/A | To explore the effectiveness of the AEducAR3.0 hybrid platform which combines AR with 3D models in allowing students to study neuroanatomy at differrent learning levels (notional learning, notional learning in context and topographical learning). | • Students achieved a higher correct response rate for the quiz of learning level two when compared to the correct response rate (70% ±4%) for the quizzes of learning levels 1 and 3 (60% ±7% and 58% ±5%, respectively) (Learning level 1 vs Learning level 2: p=0.04; Learning level 2 vs Learning level 3: p=0.02). |
| Zeedzen-Scheffers et al. 2024 (Netherlands)  [73] | Randomized Controlled Study | First year (N=5) and second year (N=23) medical and biomedical sciences students  Total number of participants N=28  *The exact number of medical students and biomedical sciences students in their first and second year was not specified. | AR Group:  Had access to the GreyMapp AR resource to complete their preparatory assignment prior attending their body donor-based education.  Preparatory Assignment 1:  Students received an overview of the anatomy of the human brain | Control Group:  Had access to Sobotta anatomical atlas resource to complete their preparatory assignment prior attending their body donor-based education. | To investigate the impact of the GreyMapp resource on students’ academic achievement when used as a preparatory teaching tool by students prior attending their body donor-based education session, and the effect of AR versus traditional resources on students’ cognition. | • Both the AR and control groups showed significant improvement from pre- to post-test scores (p<0.001), however, there were no statistically significant differences between the groups in the MRT scores (p=0.09) or in their pre- and post-test score comparisons (p=0.35). |
| **Mixed Reality (MR) Resource** | | | | | | |
| Pickering et al. 2022 (United Kingdom) [40] | Quasi-randomized control trial | Second year medical students (N=200) | MR Resource or Anatomy Drawing Screencast | N/A | To explore the impact of a MR resource focusing on the sensory and motor spinal pathways in comparison to a drawing screencasts multimedia video resource which was a pre-existing resource already embedded in the medical curriculum | • The percentage scores of students for the MCQ and SAQ sections and overall score were statistically significantly higher in the post-test irrespective of the resource the students have been exposed to (MR: p<0.001; Screencast: p<0.001).  • When absolute and normalising gains were applied to the data, it was shown that the only statistically significant increase in learning gain was observed in the MCQ section of the post-test for the screencast group (absolute gain, p < 0.003; normalising gain, p< 0.01) |
| **Utilization of a VR and an AR Resource** | | | | | | |
| Gurses et al. 2024 (Turkey) [74] | Not specified | Neurosurgery residents (N=40) & second-year medical students (N=200) | Neurosurgery residents:  Accessed the VR-Based 3D Models  Medical Students:  Accessed the AR-based 3D Models | N/A | To investigate the effectiveness of teaching neuroanatomy with AR and VR resources when cadaveric dissection is not available. | • Neurosurgery residents scored higher than medical students in the pre-test (7.5/10 vs 4.8/10).  • Both neurosurgery residents and medical students scored statistically significantly higher in their post-test scores (p<0.001 and p<0.001, respectively). |

AR: Augmented reality

CAL: Computer-assisted learning

E-learning: Electronic learning

LFB: Luxol fast blue

mAR: Mobile augmented reality

MCQ: Multiple-choice-questions

MR: Mixed reality

MRT: Mental rotation test

N/A: Not applicable

PLI: Polarized light imaging

VR: Virtual Reality

2D: Two-dimensional

3D: Three-dimensional

**Table 3:** Summarizes four strengths that were identified in some of the resources described in the 29 articles that were included in the final analysis.

| **Strengths identified in some of the resources described in the included studies** | **Type of Multimedia Resource** | | | | | **Total number of articles where resource exhibited this strength** |
| --- | --- | --- | --- | --- | --- | --- |
|  | **CAL (N=16)** | **Stereoscopic (N=4)** | **VR (N=3)** | **AR (N=5)** | **MR (N=1)** |  |
| Resource exposed students to radiological imaging scans (i.e., MRI, CT scans) | [39] [44] [45] [46] [48] | [70] | [71] | [73] | N/A | 8 |
| Resource contained interactive features such as quizzes, feedback, diagrammatic trees | [38] [42] [43] [49] [50] [51] [52] [69] | N/A | N/A | N/A | N/A | 8 |
| Resource contained a clinical application element | [38] [42] [43] [44] [49] [51] [67] [68] | N/A | N/A | [72] | [40] | 10 |
| Resource was designed based on evidence-based principles (i.e., cognitive load theory, principles of multimedia learning) | [43] [47] [51] [52] [67] | [70] | N/A | [55] [58] [73] | [40] | 10 |

AR: Augmented reality

CAL: Computer-assisted learning

CT: Computed tomography

MR: Mixed reality

MRI: Magnetic resonance imaging

VR: Virtual Reality

**Table 4:** Summarizes 12 existing weaknesses present across some of the resources described in the 29 articles that were included in the final analysis.

| **Weaknesses identified in some of the resources or the articles describing them** | **Type of Multimedia Resource** | | | | | **Total number of articles where resource or article exhibited this weakness** |
| --- | --- | --- | --- | --- | --- | --- |
|  | **CAL (N=16)** | **Stereoscopic (N=4)** | **VR (N=3)** | **AR (N=5)** | **MR (N=1)** |  |
| Resource was not accessible | [38] [39] [42] [43] [45] [46] [47] [48] [49] [52] [69] | [41] [53] [70] | [56] [57] [71] | [55] [58] [73] [74] | [40] | 22 |
| Link provided to access resource was dysfunctional | [44] | N/A | N/A | N/A | N/A | 1 |
| Information regarding the description of resource was extremely limited | [38] [39] [44][45] [47] | [41] | [71] | N/A | [40] | 8 |
| Article provided extremely limited information on how user interacts while using the resource or how user is expected to use the resource | [38] [39] [44][45] [52] | [41] | [71] | [58] | [40] | 9 |
| Article does not provide adequate information on how resource was created to allow its recreation by others | [38] [39] [42] [43] [47] [50] [52] | N/A | [56] [71] | N/A | [40] | 10 |
| No information was provided on the anatomical structures present in the resource | [38] | [41] | N/A | N/A | N/A | 2 |
| Inadequate information regarding the anatomical structures present in the resource | [44] [45] [47] [48] [52] [67] | N/A | N/A | N/A | N/A | 6 |
| Article did not provide adequate supplementary material (i.e., figures illustrating screenshots from a resource) | [38] [39] [47] [48] | N/A | [56] [57] [71] | N/A | [40] | 8 |
| Inadequate information on methods section (i.e., design and structure of pre-test/ post-test, amount of time students were given to complete such tests or questionnaires or surveys) | [38][39] [42] [47] [49] [50] [52] [67] [68] | [41] | [56] [71] | [55] [58] | N/A | 14 |
| Inconsistencies present in the results section | [39] [47] [49] [50] | N/A | [71] | N/A | N/A | 5 |
| Limited use of multimedia elements (**≤** three out of the six multimedia elements) | [38] [39 [42] [44] [45] [46] [47] [49] [52] [68] | [41] [70] | [56] [57] | [55] [73] [74] | N/A | 17 |
| Resource focuses purely on structural neuroanatomy | [38][39] [45] [46] [47] [52][68] [69] | [41] [53] [54] [70] | [56] [57] [71] | [58] [73] [74] | [40] | 19 |

AR: Augmented reality

CAL: Computer-assisted learning

MR: Mixed reality

VR: Virtual Reality

**Appendix A:** Details which of the three databases that were used for the literature search of the systematic review use medical subject headings (MeSH) terms, which of the search strings that were used are MeSH terms and provides the full search strategy for the PubMed database.

**Table 1:** Lists the three databases that were used for the literature search of the systematic review and whether they use medical subject headings (MeSH) terms or an alternative to MeSH terms.

| **Database** | **Uses medical subject headings (MeSH) (Yes/No)** | **Uses an alternative to medical subject headings (MeSH)** |
| --- | --- | --- |
| PubMed | Yes | N/A |
| ERIC (ProQuest) | No | • Free text  • Indexing terms |
| Scopus | No | • ERIC thesaurus |

**Table 2:** Lists the search strings that were used in the search strategy and whether each search string is a Medical Subject Heading (MeSH) keyword.

| **Search string** | **Medical Subject Heading (MeSH)** | **Medical Subject Heading (MeSH) Term** | **Non- Medical Subject Heading (MeSH)** |
| --- | --- | --- | --- |
| neuroanatomy | ✔ | “Neuroanatomy” [MeSH] |  |
| learning | ✔ | “Learning” [MeSH] |  |
| e-learning |  | | ✔ |
| education | ✔ | “Education” [MeSH] |  |
| teaching | ✔ | “Teaching” [MeSH] |  |
| multimedia | ✔ | “Multimedia” [MeSH] |  |
| multi-media |  | | ✔ |
| video | ✔ | “video tape recording” [MeSH] |  |
| resource |  | | ✔ |
| interactive |  | | ✔ |
| animation |  | | ✔ |
| virtual reality | ✔ | “virtual reality” [MeSH] |  |
| augmented reality | ✔ | “augmented reality” [MeSH] |  |
| online |  | | ✔ |
| student | ✔ | “students” [MeSH] |  |

**Table 3:** Provides a comprehensive search strategy for the PubMed database. N/A: Not applicable; ALL: A field code that signifies that the search is anywhere in the record or that all fields are searched; PDAT: A field code that signifies that limits results based on publication date.

| **Search string** | **Medical Subject Heading (MeSH) Term(s)** | **Free-text keyword** | **Truncation/**  **Variations** | **Field Code** |
| --- | --- | --- | --- | --- |
| Neuroanatomy | “Neuroanatomy” [MeSH] | neuroanatomy | N/A | [MeSH]  [ALL] |
| Learning | “Learning” [MeSH] | learning, e-learning, | learn* |  |
| Education | “Education” [MeSH] | education | educat* |  |
| Teaching | “Teaching” [MeSH] | teaching | teach* |  |
| Multimedia | “Multimedia” [MeSH] | multimedia, multi-media | multimed* |  |
| Video | “Video recording” [MeSH] | video | video* |  |
| Resource | N/A | resource | resourc* |  |
| Interactive | “User-Computer –Interface" [MeSH] | interactive | interact* |  |
| Animation | N/A | animation | animat* |  |
| Virtual reality | “Virtual Reality” [MeSH] | virtual reality | virtual* |  |
| Augmented reality | “Augmented Reality” [MeSH] | augmented reality | augment* |  |
| Online | “Internet-based-intervention" [MeSH] | online | online* |  |
| Student | “Students, Medical” [MeSH] | student, medical students | student* |  |
| Publication Date | N/A | N/A | 1994-2025 | [PDAT] |

**Full search strategy for PubMed database using MeSH terms and free-text:**

("Neuroanatomy"[MeSH] OR "neuroanatomy") AND

("Learning"[MeSH] OR "e-learning" OR "Education"[MeSH] OR "Teaching"[MeSH] OR

"Multimedia"[MeSH] OR "multi-media" OR "Video Recording"[MeSH] OR "video" OR

"Resource" OR "Interactive" OR "User-Computer Interface"[MeSH] OR "Animation" OR

"Virtual Reality"[MeSH] OR "Augmented Reality"[MeSH] OR "Online" OR "Student [MeSH]) AND ("1994"[Date - Publication] : "2025"[Date - Publication])

**Appendix B:** Data Extraction for the 29 articles included in the final analysis

**Table 1:** Data extraction of the 29 articles summarized according to the PICO(s) model.

| **Aim of Study** | **Study Design** | **Population description and number of participants (N=)** | **Exposure** | **Comparator** | **Measure** | **Outcome(s)** |
| --- | --- | --- | --- | --- | --- | --- |
| **Computer-Assisted Learning Resources** | | | | | | |
| **Evaluation of neuroanatomical training using a 3D visual reality model**  Brewer, D., Wilson, T., Eagleson, R., & de Ribaupierre, S. (2012). Evaluation of Neuroanatomical Training using a 3D Visual Reality Model. *Studies In Health Technology And Informatics*, *173*, 85-91. https://doi:10.3233/978-1-61499-022-2-85 | | | | | | |
| To evaluate the effectiveness of a CAL digital lab resource in improving the participants’ knowledge of neuroanatomy post-lecture and either pre or post the gross lab practical | Randomized Controlled Study | Second year health science students (N=13) and second year medical students (N=118) at the University of Western Ontario | Health science students:  Group 2/ 2D Group: Pre-Digital Lab or Group 3/3D Group: 3D Pre- Digital Lab  Medical students:  3D Pre- Digital Lab | Health science students:  Control group  Medical students:  No comparator for medical students as they experienced both the digital lab and gross lab but in a different order. | Health science students:  12-minute post-test, a survey that aimed to identify whether students had prior knowledge in neuroanatomy or experience and a 6-minute mental rotation test (MRT).  Medical students:  Undertook a knowledge post-test and a user-interface questionnaire/survey that lasted 20 minutes after attending the 3D digital lab. | Health science students:  No statistically significant differences were found between the three groups neither between the two experimental groups. Post-test scores of students in group 3 were the highest from all three groups.  No significant correlations were observed between health science students’ MRT scores and the type of intervention they received, or students’ MRT scores and post-test scores of each individual group or prior experience.  Medical students:  There were no statistically significant differences between the two groups.  On a Likert scale out of 7, medical students from both groups rated the 3D digital lab with 5.1 and reported that it assisted them in finding and learning anatomical structures more than a plastinated brain model did.  Students who had the gross lab before the 3D lab found the whole experience much more useful than the students who had the 3D digital lab first. |
| **Attitudes to e-learning, learning style and achievement in learning neuroanatomy by medical students**  Svirko, E., & Mellanby, J. (2008). Attitudes to e-learning, learning style and achievement in learning neuroanatomy by medical students. *Medical Teacher*, *30*(9-10), e219-e227. https://doi:10.1080/01421590802334275 | | | | | | |
| To evaluate how successful a neuroanatomy CAL course was at encouraging students to employ a deep approach to learning, the factors that influence students’ choice over which approach they employ for their learning as well as identify whether there is a correlation between the learning approach students employ and their academic performance | Not specified | 205 pre-clinical second year medical students (N=205) at Oxford University | Neuroanatomy CAL course | No comparator was present | “Approach to learning” Questionnaires comprised of  1) R-SPQ-2F that consisted of 10 deep and 10 surface questions rated on a 5-point scale giving a maximum deep and surface approach score of 50; R-SPQ-2FS needed to be completed by students twice for the purposes of the CAL neuroanatomy course and the students’ general academic work  2) SPQ that consisted of 9 items  3) a computer anxiety item where students needed to rate the following statement “I find using a computer makes me feel anxious” on a 5-point scale  4) the CAL evaluation questionnaire where students needed to rate two statements on a 5-point scale regarding the resource’s ease of use and amount of information present  Computer-Based Neuroanatomy Formative Assessment (NFA):  Consisted of 40 MCQs that required students to 1) identify labels that indicated parts of the brain or spinal cord, 2) identify the origin or destination of neuronal pathways, 3) identifying whether a statement was true or false and 4) identify symptoms for disorders or identify disorders from symptoms | Students reported higher surface approach scores and lower deep approach scores for the CAL course than for their studies in general  Most students (61.1%) reported that it was easy for them to follow the CAL course  Around 40.2% of the students stated that they strongly disagreed or disagreed with the statement about whether students enjoyed the CAL course  Most students (74.5%) were happy with the amount of information the CAL course contained  There was a positive correlation between student statements that reported that students enjoyed the CAL course, thought it was easy to follow and did not want it to contain less information and students’ scores on employing a deep approach to learning.  Similarly, there was a negative correlation between student statements that reported that students enjoyed the CAL course and did not want it to contain less information and students’ scores on employing a surface approach to learning. |
| **Computer-based neuroanatomy laboratory for medical students**  Lamperti, A., & Sodicoff, M. (1997). Computer-based neuroanatomy laboratory for medical students. *The Anatomical Record*, *249*(3), 422-428. https://doi:10.1002/(sici)1097-0185(199711)249:3<422::aid-ar14>3.0.co;2-q | | | | | | |
| Developed a CAL program that consisted of a computerized atlas, a laboratory guide and clinical problem-solving section, to replace the traditional glass-slide/microscope-based laboratory component of a course and compare the student performance between students who received the traditional program and students who received the CAL program | Not specified | First year medical students (N=185) | Group 1:  2-hour computer-directed study with access to CAL program and 1,5-hour self-study time  Group 2:  1,5-hour self-study time and a 2-hour computer-directed study with access to CAL program | No comparator was present | Comparison of practical exams of students who were taught with the traditional program with practical exams of students who were taught with the CAL program.  Students undertook three exams each of which consisted of:   1. MCQ Written Exam 2. Practical SPOT exam   First two exams contained 25 MCQs and 15 practical questions whereas the last exam consisted of 40 MCQs and 33 practical questions. | Students who were trained via the CAL resource who scored significantly higher (p<0.0001) than the students who were taught by the traditional method.  Overall, student opinion revealed that students were satisfied with the CAL resource but still thought that the presence of faculty members was necessary to help answer students’ questions and that the faculty-led review sessions were useful to students.  The CAL resource was rated as “good-excellent” by 90% of the students and it was reported that students have been using the resource at their own time as well and before exams as well.  Evaluation of faculty’s opinion on the resource revealed that all faculty agreed that the resource had many benefits mainly for the students but not for the interaction between students and the faculty. |
| **Effects of interactive instructional techniques in a web-based peripheral nervous system component for human anatomy**  Allen, E., Walls, R., & Reilly, F. (2008). Effects of interactive instructional techniques in a web-based peripheral nervous system component for human anatomy. *Medical Teacher*, *30*(1), 40-47. https://doi:10.1080/01421590701753518 | | | | | | |
| To determine the effect of web-based interactive instructional techniques on written exam item performance and the differences between student opinions of the benefit level of five different types of interactive learning objects (patient case studies, review games, stimulated interactive patients, flashcards, and quizzes) used in a human anatomy course | Not specified | First year medical students (N=856) at West Virginia University, West Virginia, United States | Treatment 1:  Didactic lecture & dissection lab for years 1995-1997  Treatment 2:  Didactic lecture & dissection lab & PowerPoint lecture notes on CD video game console for year 2000  Treatment 3:  Didactic lecture & dissection lab & web-based lecture notes, & web- based interactive learning objects for years 2001-2005 | No comparator was present | Instructional Design Questionnaires:  Consisted of 14 items that needed to be rated on a 5-point Likert scale and 3 open-ended questions asking students about their likes, dislikes, and suggestions on instructional design for years 2001-2005 (N=260)  PNS content of written exams:  Student scores for the PNS content questions were available for years 1995-1997 & 2001-2005 only | A statistically significant difference was observed between treatments 1 and 3 but not between treatments 1 and 2 or treatments 2 and 3; students who received treatment 3 correctly answered a higher number of questions compared to students who received any of the other two types of treatment.  Student results for the benefit level of the 5 different interactive learning objects revealed that students rated on 5-point Likert scale the flashcards with 3.26, the quizzes with 3.52, the review games with 3.62, the patient case studies with 3.63 and the stimulated interactive patients with 4.31.  In terms of the benefit level of all 5 interactive learning objects, the simulated interactive patients (SIP) learning object was significantly higher than patient case studies (p<0.001), review games (p<0.001), flashcards (p<0.001) and quizzes (p<0.001)  Review games were rated significantly higher than flashcards (p<0.01)  Findings from the open-ended questions on the questionnaire revealed that most students found the SIP as the most helpful learning object of all five |
| **Web-Based Software to Assist in the Localization of Neuroanatomical Lesions**  Lewis, E., Strike, M., Doja, A., Ni, A., Weber, J., Wiper-Bergeron, N., & Sell, E. (2011). Web-Based Software to Assist in the Localization of Neuroanatomical Lesions. *Canadian Journal Of Neurological Sciences / Journal Canadien Des Sciences Neurologiques*, *38*(2), 251-255. https://doi:10.1017/s0317167100011422 | | | | | | |
| To evaluate the educational effectiveness of a web-based neuroanatomy localization application | Randomized Controlled Study | Second year medical students (N=39) | Intervention Group: Localization Application  (N=20) | Control Group:  Two Neuroanatomy Textbook Resources  (N=19) | 30-minute Case-Based MCQ Test:  Consisted of three  clinical cases and their associated MCQs and needed to be completed by exposure and comparator groups after they had access to their assigned resource. Maximum score students could score was 10.  5-minute Test Questions: Completed by exposure and comparator groups after they switched and had access to the assigned resource of the opposite group  10-minute Questionnaire:  A six-item 4-point Likert scale questionnaire that aimed to assess participants’ subjective preference of the application and its perceived utility for clinical and educational purposes. | More than 84% of participants from both the intervention and control group reported spending more than 20 hours on the internet.  Proficiency in computers was rated as “good” or “very good” by most participants of both groups.  Student scores for the MCQ test were 67.5% and 54.7% for the intervention and control group, respectively.  The intervention group showed a significantly higher (p=0.028) mean raw score than the control group  Overall, student feedback on the localization application was favourable except for one statement where students would not prefer the localization application over an anatomical textbook resource. |
| **Enhancing neuroanatomy education using computer-based instructional material**  Ruisoto Palomera, P., Juanes Méndez, J., & Prats Galino, A. (2014). Enhancing neuroanatomy education using computer-based instructional material. *Computers In Human Behavior*, *31*, 446-452. https://doi:10.1016/j.chb.2013.03.005 | | | | | | |
| To develop a computer-based tool to explore neuroanatomy based on three- dimensional images and to compare whether the educational value assigned by students varies according to their visuospatial ability | Not specified | Medical students (N=65)  *Year of study was not specified | Computer-Based Tool | No comparator was present | Online survey:  A 4-item 7-point Likert scale survey to evaluate students’ perceptions of the anatomical functional viewer’s educational value  Computer-Based Surface Development Test:  Was completed by the students to evaluate their visuospatial ability. The test contained 12 items and maximum time for completing the test was 14 minutes. | Online survey:  No statistically significant differences were observed between students who showed high visuospatial ability and students who showed low visuospatial ability in any of the four items of the survey (p>0.01). |
| **Development and assessment of a new 3D neuroanatomy teaching tool for MRI training**  Drapkin, Z., Lindgren, K., Lopez, M., & Stabio, M. (2015). Development and assessment of a new 3D neuroanatomy teaching tool for MRI training. *Anatomical Sciences Education*, *8*(6), 502-509. https://doi:10.1002/ase.1509 | | | | | | |
| To develop a computerized (3D) neuroanatomy teaching tool for training medical students to identify subcortical structures on a magnetic resonance imaging (MRI) series of the human brain and to assess its efficacy. | Randomized Controlled Study | First year medical students (N=73)  11 students were excluded (8 from the experimental group and 3 from the control group) due to prior exposure to neuroanatomy  Total number of participants: N=62 | Experimental Group:  Neuroanatomy lecture with 3D neuroanatomy teaching tool (N=33) | Control Group:  Neuroanatomy lecture with traditional methods  (N=29) | 5-minute survey: was distributed at the beginning of the workshop to obtain information about baseline characteristics  10-minute Purdue Visualization of Rotations Test: Was distributed after the survey and aimed to assess students’ intrinsic spatial reasoning abilities  30-minute assessment test: was completed after students from both groups received the same lecture; students needed to answer 32 identification questions from MRI slices screenshots  5-minute survey: completed at the end of the workshop to assess students’ satisfaction levels and to report their self-assessed confidence level in neuroanatomy | There were no statistically significant differences between the MRI identification scores across the experimental and control groups.  A statistically significant difference (p<0.01) was observed for questions that involved C-shaped structures as the experimental group outperformed the control group by an estimated difference in average score of 15.8%.  A statistically significant rank correlation (p=0.02) was observed for question four by the experimental group in terms of the helpfulness of the session for visualizing 3D structures and spatial relationships of the brain.  All experimental group participants agreed that they would recommend the 3D neuroanatomy teaching tool for use in future classes as well as use it at their own time. |
| **Evaluation of an Online Three-Dimensional Interactive Resource for Undergraduate Neuroanatomy Education** Allen, L., Eagleson, R., & de Ribaupierre, S. (2016). Evaluation of an online three-dimensional interactive resource for undergraduate neuroanatomy education. *Anatomical Sciences Education*, *9*(5), 431-439. https://doi:10.1002/ase.1604 | | | | | | |
| To examine the educational efficacy of a newly developed 3D neuroanatomy module | Randomized Crossover Study | Second year medical students (N=47) at the University of Western Ontario | Group A:  Initially had access to the online 3D learning module and subsequently had access to the cadaveric laboratory session  Group B:  Initially had access to the cadaveric laboratory session and subsequently had access to the online 3D learning resource | No comparator was present | Pre-test:  Consisted of 15 questions to assess students’ knowledge on spatial relationships in neuroanatomy; was completed by students prior they had any exposure to any of the two experimental groups  Santa Barbara Solids Test:  Was completed by students to measure their spatial ability  Post-test:  Consisted of 15 questions to assess students’ knowledge on spatial relationships in neuroanatomy after they had been exposed to the resource, they had been assigned  Final 15-question knowledge assessment:  Was completed by students after they had been exposed to resources of both experimental groups to assess their knowledge on information present on both modalities  5-point Likert scale questionnaire:  assessed students’ attitudes and perceptions over the efficacy of the two learning modalities, personal preference over a learning modality and level of attraction or repulsion toward different types of learning modalities | The only statistically significant difference between Groups A and B was observed for the post-test scores as it was shown that students who accessed the 3D learning module before accessing the cadaveric laboratory session performed significantly better on this test (p<0.01)  Statistical analysis of scores within each group revealed that students in both groups scored significantly higher on the post-test than on the pre-test (p<0.01)  Only the student scores on the final 15-question knowledge assessment from Group A were shown to be significantly higher than the student scores on the post-test (p<0.01)  Spatial ability was shown to be positively correlated with higher scores across the anatomy knowledge assessments.  Responses from the questionnaire revealed that the 3D model assisted students with visualization of structures and with their understanding as well unlike traditional resources. |
| **Analysis of Traditional Versus Three-Dimensional Augmented Curriculum on Anatomical Learning Outcome Measures**  Peterson, D., & Mlynarczyk, G. (2016). Analysis of traditional versus three-dimensional augmented curriculum on anatomical learning outcome measures. *Anatomical Sciences Education*, *9*(6), 529-536. https://doi:10.1002/ase.1612 | | | | | | |
| To analyse the examination performance of students on questions covering material taught with traditional learning formats versus material covered with traditional formats augmented with digital 3D technology. | Not specified | Graduate medical students (N=51)  Upper-level undergraduate medical students (N=5)  Total number of students: N=56  *Year of study is not specified by authors | Traditional teaching material augmented with computerized 3D teaching tools | Traditional teaching material | Laboratory Exam:  Consisted of 25-50 spot exam questions depending on the exam;  students had 1 minute for each question and completed 4 laboratory examinations in total  Written Examinations:  Consisted of 27 MCQs and students completed 4 written examinations in total  Student Survey:  Distributed at the end of semester to determine whether student perceptions over the usefulness of each teaching modality correlates to student performance; students needed to rate traditional and augmented teaching tools on a 5-point Likert scale. Student also answered 9 questions by providing free comments | Student performance for questions covering material taught with 3D teaching tools was statistically significantly higher (p<0.0001) than for questions covering material taught with traditional teaching methods  Students performed significantly better (p<0.0001) on cadaveric SPOT exam questions that were taught with 3D teaching tools than for cadaveric SPOT exam questions taught with traditional teaching methods  Higher undergraduate GPA was positively correlated with better overall performance (p<0.0005)  Survey results revealed that students rated traditional learning tools as more useful than 3D teaching tools, however, a combination of both assists students with the transition from 2D to 3D.  Students reported that overall, traditional learning tools assisted them with associating a name with a structure whereas 3D learning tools allowed them to develop a deeper understanding over the location of a structure and spatial relationships as well. |
| **Teaching neuroanatomy using computer-aided learning: What makes for successful outcomes?**  Svirko, E., & Mellanby, J. (2017). Teaching neuroanatomy using computer-aided learning: What makes for successful outcomes?. *Anatomical Sciences Education*, *10*(6), 560-569. https://doi:10.1002/ase.1694 | | | | | | |
| To compare the approach to learning that the students adopted toward the Oxford University’s neuroanatomy CAL course with their approach to the more traditional aspects of their neuroanatomy instruction and examine whether students’ approach to learning related to their performance in tests and examinations | Not specified | Second year medical students (N=869) | Neuroanatomy CAL course | Traditional neuroanatomy course | 45—minute Formative Neuroanatomy Assessment:  Consists of MCQs  Neuroscience Examination:  -25-minute MCQ paper  -40-minute Essay Question  “Approach to Learning” Questionnaires:  comprised of  1) the revised two-factor study process questionnaire (R-SPQ-2F) that consisted of 10 deep and 10 surface questions rated on a 5-point scale that eventually add up to give each a maximum deep and surface approach score of 50; R-SPQ-2FS was completed by students twice for the purposes of the CAL neuroanatomy course and the students’ general academic work  2) shortened study process questionnaire (SPQ) that consisted of 9 items  3) a computer anxiety item where students needed to rate the following statement “I find using a computer makes me feel anxious” on a 5-point scale (1=rarely true; 5=usually true);  and 4) the CAL evaluation questionnaire where students needed to rate the following two statements on a 5-point scale (1= strongly disagree; 5= strongly agree): statement 1: I found the CAL course easy to follow; statement 2: I enjoyed the CAL course; statement 3: “I would like the neuroanatomy CAL to contain: 1) Less information ; 2: About the same amount of information, 3) More information. | Students’ deep approach scores were significantly lower for the CAL course than for the traditional neuroanatomy course (p<0.001)  Students’ surface approach scores were significantly higher for the CAL course than for the traditional neuroanatomy course (p<0.001)  Students’ deep approach to the CAL course was positively correlated with students’ marks on the formative neuroanatomy assessment (p<0.001)  Students who spent more time on the CAL course were more likely to score higher in the formative assessment.  Overall, students who showed deep approach to learning seemed to have enjoyed the CAL course and its component as well as score better in the formative assessment |
| **A Multimedia Educational Module for Teaching Early Medical Neuroanatomy**  Welch, M. C., Yu, J., Larkin, M. B., Graves, E. K., & Mears, D. (2020). A Multimedia Educational Module for Teaching Early Medical Neuroanatomy. *MedEdPORTAL : the journal of teaching and learning resources*, *16*, 10885. <https://doi.org/10.15766/mep_2374-8265.10885> | | | | | | |
| To assess the effectiveness of a neuroanatomy multimedia learning module that was developed and was offered as optional study aids to pre-clinical medical students | Not specified | First year medical students  (N=314) | Neuroanatomy multimedia learning module | No comparator was present | Practical examination:  11 questions completed by students to assess their understanding after being exposed to the neuroanatomy learning module  External Topography Survey:  A 5-point Likert scale survey completed by 160 students only to provide feedback on external topography learning tools; these students also completed a survey on the usage of external topography learning tools  Internal Topography Survey:  A 5-point Likert scale survey completed by 154 students only to provide feedback on internal topography learning tools; these students also completed a survey on the usage of internal topography learning tools | Overall, students thought that the videos of external and internal topography they watched helped them prepare for the dissection; both the internal and external topography post-dissection review slides helped them prepare for dissection but also learn anatomy; despite this, survey results demonstrated that not all participants reviewed these slides prior attending the lab but all of them reviewed them after performing the dissection in the lab |
| **E-Learning Three-Dimensional Anatomy of the Brainstem: Impact of Different Microscopy Techniques and Spatial Ability**  Cappellen van Walsum, A., & Henssen, D. (2022). E‐Learning Three‐Dimensional Anatomy of the Brainstem: Impact of Different Microscopy Techniques and Spatial Ability. *Anatomical Sciences Education*, *15*(2), 317-329. https://doi:10.1002/ase.2056 | | | | | | |
| To evaluate the effect of spatial ability on cross-sectional e-learning brainstem anatomy and examine the learning outcomes of students working with PLI images and learning outcomes of students working with LFB images | Randomized Controlled Study | Second year medical students  (N=38) [females=19; males=19] at University Medical Center in Nijmegen, Netherlands | Group 1:  Students were exposed to  E-Learning Modules 1, 2 and 4  (N=19)  Group 2:  Students were exposed to  E-Learning Modules 1, 3 and 4  (N=19) | No comparator was present | Mental Rotation Test (MRT):  Assessed students’ spatial abilities  Pre-Intervention Test:  Completed prior students accessed either Learning modules 1 and 2 or 1 and 3 to assess their knowledge of brainstem anatomy; contained 20 two-choice questions (16 questions tested students on relationships between structures of the brainstem and the remaining 4 tested students on the craniocaudal orientation of structures)  Post-Intervention Test:  Was identical to pre-intervention test and was completed after students accessed either e-learning modules 1 and 2 or 1 and 3 to assess their knowledge of brainstem anatomy  Long-Term Anatomical test:  Completed by students after accessing e-learning module 4 to assess students’ long-term retention of memory  Qualitative Interviews:  Semi-structured qualitative interviews were conducted with 10 student volunteers | No statistically significant differences were observed between the MRT scores of male and female medical students (p=0.134)  No statistically significant differences were observed between the pre-intervention, post-intervention and long-term test scores of the two groups  Post-intervention and long-term anatomical test scores were significantly higher than pre-intervention test scores for both groups indicating improvement irrespective of the e-learning module students were assigned  Statistical analysis showed that MRT scores were significantly correlated with pre- and post-intervention test scores, and a significant correlation between pre- and post-intervention test scores  Qualitative results revealed that students were not happy with their white matter brain dissection assignments being replaced by electronic e-learning modules and the lack of hands-on activities due to the presence of e-learning modules.  Students expressed satisfaction with the e-learning modules as they allowed them to be “in-control” of their learning, maintain their attention and helped them remain focused.  Students from both groups expressed that the amount of background technical information they were provided with was excessive and not necessarily relevant to what they need to know while reading the slides. Tactics used by students while learning brainstem anatomy included studying the brainstem by focusing on one of its three regions at a time, visualization of sections of the brainstem (i.e., white matter tracts for students using PLI figures) by color |
| **Neuroanatomy of the spinal pathways: Evaluation of an interactive multimedia e-learning resource**  Javaid, M.A., Schellekens, H., Cryan, J.F. and Toulouse, A. (2020b). Neuroanatomy of the spinal pathways: Evaluation of an interactive multimedia e-learning resource [version 1]. MedEdPublish, 9:88. <https://doi.org/10.15694/mep.2020.000088.1> | | | | | | |
| To evaluate the impact of an interactive CAL resource on medical students’ understanding of neuroanatomy of the spinal pathways | Single Blinded Controlled Study | 34 graduate medical students & 46 undergraduate medical students & 5 clinical therapy students | Experimental Group [N=28]:  Interactive CAL resource on spinal pathways developed by Javaid and colleagues  No-use Group [N=36]:  No use of the interactive CAL resource developed by Javaid and colleagues, neither use of the Functional Neuroanatomy resource developed by Krebs and colleagues | Control Group [N=21]:  Functional Neuroanatomy resource developed by Krebs and colleagues | Pre-test (Quiz 1):  Consisted of 24 MCQs  Completed by students before becoming exposed to their assigned resource to measure their neuroanatomy  knowledge on the spinal pathways  Pre-test was completed by 154 students  Post-test (Quiz 2):  Completed by students after becoming exposed to their assigned resource to measure their neuroanatomy knowledge on the spinal pathways  Pre-test was completed by 138 students  53 participants were excluded since they did not complete both the pre-test and post-test  Likert-scale Questionnaire:  Completed by participants in the control and experimental groups only to assess their perceptions and attitudes toward their assigned resource, the usability of the resource as well as the mental effort the resource required them to put into.  Likert-scale questions focused on how each resource helped students 1) learn the neuroanatomy of the spinal pathways, 2) visualize relevant neuroanatomical structures in 3D and 3) comprehend the clinical correlates relevant to the spinal pathways | No statistically significant differences were observed for participants in each of the three groups regarding their percentage of correct answers in the pre- or the post-test in relation to the questions’ level of difficulty.  For all three groups, there was a statistically significant difference in students’ neuroanatomy knowledge on the spinal pathways between the pre- and post-tests for the easy and difficult questions and total scores overall.  Students in all three groups performed statistically significantly better in the post-test.  No statistically significant difference was noted across students’ median normalized learning gain scores.  A statistically significant difference was observed for the median normalized learning gains of participants in the no-use group for the easy versus difficult questions (p<0.001).  Compared to the CAL resource used by the control group, the assigned CAL resource for the experimental group was shown to be more useful (p<0.05) in helping students learn clinical correlates relevant to the spinal pathways, requiring less mental effort in terms of students learning to identify neuroanatomical structures on CT and MRI scans (p<0.05), clinical correlates relevant to the spinal tracts (p<0.01) and learning to locate lesions within the spinal tracts (p<0.05).  The most significant correlation (p<0.01) observed was for the usefulness of the CAL resource developed by Javaid and colleagues in terms of summarization of the content for the easy questions.  A statistically significant correlation was observed between the students’ percentage of correct answers in the control group and their ratings regarding the usefulness of the cross sections where the spinal tracts were labelled available in their assigned resource (p<0.05). |
| Creating evidence-based engaging online learning resources in neuroanatomy  Booker, J., Woodward, C., Taylor, C., Robson, A. and Border, S. (2024) Creating evidence-based engaging online learning resources in neuroanatomy. Anat Sci Educ, 17(3), 605-619. <https://doi.org/10.1002/ase.2367> | | | | | | |
| To compare the learning gain of using text-based versus video resources from Soton Brain Hub online educational platform | Cross-sectional study | Second year medical students (N=76) | Online videos group:  Online video resources from Soton Brain Hub focusing on ‘pain pathways’ (N=53) | Text resource group:  Paper copy of a text-based resource focusing on ‘pain pathways’ (N=23) | Participants from both groups completed a pre-teaching MCQ (consisted of 25 five-option questions) prior accessing their assigned resource.  Participants from both groups completed a post-teaching MCQ (consisted of 25 five-option questions) after accessing their assigned resource.  *The questions in the pre- and post- MCQ were different, however, the questions’ level of difficulty matched.  Both groups completed another MCQ 3 weeks after accessing their assigned resource, to assess knowledge retention.  *The questions in the retention MCQ consisted of questions present in the pre- and post MCQs. | Students in the intervention group who had access to video resources from the Soton Brain Hub online educational platform had a statistically significantly higher average normalized learning gain than the control group who had access to a paper copy of text-based resources (p=0.030).  When both groups took the retention MCQ, it was shown that there was no statistically significant difference for the retained learning gain among the two groups (p=0.919). |
| GRAVEN: a database of teaching method that applies gestures to represent the neurosurgical approach’s blood vessels and nerves  Xuan, H., Zhong, J., Wang, X., Song, Y., Shen, R., Liu, Y., Zhang, S., Cai, J. and Liu, M. (2024). GRAVEN: a database of teaching method that applies gestures to represent the neurosurgical approach’s blood vessels and nerves. BMC Medical Education, 24(509), 1-9. https://doi.org/10.1186/s12909-024-05512-0 | | | | | | |
| To develop a database that’s focuses on intracranial anatomy and neurosurgical approaches, to help undergraduate medical students | Randomized Control Study | Undergraduate medical students (N=50)  *Year of study was not specified | GRAVEN group:  The neuroanatomy instruction for students within this group combined the traditional teaching methods used in the control group, and the GRAVEN database. | Control group:  The neuroanatomy instruction for students within this group was via traditional teaching methods (PowerPoint, Neuroanatomy textbooks: Rhoton’s Cranial Anatomy and Surgical Approaches and Neurosurgery Tricks of the Trade) | Participants from both groups completed a 25-question neuroanatomy test after receiving their neuroanatomy instruction.  Participants in the GRAVEN group completed a survey questionnaire regarding GRAVEN being a helpful resource to learn neuroanatomy, and an interesting method to teach neuroanatomy. | Students in the GRAVEN group scored statistically significantly higher in the test than students in the control group (p = 0.0026).  Survey responses from the 25 students in the GRAVEN group indicated that 84% found that the GRAVEN database enhanced their interest in neuroanatomy; and 68% felt it helped them learn neuroanatomy when combined with traditional teaching methods. |
| Effectiveness and satisfaction with virtual and donor dissections: A randomized controlled trial  Yun, Y.H., Kwon, H.Y., Jeon, S.K., Jon, Y.M., Park, M.J., Shin, D.H., Choi, H.J. (2024). Effectiveness and satisfaction with virtual and donor dissections: A randomized controlled trial. *Sci Rep* **14**, 16388 (2024). <https://doi.org/10.1038/s41598-024-66292-7> | | | | | | |
| To investigate medical students’ academic performance and satisfaction when utilizing virtual vs donor dissections. | Randomized Control Study | First year medical students (N=154) | Virtual group (N=71):  Accessed the Complete Anatomy app on a tablet to study structures of the diencephalon, telencephalon and structures associated with the third and lateral ventricles.  Study participants were divided in three classes (A, B,C). Participants in each class were randomly assigned a resource (virtual or donor dissection) and subsequently accessed the other resource. | Donor group (N=83):  Accessed donor dissections which were prepared in advance by tutors. | Quiz 1:  All study participants took Quiz 1 after accessing their first assigned resource (brain dissection or virtual dissection using a tablet)  Quiz 2:  All study participants took Quiz 2 after accessing their second assigned resource (virtual dissection using a tablet or brain dissection).  *Quizzes 1 and 2 consisted of seven image-based MCQs and took around 3-6 minutes to complete.  Survey:  Was completed by participants to evaluate their satisfaction of virtual dissection compared to donor dissection. Survey questions were rated on a 5-point Likert-type scale score. | Only in class A, the virtual group scored statistically significantly higher in Quiz 1 than the donor group (p<0.01). In classes B and C, the virtual group scored higher in Quiz 1 than the donor group, but no statistically significant differences were observed. The mean score of quiz 1 for all classes was statistically significantly higher in the virtual group than in the donor group (p<0.05)  No statistically significant differences were observed between the mean scores of Quiz 2 between the two groups within each class and across all three classes. |
| **Stereoscopic resources** | | | | | | |
| **Virtual and stereoscopic anatomy: when virtual reality meets medical education**  de Faria, J., Teixeira, M., de Moura Sousa Júnior, L., Otoch, J., & Figueiredo, E. (2016). Virtual and stereoscopic anatomy: when virtual reality meets medical education. *Journal Of Neurosurgery*, *125*(5), 1105-1111. https://doi:10.3171/2015.8.jns141563 | | | | | | |
| To develop and evaluate a virtual stereoscopic resource for neuroanatomy teaching | Randomized Control Study | Graduate medical students (N=84)  ***Year of study was not specified** | Group 1: Received a traditional lecture exhibiting 2D images  OR  Group 2:  Received a lecture that used interactive non-stereoscopic methods  OR  Group 3:  Received a lecture that used interactive stereoscopic methods to demonstrate 1 non-stereoscopic and 1 stereoscopic video | No comparator was present | Pre-test: Students were required to list structures of the limbic system  Written Theory Exam:  Students were required to list 10 structures of the limbic system that were pre-determined by the authors, therefore, students could score up to 10 points.  Lab Practicum:  Students were required to identify the 10 pre-determined structures of the limbic system on cadaveric specimens  Groups 2 and 3:  Were asked to describe the advantages and disadvantages of the methods by which they were instructed | Students in groups 2 and 3 scored significantly higher than students in group 1 in the post-test scores for their written theory exam (p<0.05)  Comparison of the mean values of the pre and post tests showed that there was a statistically significant improvement in the post-test scores of groups 2 and 3.  In terms of scores of the practical exam, there was a statistically significant difference in the mean scores between group 1 and groups 2 and 3.  Reported resource’s disadvantages by students in groups 2 and 3 included: eyestrain, absence of text and names of structures and lack of physical contact.  Overall, stereoscopy was not statistically shown to add any advantage to students’ knowledge when compared to students’ knowledge from the non-stereoscopic lecture. |
| **Stereoscopic neuroanatomy lectures using a three-dimensional virtual reality environment**  Kockro, R., Amaxopoulou, C., Killeen, T., Wagner, W., Reisch, R., & Schwandt, E. et al. (2015). Stereoscopic neuroanatomy lectures using a three-dimensional virtual reality environment. *Annals Of Anatomy - Anatomischer Anzeiger*, *201*, 91-98. https://doi:10.1016/j.aanat.2015.05.006 | | | | | | |
| To evaluate the efficacy of a stereoscopically presented 3D neuroanatomical model to a large group of students and assess students’ retention of anatomical knowledge | Randomied Controlled Study | Second year medical students (N=169) at the University of Mainz, Germany  [females=106; males= 63] | 3D Group:  Pre-recorded audio lecture on the third ventricle & 3D animated tour of the third ventricle with DextroBeam  (N=89) | Control Group/2D Group:  Pre-recorded audio lecture on the third ventricle & 2D PowerPoint Presentation (N=80) | MCQ Exam:  Consisted of 10 questions related to the topographical anatomy of the third ventricle; students had 10 minutes to complete it  Evaluation Questionnaire:  Consisted of four questions and asked participants for baseline characteristics such as age and sex | Mean score for the MCQ test was higher for the 3D group (mean score= 5.45) than the 2D Group (mean score= 5.19), however, there was no statistically significant differences between the two groups.  Due to absence of a large sample size (N=169; 3D group: N=89, 2D group: N=20), the superiority of the 3D teaching over the 2D teaching cannot be determined accurately.  Student responses on the evaluation questionnaire revealed that scores for the 3D teaching were statistically significantly higher than for 2D teaching for all four questions. |
| **Does 3D stereoscopy support anatomical education?**  Bernard, F., Richard, P., Kahn, A., & Fournier, H. (2020). Does 3D stereoscopy support anatomical education?. *Surgical And Radiologic Anatomy*, *42*(7), 843-852. https://doi:10.1007/s00276-020-02465-z | | | | | | |
| To investigate whether a 3D stereoscopic instruction video on the Circle of Willis could improve learning over a 2D video. | Prospective Randomized Controlled Study | Second year medical students (N=175) at the University of Angers, France | 3D Group:  3D stereoscopic video  (N=91) | 2D Group:  Non-stereoscopic video (N=84) | Pre-course evaluation:  Consisted of 27 right or wrong items, one SAQ and 2 MCQs; these questions aimed to assess fundamental knowledge, spatial relationships, and clinical reasoning; was completed by all students before watching the video they were assigned to; lasted 10 minutes  Post-course evaluation:  Identical to the pre-course evaluation; was completed by students one month after viewing the video they were assigned to  Satisfaction survey:  Completed by the 3D Group only to evaluate their 3D experience; consisted of 5-point Likert scale 12 statements | Both groups achieved higher scores in the post-course evaluation than in the pre-course evaluation except for question 7 for the 3D group.  There was a statistically significant difference between the scores of the two groups as students from the 3D group scored higher than students from the 2D group in the anatomical relations and clinical reasoning sections (p=0.01)  Student responses from the satisfaction questionnaire showed that overall, students had positive reviews for most questions (Q1-Q7 & Q11-A12). |
| “Visualization matters” – stereoscopic visualization of 3D graphic neuroanatomic models through AnaVu enhances basic recall and radiologic anatomy learning when compared with monoscopy  Yohannan, D.G., Oommen, A.M., Kumar, A.S. Devanand, S., UT, M.R., Sajan, N., Thomas, N.E., Anzer, N., Raju, N.K., Thomas, B., Rajan, J.E., Govindapillai, U.K., Harish, P., Kapilamoorthy, T.R., Kesavadas, C. and Sivaswamy, J. (2024). “Visualization matters” – stereoscopic visualization of 3D graphic neuroanatomic models through *AnaVu* enhances basic recall and radiologic anatomy learning when compared with monoscopy. *BMC Med Educ* **24**, 932. <https://doi.org/10.1186/s12909-024-05910-4> | | | | | | |
| To assess the utility of the AnaVu tool (stereoscopic/monoscopic tool) compared to conventional mehods. | Three-limb Randomized Controlled Study | First-year medical students (N=152) | Stereoscopic group:  Students received a 20-minute demonstration on the brainstem lesson module via AnaVu in stereoscopic mode.    Monoscopic group:  Students received a 20-minute demonstration on the brainstem lesson | Control group:  Students were taught neuroanatomy via white board drawn diagrams. | Pre-intervention test:  Students took the pre-test before accessing their assigned resource.  Post-intervention test:  Students took the post-test after accessing their assigned resource.  Pre- and post-intervention tests lasted 20 minutes each; maximum score was 35. Pre- and post- tests consisted of four domains: 4 basic recall questions, 3 analytical questions and two diagram-based questions.  Cognitive load assessment tool:  Students answered 10 questions (3 questions on intrinsic load, 3 questions extraneous load and 3 questions on germane load) after accessing their assigned resource.  Subjective feedback: Students answered 12 5-point Likert scale questions on users’ satisfaction and how much they thought the resource reinforced their knowledge.  Survey questionnaire:  Students in the stereoscopic and and monoscopic groups completed a survey questionnaire after accessing both resources. Students answered two open-ended questions comparing the two demonstrations, commenting which demonstration they prefer and listing any disadvantages for each demonstration. | No statistically significant differences were found in the pre-test scores among the three groups (p=0.947).  All three groups scored statistically significantly higher in the post-test than in their pre-test (p<0.001).  No statistically significant differences were found in the post-test scores among the three groups (p=0.233).  No statistically significant differences were observed between the pre- and post-test scores when comparing all three groups together (p=0.226).  Domain-specific analysis showed a statistically significant difference in basic recall scores among groups, with the Stereo group scoring significantly higher than both the Mono (p = 0.03) and Control groups (p = 0.001).  Statistically significant differences were observed in radiology-based question scores, with both the Stereo and Mono groups outperforming the Control group (p < 0.001 and p = 0.046, respectively), while no significant differences were observed in analytical or diagram-based questions. |
| **Virtual reality resources** | | | | | | |
| **Immersive virtual reality as a teaching tool for neuroanatomy**  Stepan, K., Zeiger, J., Hanchuk, S., Del Signore, A., Shrivastava, R., Govindaraj, S., & Iloreta, A. (2017). Immersive virtual reality as a teaching tool for neuroanatomy. *International Forum of Allergy & Rhinology*, *7*(10), 1006-1013. https://doi:10.1002/alr.21986 | | | | | | |
| To evaluate the effectiveness, satisfaction, and motivation associated with immersive VR simulation in teaching medical students neuroanatomy | Randomized Controlled Study | First (N=34) and second year (N=32) medical students  (N=66) at Icahn School of Medicine at Mount Sinai, NY, United States | VR Group:  VR model of brain anatomy; students had a 10-minute VR study comprised of a 5-minute 3D video showing key anatomic relationships & 5 minutes of a fully immersive VR experience, and a 10-minute session with control study materials  *Study control materials were not defined by the authors | Control Group:  20-minute independent study with control study materials  *Study control materials were not defined by the authors | Demographic Survey:  Obtained information about baseline characteristics and information about students’ experience with video games, visuospatial activities, and VR-head sets  Pre-intervention Quiz:  Consisted of 10 questions and was completed before students were exposed to their assigned exposure; questions included MCQs and identification questions on the ventricular system, arterial blood supply and brainstem  Post-intervention Quiz:  Consisted of 30 questions and was completed by all students after their assigned exposure; questions included MCQs and identification questions on the ventricular system, arterial blood supply and brainstem  Retention Quiz:  Consisted of 15 questions and was completed by 65 students independently without consulting outside resources 8 weeks after their exposure; questions included MCQs and identification questions on the ventricular system, arterial blood supply and brainstem  *Questions on pre-intervention, post-intervention and retention quizzes were not identical  Subjective Experience Survey:  Administered to ask participants to rate how useful, enjoyable, engaging, and easy their assigned study tools were  Instructional Material Motivation Survey (IMMS): Aimed to assess subjective user experience and assess learners’ motivation | No statistically significantly differences were observed between the VR and Control groups for their scores on the pre-intervention, post-intervention, or retention quizzes  Second year medical students scored significantly higher than first year medical students in all three quizzes (p< 0.01).  Responses from the subjective user survey showed that students from the VR group thought that their assigned learning tools were more engaging p<0.01), enjoyable (p<0.01) and useful for learning p<0.01) and were more likely to recommend such study material to other students (p<0.01)  Student scores from the VR group on the IMMS were significantly higher (p<0.01) than scores from the control group. |
| **Immersive and interactive virtual reality to improve learning and retention of neuroanatomy in medical students: a randomized controlled study**  Ekstrand, C., Jamal, A., Nguyen, R., Kudryk, A., Mann, J., & Mendez, I. (2018). Immersive and interactive virtual reality to improve learning and retention of neuroanatomy in medical students: a randomized controlled study. *CMAJ Open*, *6*(1), E103-E109. https://doi:10.9778/cmajo.20170110 | | | | | | |
| To examine the impact of immersive virtual-reality neuroanatomy training and compare it to traditional paper-based methods | Randomized Controlled Study | First (N=41) and second (N=23) year medical students (N=64) at the University of Saskatchewan, Canada | VR Group:  (N=31)  Virtual reality learning material illustrating the following set of brains structures: caudate, putamen, globus pallidus, thalamus, ventricles, amygdala, hippocampus, lateral corticospinal tract, spinothalamic tract. Virtual reality brain was presented via a headset using two handheld remotes; participants had 12 minutes of study time to memorise spatial relationships | Paper-based Group:  (N=33) Booklet containing 15 colored figures and corresponding labels for the same set of brain structures presented in the VR group. Colored figures were obtained from the Blumenfeld’s Neuroanatomy Through Clinical Cases, textbook; participants had 12 minutes of study time to memorise spatial relationships | Pre-test MCQ:  Consisted of 12 questions that assessed students’ current knowledge of neuroanatomy; lasted 10 minutes  Post-test MCQ:  Consisted of 12 questions that assessed students’ current knowledge of neuroanatomy; lasted 10 minutes  7-day post-intervention test:  A 20-minute test administered 5-9 days after students were exposed to their assigned resource  Satisfaction Survey:  25 questions assessing participants’ learning experience satisfaction | No statistically significant differences were observed between student scores from the two groups for the pre and two post-intervention tests  94% of students from the VR group strongly agreed and agreed that VR should be used in the curriculum in contrast to 33% of students from the paper-based group.  Students from both groups reported feeling more confident to engage in learning neuroanatomy after been exposed to their assigned resource, however, students from the VR group reported higher confidence rates. |
| Enhancing Medical Students' Anatomy Memory through Virtual Reality Dissection Simulations  T, Richard. and R, Rajakumari. (2023). Enhancing Medical Students' Anatomy Memory through Virtual Reality Dissection Simulations. ICSES, pp. 1-8, https://doi.org/10.1109/ICSES60034.2023.10465581 | | | | | | |
| To evaluate the short-term and long-term influence of 3D-VR technology on learning outcomes, when compared to traditional teaching models. | Randomized Controlled Study | First- and second-year medical students (N=66)  * The exact number of first- and second-year medical students was not specified by the authors. | Experimental group (N=33):  Participants received a five-minute lesson to become familiar with iPads loaded with the VR application. Subsequently, participants accessed a range of neuroanatomical structures (basal ganglia, thalamus, hippocampus, corticospinal and spinothalamic tracts), in the VR application. | Control group (N=33):  Used traditional paper methods that showed the same neuroanatomical structures present in the VR application. | Pre-test:  All participants took a pre-test to assess baseline knowledge prior accessing their assigned resource.  Post-test:  All participants took a post-test after accessing their assigned resource to test if their performance was improved.  Long-term knowledge retention test:  Participants undertook a long-term retention test four weeks after accessing their assigned resource.  All three tests taken by the participants followed the objective structured practical examination (OSPE) framework. All tests aimed to evaluate participants’ ability to conceptualize spatial relationships among anatomical structures in 3D. Each test consisted of 13 test questions and 9 control questions.  Focus groups:  Participants from the intervention group participated in focus groups to share their impressions of using the 3D-VR application. | No statistically significant differences were observed in the pre-test scores of male (p=0.08) and female (p=0.07) participants in the control and intervention groups individually.  For the control group, a statistically significant difference was observed between the pre- and post-test scores of male (p<0.001) and female (p=0.01) participants. Both male and female participants scored lower in their post-test scores when compared to their pre-test scores.  For the intervention group, a statistically significant difference was observed between the pre- and post-test scores of males (p<0.001) and female (p=0.03) participants. Both male and female participants scored higher in their post-test scores when compared to their pre-test scores.  For the long-term knowledge test, a statistically significant difference was observed between the first post-test and the long-term post-test in male (p<0.01) and female (p=0.02) participants within the control group. Both male and female participants scored slightly higher in the long-term post-test than in the first-post-test.  For the long-term knowledge test, a statistically significant difference was observed between the first post-test and the long-term post-test in male (p<0.01) and female (p=0.02) participants within the intervention group. Both male and female participants scored slightly higher in the long-term post-test than in the first-post-test. |
| **Augmented reality resources** | | | | | | |
| **Learning anatomy via mobile augmented reality: Effects on achievement and cognitive load**  Küçük, S., Kapakin, S., & Göktaş, Y. (2016). Learning anatomy via mobile augmented reality: Effects on achievement and cognitive load. *Anatomical Sciences Education*, *9*(5), 411-421. https://doi:10.1002/ase.1603 | | | | | | |
| To determine the effects of learning anatomy via mAR on medical students’ academic achievement and cognitive load. | Mixed-methods study | Second year medical students (N=70) at Ataturk University, Turkey | Experimental Group: Participants were taught by traditional presentation material such as 2D pictures, graphs, and text and had access to the MagicBook mobile Augmented Reality (mAR) App that focused on the ascending and descending pathways; MagicBook consisted of 6 3D video animations, 3D human anatomy model and two diagrams which were supplementary material for the experimental group only  Participants used MagicBook to review the teaching material  (N=34) [females=16; males=18] | Control Group:  Participants were taught by traditional presentation material such as 2D pictures, graphs, and text  Participants used a traditional textbook to review the teaching material  (N=36)  [females=20; males=16] | Pre-test:  To assess level of neuroanatomy knowledge  Post-test:  Consisted of 1) Cognitive Load Test and 2) Academic Achievement Test  Cognitive Load Test:  9-point scale test that aimed to measure each students’ cognitive load while studying the spinal pathways  (1=extremely low; 9=extremely high)  Academic  Achievement Test:  Consisted of 30 MCQ questions that aimed to measure students’ academic achievement  Interviews:  Were conducted with students from the experimental group | No statistically significant differences were found in the pre-test scores of the experimental and control groups (p=0.821).  Students from the experimental group were statistically significant more successful (p<0.05) than students from the control group and were statistically significantly found to have lower cognitive load compared to students from the control group (p<0.05).  Most students (79%) reported that the mAR app facilitated them to learn the subject  Interview responses revealed that students believed that the mAR app enhanced their academic achievement  Most students reported that the mAR app reduced their cognitive load |
| **Neuroanatomy Learning: Augmented Reality vs. Cross-Sections**  Henssen, D., van den Heuvel, L., De Jong, G., Vorstenbosch, M., van Cappellen van Walsum, A. M., Van den Hurk, M. M., Kooloos, J., & Bartels, R. (2020). Neuroanatomy Learning: Augmented Reality vs. Cross-Sections. *Anatomical sciences education*, *13*(3), 353–365. <https://doi.org/10.1002/ase.1912> | | | | | | |
| To investigate the differences on test scores, cognitive load, and motivation after neuroanatomy learning using AR applications or using cross-sections of the brain | Randomized Controlled Study | First year medical and biomedical sciences students (N=31) at Radboud University in Nijmegen, Netherlands  *Individual number of medical students and biomedical sciences students was not specified | GreyMapp-AR Group:  Practical Assignment 1:  Students received an overview of the anatomy of the human brain  Practical Assignment 2:  Students studied subcortical structures using the GreyMapp Augmented Reality application | Control Group:  Practical Assignment 1:  Students received an overview of the anatomy of the human brain  Practical Assignment 2:  Students studied subcortical structures using cross-sections (anatomical drawings of transverse sections of the human brain) | Mental Rotation Test:  Completed by students to assess their spatial ability; students could score a maximum score of 24 points  Pre-test:  Completed by students before becoming exposed to their assigned resource to measure their neuroanatomy knowledge  Post-test:  Completed by students after becoming exposed to their assigned resource to measure their neuroanatomy knowledge  Pre and post-tests’ content was identical and consisted of EMQs and MCQs that were completed before students needed to name structures on cross-sectional images  Cognitive Load Questions:  Students needed to answer three questions on a 6-point Likert scale to measure their three dimensions of cognitive load after each of the two practical assignments  Instructional Material Motivation Survey (IMMS): Aimed to measure the students’ motivational reactions to self-directed instructional materials; was competed by 30 students only | No statistically significant differences were observed between the pre and post-test scores of the two groups  Post-test scores from both groups were significantly higher than their pre-test scores; post-test scores for the control group were significantly higher than post-test scores for the GreyMapp-AR group  Statistical analysis of the three components of the post-test showed that students from the control groups scored significantly better on the third part of the test (cross-sections) than students from the GreyMappAR group.  Total cognitive load scores were higher for both groups after students had both practical assignments (p=0.039)  No statistically significant differences were observed between the two groups for the IMMS  Qualitative student responses from the focus group showed that the GreyMapp-AR could be used as an adjunct learning tool but should never replace traditional teaching methods and resources such as atlases or prosections.  Students from the GreyMapp-AR group were frustrated with the lack of detail and learning objective alignment present in the AR resource while students from the control group reported having difficulties with questions that tested for 3D insights.  Students from the GreyMapp-AR group listed a couple of disadvantages associated with the app  Students expressed that a combination of both resources at different time frames might be beneficial for their neuroanatomy learning |
| Comparing the effectiveness of augmented reality and anatomical atlases in student preparation for neuroanatomy dissection  Zeedzen-Scheffers, I., Karstens, J., van den Hurk, M., Henssen, D. and Boer, L.L. (2024). Comparing the effectiveness of augmented reality and anatomical atlases in student preparation for neuroanatomy dissection. *Sci Rep* **14**, 24939 (2024). https://doi.org/10.1038/s41598-024-76379-w | | | | | | |
| To examine the effectiveness of using an AR application versus anatomical atlases in preparing students for their neuroanatomy prosection-based practicals | Randomized Controlled Study | First year (N=5) and second year (N=23) medical and biomedical sciences students  Total number of participants N=28  *The exact number of medical students and biomedical sciences students in their first and second year was not specified. | AR Group:  Had access to the GreyMapp AR resource to complete their preparatory assignment prior attending their body donor-based education.    Preparatory Assignment 1:  Students received an overview of the anatomy of the human brain | Control Group:  Had access to an anatomical atlas resource to complete their preparatory assignment prior attending their body donor-based education. | Mental rotation test:  Students undertook an MRT to assess their spatial abilities.  Cognitive engagement test:  Consisted of four 5-point Likert scale statements. Students took two cognitive engagement tests, one after their preparatory assignment and one after students had their neuroanatomy session with prosections.  Pre-test:  It was undertaken by students before becoming exposed to their assigned intervention (either GreyMapp AR resource or Sobotta anatomical atlas).  Pre-test consisted of nine MCQs, 11 dichotomous questions, and five structures that needed to be identified in cross-sections. Maximum score points that could be achieved= 35.  Post-test:  The post-test had the same format as the pre-test; however, some questions were different from the ones in the pre-test. | No statistically significant differences were observed in the MRT scores of the AR and control groups (p=0.09).  Post-test scores were higher than pre-test scores for both groups (p<0.001).  No statistically significant differences were observed in the pre- and post-test scores of two groups (p=0.35). |
| AEducAR3.0: An Exciting Hybrid Educational Platform for a Comprehensive Neuroanatomy Learning  Cercenelli, L., Stradiotti, S., Bortolani, B., Tarsitano, A., Manzoli, L.,.(2024). AEducAR3.0: An Exciting Hybrid Educational Platform for a Comprehensive Neuroanatomy Learning. In: De Paolis, L.T., Arpaia, P., Sacco, M. (eds) Extended Reality. XR Salento 2024. Lecture Notes in Computer Science, vol 15028. Springer, Cham. https://doi.org/10.1007/978-3-031-71704-8_10 | | | | | | |
| To explore the effectiveness of the AEducAR3.0 hybrid platform which combines AR with 3D printed models in allowing students to study neuroanatomy at different learning levels (notional learning, notional learning in context and topographical learning). | Not specified | Second-year medical students (N=70) | AR Group: Had access to the AEducAR3.0 platform | No comparator present | Self-assessment quiz:  Students took a quiz after experiencing each of the three types of learning levels. The maximum score that could have been achieved for all three learning levels as a whole was 16. | 51% of the students scored higher than the sufficient threshold of 9.6, with a median overall score of 10 out of 16 when all three learning levels were considered as a whole.  The quiz results for each learning level showed that the testing phases were well balanced in terms of their difficulty across all three levels of learning.  The mean rate of correct answers for learning level one, two and three was 60% ±7%, 70% ±4% and 58% ±5%, respectively).  Students achieved a higher correct response rate for the quiz of learning level two when compared to the correct response rate (70% ±4%) for the quizzes of learning levels 1 and 3 (60% ±7% and 58% ±5%, respectively) (Learning level 1 vs Learning level 2: p=0.04; Learning level 2 vs Learning level 3: p=0.02). |
| Creating a neuroanatomy education model with augmented reality and virtual reality simulations of white matter tracts  Gurses ME, Gökalp E, Gecici NN, Gungor A, Berker M, Ivan ME, Komotar RJ, Cohen-Gadol AA, Türe U. 2024. Creating a neuroanatomy education model with augmented reality and virtual reality simulations of white matter tracts. J Neurosurg.141(3):865-874. <https://doi.org/10.3171/2024.2.JNS2486> | | | | | | |
| To investigate the effectiveness of teaching neuroanatomy with AR and VR resources when cadaveric dissection is not available. | Not specified | Neurosurgery residents (N=40) & second-year medical students (N=200) | Neurosurgery residents:  Accessed VR-Based 3D Models    Medical Students:  Accessed AR-based 3D Models | No comparator was present | Pre-test:  Consisted of 10 questions and was undertaken by participants prior accessing their assigned resource.  Post-test:  Consisted of 10 questions and was undertaken by participants after accessing their assigned resource.  Neurosurgery residents who accessed the VR resource completed a 4-point Likert survey to evaluate their experience while using the VR resource. | Neurosurgery residents scored higher than medical students in the pre-test (7.5/10 vs 4.8/10).  Both neurosurgery residents and medical students scored statistically significantly higher in their post-test scores.  [Pre-test vs Post-test scores: Neurosurgery residents: 7.5/10 vs 9.7/10, p<0.001; Medical students: 4.8/10 vs 8.7/10, p<0.001). |
| **Mixed reality resource** | | | | | | |
| **Assessing the difference in learning gain between a mixed reality application and drawing screencasts in neuroanatomy**  Pickering, J., Panagiotis, A., Ntakakis, G., Athanassiou, A., Babatsikos, E., & Bamidis, P. (2022). Assessing the difference in learning gain between a mixed reality application and drawing screencasts in neuroanatomy. *Anatomical Sciences Education*, *15*(3), 628-635. https://doi:10.1002/ase.2113 | | | | | | |
| To explore the impact of a MR resource focusing on the sensory and motor spinal pathways in comparison to a drawing screencasts multimedia video resource which was a pre-existing resource already embedded in the medical curriculum | Quasi-randomized control trial | Second year medical students (N=200) at the University of Leeds, UK | MR Resource or Anatomy Drawing Screencast | No comparator was present | Pre-test: was undertaken by students before becoming exposed to their assigned intervention (either MR or anatomy drawing screencast resource); students answered 10 MCQs on the basic anatomical principles of the pathways, 1 clinical scenario SAQ  Post-test:  It was the same as the pre-test and it was undertaken by students after their exposure to their assigned intervention (either MR or anatomy drawing screencast resource) | The percentage scores of students for the MCQ and SAQ sections and overall score were statistically significantly higher in the post-test irrespective of the resource the students have been exposed to (MR: p<0.001; Screencast: p<0.001).  When absolute and normalizing gains were applied to the data, it was shown that the only statistically significant increase in learning gain was observed in the MCQ section of the post-test for the screencast group (absolute gain, p < 0.003; normalizing gain, p< 0.01) |

AR: Augmented Reality

CAL: Computer-assisted learning

mAR: Mobile augmented reality

MCQ: Multiple-choice question

MR: Mixed Reality

MRT: Mental Rotation Test

PICO(s): Patient/Population, Intervention, Comparison and Outcomes and Study

R-SPQ-2F: The Revised Two Factor Study Process Questionnaire

SAQs: Short-answer questions

VR: Virtual Reality

# Supporting Information

# DataS1
